# To tune or not to tune, a case study of ridge logistic regression in small or sparse datasets

**DOI:** 10.1186/s12874-021-01374-y

**Published:** 2021-09-30

**Authors:** Hana Šinkovec, Georg Heinze, Rok Blagus, Angelika Geroldinger

**Affiliations:** 1 Section for Clinical Biometrics, Center for Medical Statistics, Informatics and Intelligent Systems, Medical University of Vienna, Spitalgasse 23, 1090 Vienna, Austria; 2 Institute for Biostatistics and Medical Informatics, University of Ljubljana, Ljubljana, Slovenia

**Keywords:** Calibration slope, Firth’s correction, Mean squared error, Penalized logistic regression, Ridge regression, Shrinkage, Tuning

## Abstract

**Background:**

For finite samples with binary outcomes penalized logistic regression such as ridge logistic regression has the potential of achieving smaller mean squared errors (MSE) of coefficients and predictions than maximum likelihood estimation. There is evidence, however, that ridge logistic regression can result in highly variable calibration slopes in small or sparse data situations.

**Methods:**

In this paper, we elaborate this issue further by performing a comprehensive simulation study, investigating the performance of ridge logistic regression in terms of coefficients and predictions and comparing it to Firth’s correction that has been shown to perform well in low-dimensional settings. In addition to tuned ridge regression where the penalty strength is estimated from the data by minimizing some measure of the out-of-sample prediction error or information criterion, we also considered ridge regression with pre-specified degree of shrinkage. We included ‘oracle’ models in the simulation study in which the complexity parameter was chosen based on the true event probabilities (prediction oracle) or regression coefficients (explanation oracle) to demonstrate the capability of ridge regression if truth was known.

**Results:**

Performance of ridge regression strongly depends on the choice of complexity parameter. As shown in our simulation and illustrated by a data example, values optimized in small or sparse datasets are negatively correlated with optimal values and suffer from substantial variability which translates into large MSE of coefficients and large variability of calibration slopes. In contrast, in our simulations pre-specifying the degree of shrinkage prior to fitting led to accurate coefficients and predictions even in non-ideal settings such as encountered in the context of rare outcomes or sparse predictors.

**Conclusions:**

Applying tuned ridge regression in small or sparse datasets is problematic as it results in unstable coefficients and predictions. In contrast, determining the degree of shrinkage according to some meaningful prior assumptions about true effects has the potential to reduce bias and stabilize the estimates.

**Supplementary Information:**

The online version contains supplementary material available at 10.1186/s12874-021-01374-y.

## Background

In medical research, logistic regression is commonly used to study the relationship between a binary outcome and a set of covariates. For a dataset with similar prevalence of the two outcome levels and sufficient sample size, the maximum likelihood estimation of the regression coefficients facilitates inference, i.e. interpretability of effect estimates, as well as accuracy of predictions given the covariates. Thus, maximum likelihood logistic regression may be used for explanation or prediction, depending on context. These attractive properties of the maximum likelihood logistic regression, however, vanish when the sample size is small or the prevalence of one of the two outcome levels (for some combination of exposure) is low, yielding coefficient estimates biased away from zero and very unstable predictions that generalize poorly on a new dataset from the same population [1, 2].

In theory, a straightforward approach to alleviate the problem would be to apply penalized maximum likelihood logistic regression: a penalty term that is added to the log likelihood function provides shrinkage of the coefficients towards zero, hereby decreasing the variance of the maximum likelihood estimates and stabilizing the predictions by pulling them towards the observed event rate [3]. A common way of shrinkage is by ridge logistic regression where the penalty is defined as minus the square of the Euclidean norm of the coefficients multiplied by a non-negative complexity parameter *λ*. The multiplier *λ* controls the strength of the penalty, i.e. amount of shrinkage towards zero. According to the idea of the bias-variance trade-off, the expected prediction error can be decomposed into the three components bias, variance and irreducible error [4]. Hence, the goal in ridge regression is to find the value of *λ* that balances the model between underfitting and overfitting, producing generalizable results [5]. As compared to the maximum likelihood estimation the resulting coefficients may achieve lower mean squared errors (MSE) but are usually biased towards zero, therefore conventional inference by hypothesis tests and confidence intervals based on standard errors is difficult [6]. A further complication for inference arises from the estimation of *λ*, which is often performed on the same data set by cross-validation, as its sampling variability contributes to the uncertainty in the regression coefficients.

Tuned ridge logistic regression has been extensively investigated in simulation studies and was commonly found to perform well for low dimensional settings in terms of small MSE of coefficients and predictions [2, 7, 8]. However, one should not expect that penalization can overcome the problem of insufficient sample sizes when developing prediction models [9]. Indeed, there has been evidence that ridge regression is sensitive to small or sparse data situations, yielding poor performance in individual datasets [10–13]. Recent recommendations, therefore, advise caution when using ridge logistic regression for developing prediction models in case of low sample size or low events per variable ratio and call for more research investigating the impact of specific combinations of shrinkage and tuning methods [11]. While in theory there always exists some value of *λ* for which ridge regression outperforms maximum likelihood estimation in terms of the MSE of predictions [14], choosing *λ* adequately in datasets that suffer from large random sampling variation is difficult. For such datasets tuning procedures based on out-of-sample prediction performance might fail to approximate the U-shaped curve arising from the bias-variance trade-off and result in an arbitrary choice of *λ* that either equals the smallest or the largest value of the pre-specified range of values. This will yield large variability of tuned solutions and consequently, very unstable estimates [13].

We assume that larger variability of calibration slopes in small or sparse datasets as compared to Firth’s correction [11] is closely related to tuning and not to ridge regression as a shrinkage method per se. Therefore, in the present paper we investigate the performance of different commonly used approaches to tune ridge logistic regression in a low-dimensional sparse data setting by means of a simulation study. We also include ridge regression with pre-specified *λ*, which is interpretable as semi-Bayesian analysis with a normal prior centered at zero [1, 15, 16], and Firth’s correction [17] in our comparison, as these approaches were proposed for similar settings [7, 11, 12, 18] and do not suffer from the convergence issues that may occur in maximum likelihood estimation [19]. We structured the paper accordingly: in the following section we introduce Firth’s correction and ridge logistic regression and describe different ways to choose the complexity parameter *λ* in ridge regression. We then illustrate the problems which might arise with tuning in sparse data situations. Subsequently, we present the setup and report the results from our simulation study with respect to the accuracy of coefficients and predictions. Further on, we perform an analysis of a real data example by fitting ridge regression and Firth’s correction models. Finally, we summarize our main findings.

## Methods

Let *y*_*i*_ ∈ {0, 1}, *i* = 1, …*N*, be a realization of a binary outcome variable *Y*, where *y*_*i*_ = 1 denotes an event occurring in the *i*-th observation. The logistic regression model associates *y*_*i*_ to a set of corresponding covariate values *x*_*i*_ = (1, *x*_*i*1_, …, *x*_*iK*_), *K* < *N*, by assuming

$$ {\pi}_i=P\left(Y=1|{\mathbf{x}}_{\mathbf{i}}\right)=\frac{1}{1+\exp \left(-{\beta}_0-{\beta}_1{x}_{i1}-\dots -{\beta}_K{x}_{iK}\right)}, $$

where β_0_ is an intercept and β_*k*_, *k* = 1, …, *K*, are regression coefficients. The parameters **β** = (β_0_, β_1_, …β_*K*_) of the model can be estimated by the maximum likelihood method, maximizing the log-likelihood function

$\ell \left(\boldsymbol{\upbeta} \right)=\sum \limits_{i=1}^N\left({y}_i\log {\pi}_i+\left(1-{y}_i\right)\log \left(1-{\pi}_i\right)\right),$ using an iterative algorithm [20].

### Firth’s correction

Maximum likelihood estimation is asymptotically unbiased, however, in situations when data are small or sparse coefficient estimates become biased away from zero and very unstable or may even not exist [19]. To reduce the bias of maximum likelihood estimates, Firth [17] proposed to penalize the likelihood function by Jeffreys’ invariant prior so that the penalized log-likelihood becomes

$$ {\ell}_{FC}^{\ast}\left(\boldsymbol{\upbeta} \right)=\ell \left(\boldsymbol{\upbeta} \right)+\frac{1}{2}\log \left|\boldsymbol{I}\left(\boldsymbol{\upbeta} \right)\right|, $$

where ***I***(**β**) is the Fisher information matrix evaluated at **β**. Since the intercept is included in the penalty term, the average predicted probability may not equal the observed event rate but is instead biased towards one-half. To correct for this bias that may become especially apparent in situations with unbalanced outcome, Puhr et al. [7] proposed a simple modification, Firth’s logistic regression with intercept-correction (FLIC) that alters the intercept such that average predicted probabilities become equal to the observed event rate.

### Ridge regression

In ridge regression coefficients are constrained by the square of the Euclidean norm of the coefficients, i.e. the penalized log-likelihood reads

$$ {\ell}_{ridge}^{\ast}\left(\boldsymbol{\upbeta} \right)=\ell \left(\boldsymbol{\upbeta} \right)-\frac{\lambda }{2}\sum \limits_{k=1}^K{\beta}_k^2, $$

where the positive complexity parameter *λ* controls the amount of shrinkage towards zero. The intercept *β*_0_ is excluded from the penalty term, yielding an average predicted probability equal to the observed event rate. Unlike Firth’s correction, ridge regression is not invariant to linear transformation of the design matrix. Therefore, to facilitate interpretation and ensure that coefficients are represented on the same scale, suitable standardization of covariates is required, usually to zero mean and unit variance.

#### Tuning procedures

To select the complexity parameter *λ*, generally, a sequence of *λ* values is pre-specified and the corresponding set of models is evaluated. The optimized *λ*^∗^ is the one that produces the model minimizing the expected out-of-sample prediction error, often estimated by cross-validation. The out-of-sample prediction error may be defined in different ways [3, 21–23], e.g. as

- deviance (*D*) [3]
  $$ D=-2\sum \limits_{i=1}^N\left({y}_i\log {\hat{\pi}}_{\left(-i\right)}+\left(1-{y}_i\right)\log \left(1-{\hat{\pi}}_{\left(-i\right)}\right)\right), $$
- generalized cross-validation (*GCV*) [21, 23]
  $$ GCV=\frac{N\cdot D}{{\left(N-d{f}_e\right)}^2}, $$

where *df*_*e*_ are the effective degrees of freedom, $d{f}_e=\mathrm{trace}\left(\frac{\partial^2\ell }{\partial^2\boldsymbol{\upbeta}}\left(\hat{\boldsymbol{\upbeta}}\right){\left(\frac{\partial^2{\ell}_{ridge}^{\ast }}{\partial^2\boldsymbol{\upbeta}}\left(\hat{\boldsymbol{\upbeta}}\right)\right)}^{-1}\right)$,

- classification error (*CE*) [3]
  $$ CE=\frac{1}{N}\sum \limits_{i=1}^N\left({y}_iI\left({\hat{\pi}}_{\left(-i\right)}<c\right)+\left(1-{y}_i\right)I\left({\hat{\pi}}_{\left(-i\right)}>c\right)+\frac{1}{2}I\left({\hat{\pi}}_{\left(-i\right)}=c\right)\right), $$

with *I* denoting an indicator function and *c* some cut-off, usually set to 1/2. Since in datasets with unbalanced outcomes *c* = 1/2 would assign most of observations to the more frequent outcome level Blagus and Lusa [10] advised to set *c* equal to the marginal event rate instead.

In the definitions above ${\hat{\pi}}_{\left(-i\right)}$ is the event probability estimate for the *i*-th observation computed from the model where that observation has been left out from estimation of the model parameters. Alternatively, 10-fold cross-validation may be used to speed-up computations, however, this produces different optimized *λ*^∗^ values for different combinations of fold assignments to observations. To stabilize the selection of *λ*^∗^, 10-fold cross-validation may be repeated several times, and a particular quantile *θ* of the values obtained may be used [2, 24].

Alternatively, to avoid resampling, *λ* may be tuned by using the Akaike’s information criterion (AIC) [6, 25], where

$$ \mathrm{AIC}=-2\ell \left(\hat{\boldsymbol{\upbeta}}\right)+2d{f}_e. $$

#### Pre-specifying the degree of shrinkage

Mathematically, ridge regression is identical to Bayesian analysis with zero-centered univariate normal priors imposed on the coefficients [1]. The variance *v*_*prior*_ of these priors is inversely proportional to *λ*. If the priors for the coefficients are assumed to have different variances, this translates into a penalty equal to the weighted sum of squared coefficients with different weights for each coefficient. In the Bayesian analysis approach suggested by Sullivan and Greenland [15] the degree of shrinkage is not determined by tuning but is instead based on some prior assumptions about covariates’ odds ratios that can be easily converted into *v*_*prior*_. The prior variance *v*_*prior*_ can be obtained from a plausible (usually 95%) prior interval for a covariate’s odds ratio that has to be specified according to some background assumptions. In a particular setting Sullivan and Greenland [15] considered as plausible the 95% odds ratio interval ranging from 1/4 to 4 which translates to *v*_*prior*_ = 1/2. However, if one wishes to avoid the effort of specifying prior distributions, one could apply weakly informative priors, e.g. assuming the 95% probability that the odds ratio falls between 1/16 to 16, which are still beneficial to stabilize estimates.

## Illustration

Consider the two datasets described below, each with 100 independent observations of a binary outcome, *y*_*i*_ ∈ {0, 1}, and a single covariate *x*_*i*_ ∈ {0, 1}, *i* = 1, …, 100.

**Table:** Dataset 1

		*y*	
		0	1
*x*	0	20	0
	1	71	9

**Table:** Dataset 2

		*y*	
		0	1
*x*	0	19	1
	1	71	9

**Fig. 1:**
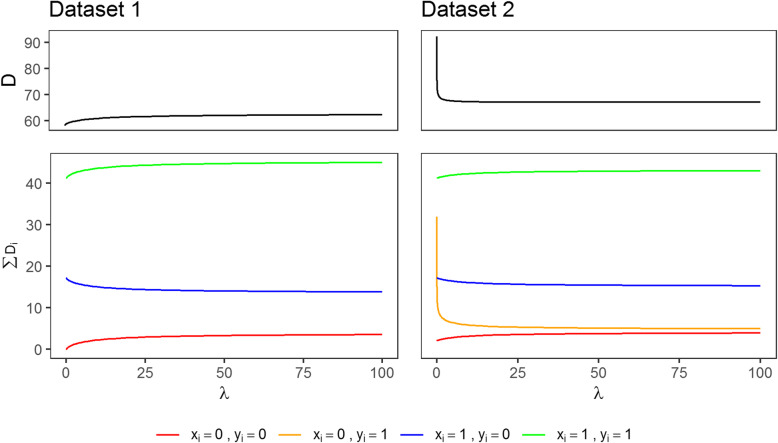
Leave one-out cross-validated deviance *D* (top) and the sum of the deviance components *D*_*i*_ for distinct observations (bottom) in dataset 1 (left) and dataset 2 (right) with respect to the complexity parameter *λ*

In dataset 1, separation occurs as there are no observations with *x*_*i*_ = 0 and *y*_*i*_ = 1. Therefore, maximum likelihood estimation yields perfect leave-one-out cross-validated predictions ${\hat{\pi}}_{\left(-i\right)}=0$ for *x*_*i*_ = 0 and such also the individual out-of-sample prediction errors equal ${D}_{x_i=0}=0$. These errors, however, increase with shrinkage (in particular, we considered a fixed sequence of 200 log-linearly equidistant *λ* values ranging from 10^(−6)^ to 100) as predicted probabilities get pulled towards 9/99 (Fig. 1). In addition, the errors increase with shrinkage for those 9 observations with event as ${\hat{\pi}}_{\left(-i\right)}=8/79=0.1$ in the maximum likelihood model and ${\hat{\pi}}_{\left(-i\right)}=0.08$ for *λ* = 100. Conversely, shrinkage reduces the errors of the 71 observations with *x*_*i*_ = 1 and *y*_*i*_ = 0 but the predicted probabilities are similar for *λ* = 100 (${\hat{\pi}}_{\left(-i\right)}=0.09$) and *λ* = 10^(−6)^ (${\hat{\pi}}_{\left(-i\right)}=0.11$), and such are the differences between the error estimates when *λ* = 100 and *λ* = 10^(−6)^ (Fig. 1). Therefore, the tuning procedure based on *D* favors the smallest of the pre-specified range of *λ* values, in our example *λ*^∗^ = 10^(−6)^. In this case, fitting ridge regression model with a standardized covariate *X* using R [26] package penalized [27] yields an estimate of *β*_1_ as large as 13.94, a consequence of data sparsity [1, 19, 28]. In contrast, FLIC (fitted by using package logistf [29]) and ridge regression with an informative prior (IP), assuming the 95% prior interval for the odds ratio of a standardized covariate ranging from 1/4 to 4, yield interpretable coefficient estimates (Table 1).

**Table 1:** Illustration: coefficients and predictions estimated by Firth’s logistic regression with intercept-correction (FLIC) and ridge regression where complexity parameter is either tuned by leave one-out cross-validated deviance *D* or set according to some informative prior (IP)

Method		FLIC	Ridge	
			*D*	IP
Dataset 1	${\hat{\beta}}_1$	1.7	13.94	1.54
	${\hat{\pi}}_{x=0}$	0.02	0	0.03
	${\hat{\pi}}_{x=1}$	0.11	0.11	0.11
Dataset 2	${\hat{\beta}}_1$	0.55	0.06	0.65
	${\hat{\pi}}_{x=0}$	0.07	0.1	0.06
	${\hat{\pi}}_{x=1}$	0.11	0.1	0.11

In dataset 2, we have one single observation with event and *x*_*i*_ = 0 for which maximum likelihood estimation falsely predicts ${\hat{\pi}}_{\left(-i\right)}=0$. While for all other observations the out-of-sample prediction errors *D*_*i*_ do not change much if applying shrinkage (for some observations *D*_*i*_ gets slightly larger and for the others slightly smaller), the error for this single observation reduces considerably with increasing shrinkage (Fig. 1). This results in *λ*^∗^ that equals the largest of the pre-specified range of values, in our example *λ*^∗^ = 100. Obviously, this overshrinks the coefficients as compared to FLIC and IP (Table 1).

Repeating the data-generating process used to generate the two datasets 500-times in which a binary covariate *X* was sampled with E(*X*) = 0.8 and the binary outcome *y*_*i*_ was drawn from a Bernoulli distribution with true event probability (1 + exp(−(−3.05 + *x*_*i*_)))^−1^, each time standardizing *X* and tuning the value of *λ*^∗^ by *D* results in a choice of *λ*^∗^ that for 42% of simulated datasets simply equals the smallest and for 38% of datasets the largest value of the pre-specified range of *λ* values. This reflects a large variability of tuned *λ*^∗^ values and consequently, very unstable coefficients with large expected MSE. The large MSE of coefficients is mostly due to data sparsity that leads to very small optimized *λ*^∗^ values and huge coefficients. It is reasonable to assume that the instability in optimized complexity parameter values stands alongside prediction performance, translating into calibration slopes of large variability (models that strongly underfit or overfit). Indeed, the median (25th and 75th percentile) of calibration slopes that were evaluated on a dataset of size 10,000, independently generated from the same distribution, was 0.07 (0.07, 18.4). In contrast, in FLIC and IP no tuning is required and plausible estimates of *β*_1_ and more stable calibration slopes are produced over 500 datasets. In particular, bias ($\mathrm{E}\left(\hat{\beta}-\beta \right)\Big)$ and MSE ($\mathrm{E}{\left(\hat{\beta}-\beta \right)}^2$) of coefficient estimate, and the median (25th and 75th percentile) of calibration slopes were −0.15, 0.64 and 0.77 (0.53, 1.77) for FLIC and −0.11, 0.45 and 0.82 (0.6, 1.6) for IP, respectively.

## Simulation study

### Design

We describe the simulation study design following recommendations by Morris [30].

#### Aims

Our aim was to systematically investigate the performance of ridge logistic regression in terms of effect estimation and prediction in low-dimensional sparse data settings where the complexity parameter *λ* was determined using different approaches and to compare it to Firth’s correction with predictions obtained by FLIC.

#### Data-generating mechanisms

To allow a fair comparison of the approaches [31], we considered a data generation scheme similar to the one described in Binder et al. [32], featuring covariates with mixed types and shapes of distributions and a complex correlation structure, similar to what an analyst is usually confronted with in biomedical prognostic studies. Covariates *X*_1_, …, *X*_15_ were obtained by applying certain transformations to variables *Z*_1_, …, *Z*_15_ sampled from a standard multivariate normal distribution with correlation matrix **Σ** (Table 2). In particular, *X*_1_, …, *X*_4_ were binary, *X*_5_ and *X*_6_ ordinal with three levels and *X*_7_, …, *X*_15_ continuous. To avoid extreme values, the continuous variables were generated from truncated distributions, where the truncation was at the third quartile plus five times the interquartile distance of the respective underlying distribution. The values of the binary outcome *y*_*i*_ were sampled from Bernoulli distributions with event probabilities (1 + exp(−*β*_0_ − *a* ∗ (*β*_1_*x*_*i*1_ + … + *β*_*K*_*x*_*iK*_)))^−1^, where *i* = 1, …, *N*, *N* ∈ {100, 250, 500}, *K* ∈ {2, 5, 10}, effect multiplier *a* ∈ {0.5, 1} for moderate and strong effects, respectively, and true regression coefficients *β*_1_, …, *β*_*K*_ defined as follows: *β*_1_ = 2.08, *β*_2_ = 1.39, *β*_3_ = *β*_4_ = 0.69, *β*_5_ = *β*_6_ = 0.35 and *β*_7_, …, *β*_10_ were chosen such that the log odds ratio between the first and the fifth sextile of the corresponding distribution was 0.69 (Table 3). An intercept *β*_0_ was determined for each simulation scenario such that the desired marginal event rate E(*Y*) ∈ {0.1, 0.25} was approximately obtained. We considered two types of analysis: one using exactly the set of real predictors used to generate the data, and one also including five noise covariates *X*_11_, …, *X*_15_ that were not associated with the outcome. We refer to this factor as ‘noise’ (absent/present). Combining the simulation parameters *N* (sample size), *K* (number of true predictors), *a* (effect multiplier), E(*Y*) (marginal event rate) and noise (absent/present) in a full factorial design resulted in 72 possible scenarios. On the request of a reviewer we added 16 more scenarios also considering *N* = 1000 and combining it with *a* ∈ {0.5,1}, *E*(*Y*) ∈ {0.1, 0.25}, *K* ∈ {5, 10} and noise (absent/present). We simulated 1000 datasets for each scenario. Table S1 in Additional file 1 shows minimum sample size required for developing a prediction model for different scenarios based on recent guidance [9, 33].

**Table 2:** Covariate structure applied in the simulation study. In particular, pairwise non-zero correlations between standard normal deviates *Z*_*k*_, the transformations defining *X*_*k*_, measurement scale of covariates *X*_*k*_ and expected value of covariates E(*X*_*k*_) are shown. [∙] denotes removal of the non-integer part of the argument and *I* is the indicator function

*Z*_*k*_	Pairwise non-zero correlations of *Z*_*k*_	Transformation defining *X*_*k*_	Scale of *X*_*k*_	E(*X*_*k*_)
*Z*_1_	*Z*_2_(0.5), *Z*_3_(0.5), *Z*_7_(0.5), *Z*_14_(0.5)	*X*_1_ = *I*(*Z*_1_ < 0.84)	binary	0.80
*Z*_2_	*Z*_1_(0.5), *Z*_14_(0.3)	*X*_2_ = *I*(*Z*_2_ < − 0.35)	binary	0.36
*Z*_3_	*Z*_1_(0.5), *Z*_4_(−0.5), *Z*_5_(−0.3)	*X*_3_ = *I*(*Z*_3_ < 0)	binary	0.50
*Z*_4_	*Z*_3_(−0.5), *Z*_5_(0.5), *Z*_7_(0.3), *Z*_8_(0.5), *Z*_9_(0.3), *Z*_14_(0.5)	*X*_4_ = *I*(*Z*_4_ < 0)	binary	0.50
*Z*_5_	*Z*_3_(−0.3), *Z*_4_(0.5), *Z*_8_(0.3), *Z*_9_(0.3)	*X*_5_ = *I*(*Z*_5_ ≥ − 1.2) + *I*(*Z*_5_ ≥ 0.75)	ordinal	1.11
*Z*_6_	*Z*_7_(−0.3), *Z*_8_(0.3), *Z*_11_(−0.5)	*X*_6_ = *I*(*Z*_6_ ≥ 0.5) + *I*(*Z*_6_ ≥ 1.5)	ordinal	0.38
*Z*_7_	*Z*_1_(0.5), *Z*_4_(0.3), *Z*_6_(−0.3)	*X*_7_ = [10*Z*_7_ + 55]	continuous	54.5
*Z*_8_	*Z*_4_(0.5), *Z*_5_(0.3), *Z*_6_(0.3), *Z*_9_(0.5), *Z*_12_(−0.3), *Z*_14_(0.5)	*X*_8_ = [max(0, 100 exp(*Z*_8_) − 20)]	continuous	146
*Z*_9_	*Z*_4_(0.3), *Z*_5_(0.3), *Z*_8_(0.5), *Z*_14_(0.3)	*X*_9_ = [max(0, 80 exp(*Z*_9_) − 20)]	continuous	112
*Z*_10_	–	*X*_10_ = [10*Z*_10_ + 55]	continuous	54.5
*Z*_11_	*Z*_6_(−0.5), *Z*_12_(0.3), *Z*_15_(0.5)	*X*_11_ = exp(0.4*Z*_11_ + 3)	continuous	21.8
*Z*_12_	*Z*_8_(−0.3), *Z*_11_(0.3), *Z*_15_(0.5)	*X*_12_ = exp(0.5*Z*_12_ + 1.5)	continuous	5.1
*Z*_13_	–	*X*_13_ = 0.01 ∗ [100(*Z*_13_ + 4)^2^]	continuous	17
*Z*_14_	*Z*_1_(0.5), *Z*_2_(0.3), *Z*_4_(0.5), *Z*_8_(0.5), *Z*_9_(0.3)	*X*_14_ = [10*Z*_14_ + 55]	continuous	54.5
*Z*_15_	*Z*_11_(0.5), *Z*_12_(0.5)	*X*_15_ = [10*Z*_15_ + 55]	continuous	54.5

**Table 3:** Regression coefficients (standardized regression coefficients) for scenarios with *K* ∈ {2, 5, 10} and *a* ∈ {0.5, 1}, where *K* is the number of true predictors in the data-generating mechanism and *a* is the effect multiplier. Regression coefficients of *X*_7_, …, *X*_10_ were chosen such that the log odds ratio between the first and the fifth sextile of the corresponding distribution was equal to 0.69. The last row shows the approximate true c-indices for those scenarios

		***K*** = 2		***K*** = 5		***K*** = 10	
		***a*** **= 1**	***a*** **= 0.5**	***a*** **= 1**	***a*** **= 0.5**	***a*** **= 1**	***a*** **= 0.5**
(Standardized) regression coefficients of true predictors	*X*_1_	2.08 (0.83)	1.04 (0.42)	2.08 (0.83)	1.04 (0.42)	2.08 (0.83)	1.04 (0.42)
	*X*_2_	1.39 (0.67)	0.69 (0.33)	1.39 (0.67)	0.69 (0.33)	1.39 (0.67)	0.69 (0.33)
	*X*_3_	–	–	0.69 (0.35)	0.35 (0.17)	0.69 (0.35)	0.35 (0.17)
	*X*_4_	–	–	0.69 (0.35)	0.35 (0.17)	0.69 (0.35)	0.35 (0.17)
	*X*_5_	–	–	0.35 (0.2)	0.17 (0.10)	0.35 (0.2)	0.17 (0.10)
	*X*_6_	–	–	–	–	0.35 (0.21)	0.17 (0.11)
	*X*_7_	–	–	–	–	0.036 (0.37)	0.018 (0.18)
	*X*_8_	–	–	–	–	0.003 (0.67)	0.002 (0.33)
	*X*_9_	–	–	–	–	0.004 (0.66)	0.002 (0.33)
	*X*_10_	–	–	–	–	0.036 (0.36)	0.018 (0.18)
Noise	*X*_11_, …, *X*_15_	0	0	0	0	0	0
True c-index		0.73	0.64	0.76	0.66	0.84	0.71

#### Methods

We analyzed each simulated dataset by fitting ridge and Firth’s logistic regression models. To obtain predictions based on Firth’s correction we applied FLIC as suggested by Puhr et al. [7]. To fit ridge regression models we first standardized covariates of each dataset to zero mean and unit variance, and then optimized the complexity parameter *λ*^∗^ over a fixed sequence of 200 log-linearly equidistant values ranging from 10^(−6)^ to 100 by using the following procedures:

- *D*;
- *GCV*;
- *CE* where the cut-off *c* was set to the observed event rate $\frac{1}{N}\sum \limits_i{y}_i.$ As *CE* is discrete in nature and has no unique optimum in *λ*, in our study *λ*^∗^ was the largest *λ* minimizing *CE*;
- *D* by 10-fold cross-validation with 50 repetitions (RCV) where *λ*^∗^ was chosen as the *θ*-th quantile of the obtained values with *θ* ∈ {0.5, 0.95} (RCV50, RCV95) [2, 24];
- AIC;
- restricting the standardized coefficients by informative (IP, *λ* = 2) and weakly informative prior assumptions (WP, *λ* = 1/2). In the simulations the degree of shrinkage was the same for all the covariates.

As a benchmark we defined two oracle models, determined by an amount of shrinkage ideal with respect to estimation of *β*_1_ (explanation oracle, OEX) and to predictions (prediction oracle, OP). For OEX *λ*^∗^ was chosen such that ${\left({\hat{\beta}}_1-{\beta}_1\right)}^2$ (or equivalently $\mid {\hat{\beta}}_1-{\beta}_1\mid$), where ${\hat{\beta}}_1$ is the ridge regression estimate of *β*_1_, was minimized; for OP, *λ*^∗^ was the one minimizing ${\sum}_{\mathrm{i}}{\left({\hat{\pi}}_i-{\pi}_i\right)}^2$, where ${\hat{\pi}}_i$ is the estimate of the *i*-th probability of *π*_*i*_. To avoid model fitting problems, all ridge regression models were fitted by data augmentation [15] in the following way: two artificial data records were added for each covariate; the values for this covariate were set to 1/*s* and to zero for other covariates, where *s* = 10 was a rescaling factor improving the approximation. Maximum likelihood estimation on this augmented dataset was then performed, specifying weights that equaled 1 for the original observations and 2*s*^2^*λ* for the pseudo-observations. We used the libraries brglm2 [34] for detecting separation, penalized [27] for performing cross-validation and logistf [29] for model fitting in R version 4.0.2 [26].

#### Estimands

The true regression coefficient *β*_1_ and the vector of true event probabilities *π* were the estimands in our study.

#### Performance measures

We evaluated the root mean squared errors (RMSE) of coefficients ${\left(\frac{1}{1000}{\sum}_{s=1}^{1000}{\left({\hat{\beta}}_{k,s}-{\beta}_k\right)}^2\right)}^{1/2}$, where ${\hat{\beta}}_{k,s},k=1,$ *s* ∈ {1, …, 1000} is the estimate of *β*_*k*_ in the *s*-th simulated dataset) and of predictions ${\left(\frac{1}{1000N}{\sum}_{s=1}^{1000}{\sum}_{i=1}^N{\left({\hat{\pi}}_{i,s}-{\pi}_{i,s}\right)}^2\right)}^{1/2}$, where ${\hat{\pi}}_{i,s}$ and *π*_*i*, *s*_ are the estimated and true event probability for the *i*-th observation in the *s*-th simulated dataset). We also evaluated c-statistics, estimated with respect to newly generated outcome, and calibration slopes evaluated on a validation dataset generated once for each scenario from the same population with a sample size *N* = 10,000. The variability of calibration slopes was assessed by median absolute deviation (MAD) of the log(slope_s_). To combine bias and variability of calibration slopes, we calculated root mean squared distances (RMSD, ${\left(\frac{1}{1000}{\sum}_{s=1}^{1000}{d}_s^2\right)}^{1/2}$), where the *s*-th distance was defined as *d*_*s*_ = log(1) − log(slope_s_), as suggested by Van Calster et al. [11]. To avoid issues with negative slopes that were rarely obtained by the methods we winsorized them at 0.01 for the calculation of RMSD. In addition, we assessed the Spearman correlation coefficients between calibration slopes and tuned complexity parameters *λ*^∗^ as well as the RMSD of calibration slopes achieved by the methods and the variability of tuned complexity parameters *λ*^∗^, expressed by median absolute deviation, over all simulated scenarios.

### Results

Among 88 simulated scenarios the prevalence of separation was ranging from zero in scenarios with moderate effects, large sample sizes and E(*Y*) = 0.25 to at most 85% in a scenario with large effects, *N* = 100, *K* = 5 and E(*Y*) = 0.1 (Table S2 and S3 in Additional file 1).

**Fig. 2:**
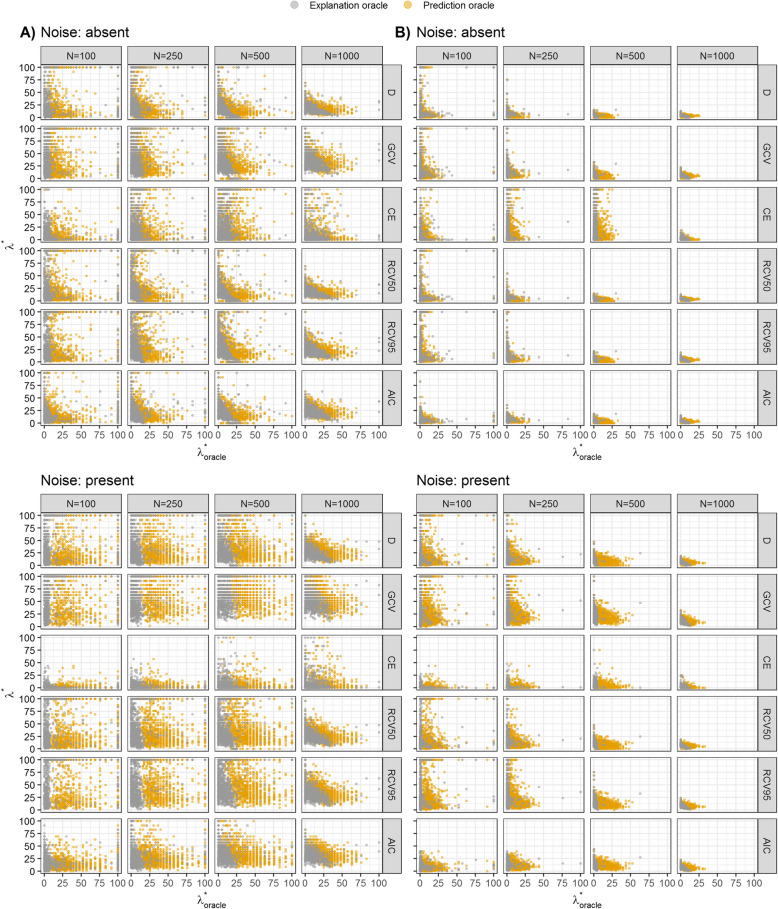
Scatter plots showing values of *λ*^∗^ obtained by optimizing different tuning criteria versus ‘optimal’ *λ*^∗^ as achieved by explanation oracle and prediction oracle over 1000 generated datasets in scenarios with the expected value of *Y*, E(*Y*) = 0.1, the number of predictors *K* = 5, noise absent or present, the sample size of *N* ∈ {100, 250, 500, 1000} considering A) moderate (*a* = 0.5) and B) strong (*a* = 1) predictors. Values of *λ*^∗^ were optimized by using different tuning criteria: *D*, deviance; *GCV*, generalized cross-validation; *CE*, classification error; RCV50, repeated 10-fold cross-validated deviance with *θ* = 0.5; RCV95, repeated 10-fold cross-validated deviance with *θ* = 0.95; AIC, Akaike’s information criterion

First, we describe the distribution of *λ*^∗^ values obtained by optimizing different tuning criteria over 1000 simulation runs and their correlations with ‘optimal’ *λ*^∗^ as achieved by OEX and OP, respectively (Fig. 2). For brevity, Fig. 2 focuses on scenarios with E(*Y*) = 0.1 and *K* = 5 only. Tuning procedures often led to large variability of selected *λ*^∗^, which was especially apparent in moderate effects scenarios. Generally, the variability was smaller when the true effects were strong, with larger *N* and *K*, i.e. the number of predictors associated with the outcome, and with more balanced outcomes. With moderate effects the methods tended to overshrink, often producing very wide distributions of optimized *λ*^∗^ values. The smallest variability of optimized values over all scenarios in terms of MAD was obtained by the AIC, followed by *CE* and RCV50. Quite some variability of *λ*^∗^ was also obtained by OP, however, the correlations between ‘optimal’ *λ*^∗^ (of both OP and OEX) and *λ*^∗^ obtained by optimizing different tuning criteria were mostly negative. OEX resulted in less variability of *λ*^∗^ and less shrinkage than OP. In IP the pre-specified *λ*^∗^ was in median very close to the one obtained by OEX.

#### Accuracy of coefficients

**Fig. 3:**
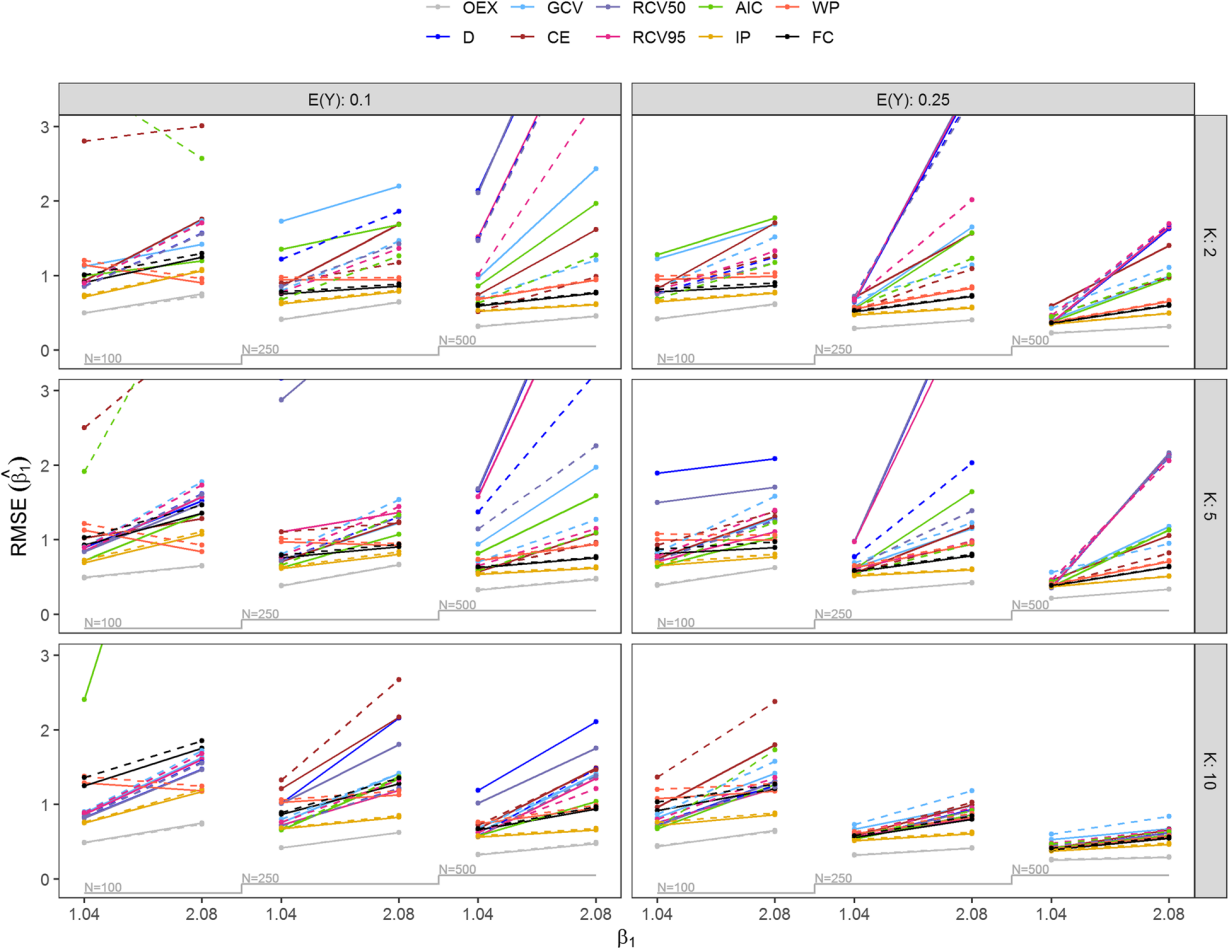
Nested loop plot of root mean squared error (RMSE) of ${\hat{\beta}}_1$ by the expected value of *Y*, E(*Y*) ∈ {0.1, 0.25}, the number of predictors *K* ∈ {2, 5, 10}, noise absent or present (full and dashed lines), the sample size *N* ∈ {100, 250, 500} and the size of true coefficients *β*_1_ ∈ {1.04, 2.08} for simulated scenarios. Due to poor performance some results lie outside the plot range. OEX, explanation oracle; *D*, deviance; *GCV*, generalized cross-validation; *CE*, classification error; RCV50, repeated 10-fold cross-validated deviance with *θ* = 0.5; RCV95, repeated 10-fold cross-validated deviance with *θ* = 0.95; AIC, Akaike’s information criterion; IP, shrinkage based on informative priors; WP, shrinkage based on weakly informative priors; FC, Firth’s correction. See Table S2 and S3 for results on scenarios with *N* = 1000. Results regarding RMSE of *β*_2_ are contained in Table S4 and S5

Figure 3 shows the RMSE of *β*_1_ across simulated scenarios and models with and without noise by means of nested loop plots [35, 36]. More detailed results also including scenarios with *N* = 1000 are contained in Table S2 and S3 of Additional file 1. As expected the best performance across all simulated scenarios was achieved by OEX. Generally, the performance of other tuned ridge regression approaches was extremely variable and unreliable and due to data sparsity the methods yielded coefficients with extremely large RMSE. Interestingly, the RMSE of *β*_1_ did not always decrease with increasing sample size and noise did not necessarily worsen the performance of those methods. In contrast, the methods where tuning was not required showed stable performance across all simulated scenarios. While the performance of Firth’s correction was satisfactory in almost all scenarios, suffering from RMSE larger than one in scenarios with the expected event rate E(*Y*) = 0.1 and sample size *N* = 100 only, it was clearly outperformed by IP that produced small RMSE of *β*_1_ across all scenarios. Although WP generally resulted in worse performance than Firth’s correction, it was less sensitive to very sparse data situations in which the performance of Firth’s correction was poor.

#### Accuracy of predictions

**Fig. 4:**
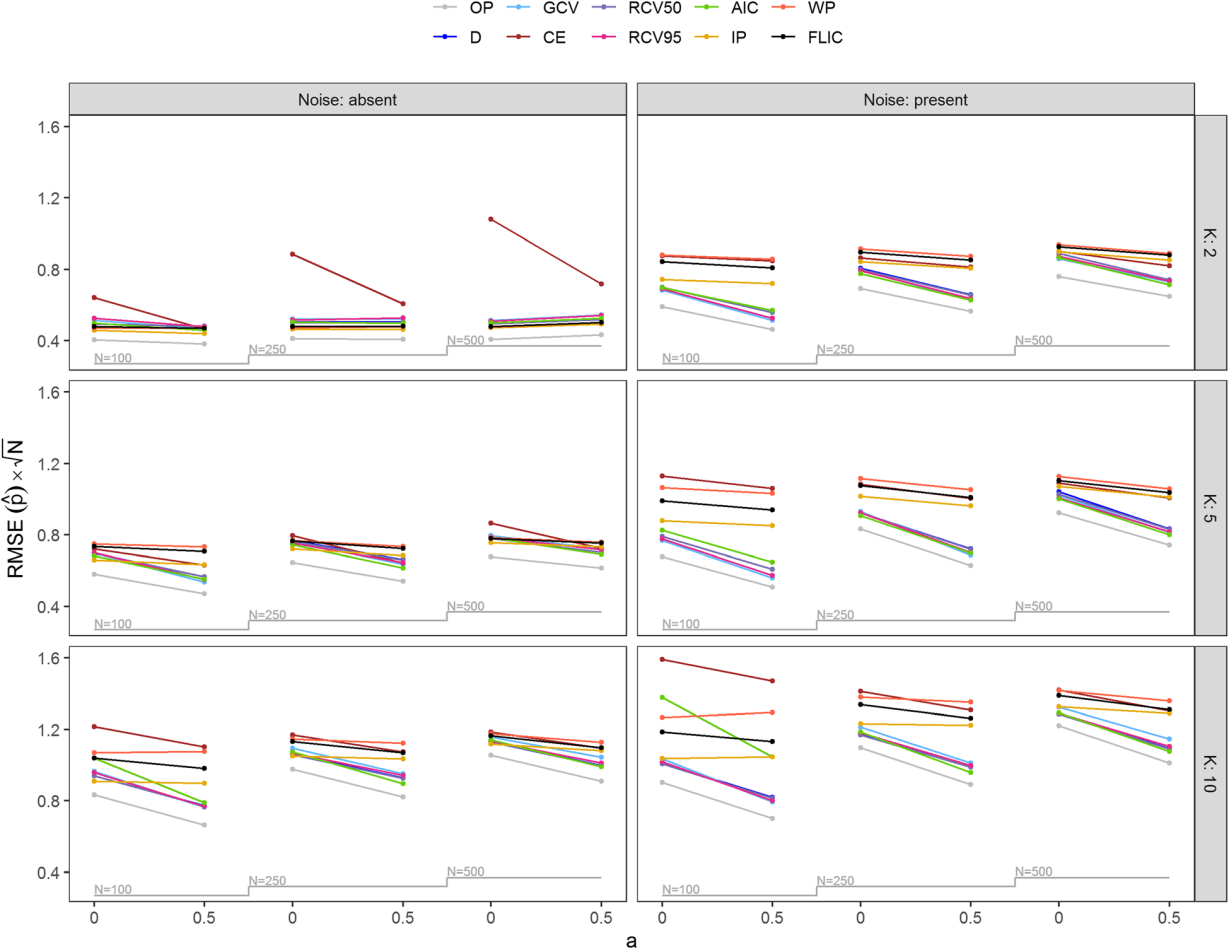
Nested loop plot of root mean squared error (RMSE) of predictions multiplied by the square root of sample size *N* by the number of predictors *K* ∈ {2, 5, 10}, noise absent or present, the sample size *N* ∈ {100, 250, 500} and the effect multiplier *a* ∈ {0.5, 1} for simulated scenarios with expected value of *Y*, E(*Y*) = 0.1. Further results are contained in Table S6 and S7. OP, prediction oracle; *D*, deviance; *GCV*, generalized cross-validation; *CE*, classification error; RCV50, repeated 10-fold cross-validated deviance with *θ* = 0.5; RCV95, repeated 10-fold cross-validated deviance with *θ* = 0.95; AIC, Akaike’s information criterion; IP, shrinkage based on informative priors; WP, shrinkage based on weakly informative priors; FLIC, Firth’s logistic regression with intercept-correction

Results regarding the RMSE of predicted probabilities for E(*Y*) = 0.1 are shown in Fig. 4. While the performance of the methods was similar in scenarios with larger sample sizes and no noise, the differences between them became apparent in scenarios with *N* = 100 and especially when including noise. Noise considerably worsened the performance of the methods. The least affected by noise were the methods based on cross-validation (apart from *CE*) that generally yielded the best performance. However, with no noise and strong effects (higher c-indices) IP always outperformed the other methods (apart from OP). The performance of FLIC and WP was consistently somewhat worse than the one of IP. The performance of AIC was similar to that of the cross-validation-based methods if there was no noise, however, when *N* = 100 it appeared sensitive to noise.

**Fig. 5:**
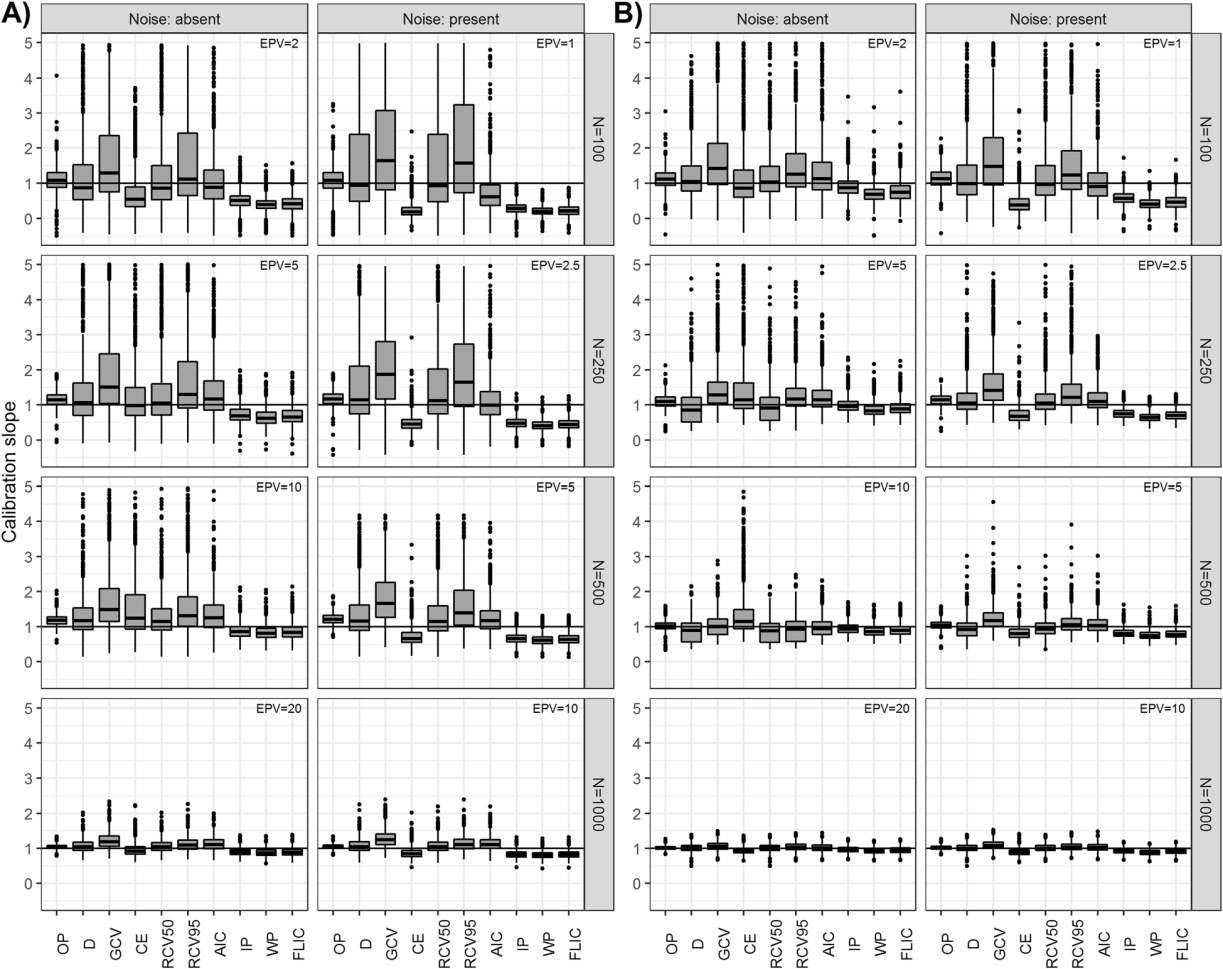
Boxplots showing distribution of calibration slopes over 1000 generated datasets in scenarios with the expected value of *Y*, E(*Y*) = 0.1, the number of predictors *K* = 5, noise absent or present, the sample size of *N* ∈ {100, 250, 500, 1000} considering A) moderate (*a* = 0.5) and B) strong (*a* = 1) predictors. Datasets where calibration slopes were larger than 5 are not shown. The whiskers extend no more than 1.5-times the interquartile range from the box. EPV, indicated in the top right, denotes the events per variable ratio. Further results from other scenarios are contained in Table S8 and S9. OP, prediction oracle; *D*, deviance; *GCV*, generalized cross-validation; *CE*, classification error; RCV50, repeated 10-fold cross-validated deviance with *θ* = 0.5; RCV95, repeated 10-fold cross-validated deviance with *θ* = 0.95; AIC, Akaike’s information criterion; IP, shrinkage based on informative priors; WP, shrinkage based on weakly informative priors; FLIC, Firth’s logistic regression with intercept-correction

Calibration slopes are presented by means of boxplots for scenarios with E(*Y*) = 0.1 and *K* = 5 (Fig. 5). For clarity of presentation, datasets where calibration slopes were larger than 5 are not shown. IP, WP and FLIC yielded similar performance with small variability over simulation runs and were generally close to the slope of 1 in strong effects scenarios but suffered from overfitting in moderate effects scenarios. The addition of noise increased overfitting of these methods. Tuning procedures (specifically, *D*, RCV50 and AIC) yielded calibration slopes that were in median relatively close to 1, however they suffered from large variability. While this variability decreased with *N*, there was still a considerable number of outliers produced by these methods even with *N* = 500, if the outcomes were very unbalanced and effects moderate only. Among tuning procedures AIC achieved on average the smallest variability of calibration slopes. With respect to the RMSD of the logarithm of calibration slopes (Table S10, S11 in Additional file 1) OP overall achieved the best performance, followed by IP if no noise was included or AIC in case of noise. Interestingly, noise did not necessarily increase the RMSD of tuning procedures and they appeared to be less sensitive to it as compared to the methods where shrinkage was pre-specified. Calibration slopes were strongly positively correlated with optimized *λ*^∗^ values (Fig. S1 in Additional file 1) and such were the correlations between the RMSD of calibration slopes and the variability of *λ*^∗^.

In terms of c-indices there was no considerable differences between methods (Table S12, S13 in Additional file 1).

## Data example

As an example we consider the study described by Poplas Susič et al. [37]. The aim of the study was to estimate the prevalence of dependence in daily activities (binary) and its risk factors in a group of individuals whose health status is not well known to family practice teams (patients who had not visited their chosen family physician in the last 5 years). Nine risk factors were considered: age (continuous), sex (binary), body mass index category (BMI; ordinal with 4 levels), APGAR (measuring family function via five constructs: Adaptation, Partnership, Growth, Affection, and Resolve; binary), chronic disease (CD; ordinal with four levels), fall (measuring increased risk of fall; binary), loneliness (measured on a discrete scale from 1 to 10; continuous), health (measured on a discrete scale from 1 to 10; continuous), pain (measured on a discrete scale from 1 to 10; continuous). Complete case analysis on a sample of individuals of size *N* = 1814, from which 423 (23%) had an event, was performed to quantify the effects of risk factors.

For our demonstration we randomly selected *N* = 275 individuals from the complete sample. According to recent guidance, the minimum required sample size for developing a prediction model was *N* = 273 based on an expected value of the (Cox-Snell) R-squared of 0.5, 9 predictors, expected value of events *E*(*Y*) = 0.23, and a desired level of shrinkage of 0.9 [9, 33]. In the subsample 60 (22%) events were observed. We used this subsample for fitting ridge regression and FLIC models while the remaining data served as a validation dataset for calculating the calibration slopes (Table 4). Ridge regression and FLIC models were fitted as described in Section 4.1.3. Most shrinkage was induced by tuned ridge regression methods with the exception of AIC and *CE* while methods with fixed penalization strength yielded calibration slopes (slightly) smaller than 1.

**Table 4:** Estimated regression coefficients for nine predictors of dependence in daily activities obtained from a subsample of size *N* = 275 by applying various versions of tuned ridge regression, ridge regression based on informative priors (IP), ridge regression based on weakly informative priors (WP) or Firth’s logistic regression with intercept-correction (FLIC). Tuning criteria: *D* deviance, *GCV*, generalized cross-validation, *CE*, classification error, RCV50, repeated 10-fold cross-validated deviance with *θ* = 0.5, RCV95, repeated 10-fold cross-validated deviance with *θ* = 0.95, AIC, Akaike’s information criterion. Calibration slopes were calculated on a validation dataset of size *N* = 1539

Method	Estimated coefficients										Calibration slope
	${\hat{\beta}}_0$	${\hat{\beta}}_{\mathrm{age}}$	${\hat{\beta}}_{\mathrm{sex}}$	${\hat{\beta}}_{\mathrm{BMI}}$	${\hat{\beta}}_{\mathrm{APGAR}}$	${\hat{\beta}}_{\mathrm{CD}}$	${\hat{\beta}}_{\mathrm{fall}}$	${\hat{\beta}}_{\mathrm{lonliness}}$	${\hat{\beta}}_{\mathrm{health}}$	${\hat{\beta}}_{\mathrm{pain}}$	
*D*	−4.79	0.05	−0.43	−0.25	1.25	0.57	2	0.04	−0.20	0.11	1.09
*GCV*	−4.53	0.05	−0.35	−0.22	1.14	0.54	1.90	0.05	−0.18	0.10	1.16
*CE*	−5.71	0.07	−0.72	−0.36	1.59	0.64	2.25	0.03	−0.25	0.13	0.89
RCV50	−4.85	0.06	−0.45	−0.26	1.27	0.57	2.02	0.04	−0.20	0.11	1.07
RCV95	−4.53	0.05	−0.35	−0.22	1.14	0.54	1.90	0.05	−0.18	0.10	1.16
AIC	−5.59	0.07	−0.69	−0.35	1.55	0.63	2.22	0.03	−0.24	0.13	0.91
IP	−5.33	0.07	−0.61	−0.32	1.46	0.61	2.16	0.03	−0.23	0.12	0.96
WP	−5.89	0.08	−0.78	−0.38	1.65	0.65	2.29	0.02	−0.26	0.14	0.87
FLIC	−5.84	0.07	−0.77	−0.37	1.61	0.60	2.16	0.02	−0.25	0.13	0.91

## Discussion

Numerous studies have shown that shrinkage is effective in preventing overfitting and may solve issues that arise in classical clinical settings with relatively large number of correlated covariates [2, 7, 8, 38]. Therefore, applying shrinkage has been recommended not only when developing prediction models but also when interest lies in coefficients with reduced MSE and inference is not required [7]. A recent study, however, noted that while calibration slopes obtained by shrinkage methods are on average close to 1 the variability of calibration slopes in small or sparse situations is large and therefore, improved performance in a single dataset cannot be guaranteed [11]. If considering that the number of observations (with event) constitutes the amount of information contained in the data, this may not seem surprising. However, many researchers would still utilize shrinkage methods in small or sparse datasets, expecting all problems to be solved. Therefore, in this paper, we have elaborated this issue further by focusing on ridge logistic regression. We evaluated its performance in a low-dimensional setting and compared it to Firth’s correction by means of simulation study. The amount of shrinkage in ridge regression was determined using different tuning procedures and prior assumptions, respectively. We were interested in the accuracy of both coefficient estimates and predictions.

With respect to large variability of calibration slopes, the results of our study confirm the findings of Van Calster et al. [11]. Furthermore, as already indicated by Riley et al. [13], we observed that the RMSD of the logarithm of calibration slopes was strongly correlated with the variability of optimized complexity parameters *λ*^∗^. By an illustrative example we demonstrated that tuning procedures might fail to approximate the U-shaped curve arising from the bias-variance trade-off and result in completely arbitrary choice of *λ*^∗^ that simply equals the smallest or the largest *λ* of the pre-specified sequence of values. However, in the simulation study we then observed that substantial variability of *λ*^∗^ must even be expected from the oracle that ‘knows’ the true event probabilities (prediction oracle) or regression coefficients (explanation oracle) and uses this knowledge to determine the optimal values of *λ*^∗^. This indicates that the variability of *λ*^∗^ by itself would not be that much of a problem but tuning procedures yielded optimized values that were negatively correlated with their ‘optimal counterparts’, determined by explanation or prediction oracle. On the one hand this can be explained by separation that often makes tuning procedures result in an optimized value of *λ*^∗^ close to zero [12]. In such datasets, if the amount of shrinkage is too small, this will yield coefficients with large MSE and predictions that may be numerically indistinguishable from zero or one by software packages [18]. While some may argue that this is not problematic when interest lies in predictions, we observed that the prediction oracle generally favored larger values of *λ*^∗^ than the explanation oracle, suggesting that in typical clinical studies seemingly perfect predictions should not be accepted as they potentially imply overfit and reflect increased variability of calibration slopes. On the other hand, as demonstrated in our illustration, if only a few observations prevent the data set from separation, a large value of *λ*^∗^ is needed to avoid very large out-of-sample prediction errors for the crucial, separation-preventing observations, while in fact the performance of maximum likelihood estimation in such datasets is already satisfactory [18]. In words of van Houwelingen [39], if *β* is ‘large’ by random fluctuation tuning procedures tend to keep the model large instead of correcting for the ‘large’ *β* by setting *λ*^∗^ > 0; and vice versa. In our simulation study we observed that for tuned ridge logistic regression calibration slopes were more stable in scenarios with larger sample sizes, more balanced outcomes, stronger effects and often even with a larger number of true predictors, while noise possibly increased the variability of calibration slopes. This suggests that simply breaking down the problem to a measure such as the events-per-variable ratio is unsatisfactory as not only the number of covariates but also their relations to the outcome are decisive here [8, 9].

Our results show that optimization of the complexity parameter in ridge regression is difficult in datasets where sampling variability is large and sampling artefacts, e.g. separation, are likely to occur. Van Calster et al. [11] instead suggested to apply FLIC [7] that provides only little shrinkage but results in lower variability of calibration slopes. Another convenient property of Firth’s correction, not shared by ridge regression, is its invariance to linear transformations of the design matrix. However, ridge regression may be generally preferred over Firth’s correction in the case of highly correlated covariates, where ***I***(**β**) is close to singularity, causing Firth’s correction to deteriorate. Alternatively, the choice of the complexity parameter in ridge regression may be based on prior expectations about the magnitude of the underlying effects [15]. Pre-specifying the degree of shrinkage seems reasonable as it stabilizes *λ*^∗^, and appeared beneficial in our study in which we included such a semi-Bayesian approach with zero-centered informative or weakly informative normal priors (IP and WP, respectively). Despite different motivation behind methods with fixed penalization strength, IP clearly outperformed tuned ridge regression (and Firth’s correction) with regard to RMSE of coefficients. With tuned ridge regression valid inference is hard to achieve due to bias introduced in the coefficients and additional variability that comes along with tuning *λ* (which possibly leads to less bias and more variance) [14, 40]. By contrast, for IP and WP (like with Firth’s correction) valid 95% posterior limits could be obtained easily by data augmentation, using any statistical software that enables maximum-likelihood fitting and weighting of observations [15]. Moreover, in scenarios with no noise included in the model IP yielded small RMSE of predictions and small RMSD of calibration slopes. Although one should usually devote additional work to specify prior distributions, we straightforwardly followed the outline of Sullivan and Greenland [15] in defining our priors, assuming that the true effects are not too extreme. IP therefore performed extremely well in all scenarios with strong effects, associated with higher true c-indices. While it seems that this approach is to some extent robust to misspecification of the prior, the results showed that with moderate effects only (and lower true c-indices) or with noise present in the data it could be reasonable to choose smaller prior variances (preferably for each coefficient separately) to better handle overfitting. However, if one is in doubt about how to determine the limits of the prior interval, weaker penalties are preferred. More guidance on how to specify prior distributions can be found in the paper by Greenland et al. [1].

Our study showed that particularly methods with a fixed degree of shrinkage were sensitive to noise, especially in terms of calibration slopes. For high-dimensional settings where much noise is contained in the data defining appropriate priors may be much more challenging and thus, these methods less appropriate. We expect that in such settings larger complexity parameter values will yield smaller out-of-sample prediction errors, thus tuning may be successful in preventing overfitting, but the problem of large calibration slopes due to very large optimized *λ*^∗^ might remain or even increase. Future research should investigate the behavior of tuning approaches in high-dimensional settings, also considering other penalized regression methods, e.g. lasso that in addition to shrinkage also performs variable selection. In a classical clinical prediction modelling context, however, lasso may be too restrictive and result in too sparse models. Furthermore, based on limited additional simulations (results not shown) we suspect that issues we discussed with respect to tuning will also appear with the lasso or any other tuned penalized regression method [11, 13].

Summarizing, while tuning has the potential to reduce the MSE of the estimates as demonstrated by the oracles, applying tuned ridge logistic regression in small or sparse datasets is problematic as tuned *λ*^∗^ values are highly variable and in addition negatively correlated with optimal values, yielding unstable coefficients and predictions. Naturally, only limited performance of the methods can be expected if little information is provided by the data, as is the case with small or sparse datasets. In order to alleviate the problem and allow for a more efficient use of available sample size, we recommend to determine the degree of shrinkage a priori with respect to some meaningful assumptions about true effects as demonstrated by Sullivan and Greenland [15]. Our simulations indicate that this approach has the potential to stabilize the estimates and may reduce bias of the coefficients away from zero such that relatively accurate coefficients and predictions can be obtained even in non-ideal settings which are typical e.g. in the context of rare outcomes or sparse predictors. Also with larger sample sizes an analysis might benefit from this approach, especially when (in addition to point estimates or predictions) valid Bayesian inference is required [1, 15, 16].

## Supplementary Information

**Additional file 1.**

## Data Availability

The code for illustration, simulation study and data example is contained in Additional file 1. The dependence in daily activities data analyzed in Section 5 are available from Antonija Poplas Susič (antonija.poplas-susic@zd-lj.si) but restrictions apply to the availability of these data, which were used under license for the current study, and so are not publicly available. The dependence in daily activities data cannot be shared by the authors due to violating confidentiality.

## Abbreviations

AIC: Akaike’s information criterium
*CE*: classification error
*D*: deviance
FLIC: Firth’s logistic regression with intercept-correction
*GCV*: generalized cross-validation
IP: ridge regression based on informative prior assumptions
MAD: median absolute deviation
MSE: mean squared error
OEX: explanation oracle
OP: prediction oracle
RCV: repeated 10-fold cross-validated deviance
RMSD: root mean squared distance
RMSE: root mean squared error
WP: ridge regression based on weakly informative prior assumptions
