# Supplementary material for: To tune or not to tune, a case study of ridge logistic regression in small or sparse datasets
Source: BMC Med Res Methodol. 2021 Sep 30;21:199. doi: 10.1186/s12874-021-01374-y (PMC8482588; doi:10.1186/s12874-021-01374-y)
Supplement: Supplementary file 1 — Additional file 1. [file 12874_2021_1374_MOESM1_ESM.pdf]

To tune or not to tune,
a case study of ridge logistic regression in small or sparse datasets

Hana Šinkovec, Georg Heinze, Rok Blagus, Angelika Geroldinger

Additional file 1

Minimum sample size required for developing a prediction model

Table S1: Minimum sample size required for developing a prediction model based on an expected value of the (Cox-Snell) R-squared, number of predictors, expected value of events $E(Y)$, and a desired level of shrinkage for different simulation scenarios that differed by the number of predictors $K \in \{2, 5, 10\}$, effect multiplier $a \in \{1, 0.5\}$ and noise absent or present. The level of shrinkage was set to 0.9 for scenarios with $a = 1$ and noise absent, 0.8 for scenarios with $a = 0.5$ and noise absent, 0.8 for scenarios with $a = 1$ and noise present, and 0.7 for scenarios with $a = 0.5$ and noise present.

		$E(Y) = 0.1$			$E(Y) = 0.25$		
			Required sample size			Required sample size	
a	K	R^2	Noise: absent	Noise: present	R^2	Noise: absent	Noise: present
0.5	2	0.05	155	315	0.08	289	289
	5	0.08	238	395	0.1	289	289
	10	0.15	380	569	0.15	289	399
1	2	0.15	139	266	0.2	289	289
	5	0.2	199	369	0.25	289	289
	10	0.35	334	501	0.35	289	348

Detailed simulation results

Table S2: Simulation results showing prevalence of separation (SP, %) and root mean squared errors of β_1 across simulation scenarios with marginal event rate $E(Y) = 0.10$ that differed by the number of predictors $K \in \{2, 5, 10\}$, sample size $N \in \{100, 250, 500, 1000\}$, effect multiplier $a \in \{1, 0.5\}$ and noise absent (0) or present (1). OEX, explanation oracle; D, deviance; GCV, generalized cross-validation; CE, classification error; RCV50, repeated 10-fold cross-validated deviance with $\theta = 0.5$; RCV95, repeated 10-fold cross-validated deviance with $\theta = 0.95$; AIC, Akaike's information criterion; IP, shrinkage based on informative priors; WP, shrinkage based on weakly informative priors; FC, Firth's correction.

K	N	β_1	a	Noise	SP	OEX	D	GCV	CE	RCV50	RCV95	AIC	IP	WP	FC
2	100	2.08	1	0	79	0.75	6.77	1.42	1.75	7.02	3.53	1.20	1.06	0.90	1.25
2	100	2.08	1	1	79	0.73	1.56	1.73	3.01	1.56	1.70	2.57	1.08	0.96	1.30
2	250	2.08	1	0	54	0.64	7.18	2.20	1.69	7.28	5.64	1.68	0.79	0.94	0.86
2	250	2.08	1	1	54	0.65	1.86	1.46	1.18	1.43	1.37	1.27	0.80	0.97	0.89
2	500	2.08	1	0	33	0.45	5.58	2.43	1.61	5.73	4.79	1.96	0.61	0.94	0.77
2	500	2.08	1	1	33	0.46	4.73	1.21	0.99	4.70	3.35	1.28	0.62	0.95	0.78
5	100	2.08	1	0	85	0.66	1.52	1.58	1.29	1.48	1.58	1.36	1.07	0.84	1.36
5	100	2.08	1	1	85	0.65	1.62	1.78	3.81	1.61	1.74	4.71	1.11	0.93	1.47
5	250	2.08	1	0	63	0.67	4.78	1.23	1.24	4.22	1.37	1.07	0.81	0.92	0.91
5	250	2.08	1	1	63	0.67	1.31	1.54	1.23	1.31	1.45	1.33	0.84	0.95	0.94
5	500	2.08	1	0	41	0.47	5.29	1.97	1.09	5.33	4.96	1.59	0.62	0.94	0.76
5	500	2.08	1	1	41	0.48	3.25	1.27	0.96	2.26	1.15	1.10	0.64	0.95	0.77
5	1000	2.08	1	0	18	0.39	3.56	2.03	0.97	3.59	3.51	1.69	0.56	0.88	0.74
5	1000	2.08	1	1	18	0.39	3.47	1.77	0.74	3.49	3.39	1.57	0.56	0.88	0.75
10	100	2.08	1	0	72	0.75	1.47	1.63	7.69	1.46	1.60	7.41	1.17	1.18	1.75
10	100	2.08	1	1	72	0.73	1.57	1.72	20.33	1.56	1.68	17.84	1.21	1.24	1.85
10	250	2.08	1	0	34	0.62	2.16	1.41	2.17	1.81	1.19	1.35	0.83	1.13	1.28
10	250	2.08	1	1	34	0.62	1.21	1.42	2.67	1.21	1.33	1.37	0.85	1.18	1.36
10	500	2.08	1	0	11	0.47	2.11	1.41	1.49	1.75	1.35	1.04	0.65	0.95	0.94
10	500	2.08	1	1	11	0.49	1.47	1.40	1.47	1.38	1.21	1.04	0.68	0.99	0.97
10	1000	2.08	1	0	1	0.36	1.06	0.79	0.71	1.06	0.82	0.75	0.54	0.67	0.65
10	1000	2.08	1	1	1	0.37	0.93	0.85	0.75	0.84	0.77	0.77	0.55	0.68	0.66
2	100	1.04	0.5	0	47	0.50	6.60	1.13	0.93	6.65	3.33	1.00	0.71	1.14	0.91
2	100	1.04	0.5	1	47	0.50	0.86	0.90	2.80	0.86	0.90	3.61	0.74	1.20	1.01
2	250	1.04	0.5	0	16	0.41	4.93	1.73	0.86	4.96	3.52	1.35	0.62	0.94	0.75
2	250	1.04	0.5	1	16	0.42	1.22	0.80	0.90	0.85	0.78	0.68	0.64	0.97	0.78
2	500	1.04	0.5	0	2	0.31	2.14	0.97	0.74	2.11	1.51	0.86	0.52	0.68	0.60
2	500	1.04	0.5	1	2	0.32	1.49	0.70	0.52	1.47	1.01	0.63	0.53	0.70	0.61
5	100	1.04	0.5	0	50	0.50	0.90	0.85	1.02	0.84	0.85	0.73	0.70	1.13	0.93
5	100	1.04	0.5	1	50	0.49	0.85	0.90	2.51	0.84	0.90	1.92	0.73	1.22	1.03
5	250	1.04	0.5	0	18	0.39	3.17	0.71	0.71	2.88	1.11	0.63	0.62	0.98	0.76
5	250	1.04	0.5	1	18	0.39	0.73	0.81	1.11	0.72	0.78	0.67	0.64	1.02	0.80
5	500	1.04	0.5	0	3	0.33	1.67	0.94	0.58	1.69	1.58	0.82	0.54	0.72	0.63
5	500	1.04	0.5	1	3	0.34	1.38	0.71	0.61	1.15	0.66	0.59	0.56	0.74	0.64
5	1000	1.04	0.5	0	0	0.26	0.52	0.51	0.46	0.52	0.53	0.47	0.43	0.49	0.46
5	1000	1.04	0.5	1	0	0.26	0.57	0.59	0.39	0.57	0.60	0.53	0.44	0.50	0.47
10	100	1.04	0.5	0	48	0.49	0.82	0.88	4.80	0.81	0.87	2.40	0.75	1.29	1.25
10	100	1.04	0.5	1	48	0.48	0.84	0.90	11.10	0.84	0.89	10.82	0.76	1.38	1.36
10	250	1.04	0.5	0	12	0.41	1.02	0.75	1.21	1.01	0.72	0.65	0.67	1.03	0.87
10	250	1.04	0.5	1	12	0.41	0.71	0.80	1.33	0.71	0.76	0.67	0.68	1.06	0.89
10	500	1.04	0.5	0	2	0.32	1.19	0.62	0.67	1.02	0.57	0.58	0.56	0.74	0.66
10	500	1.04	0.5	1	2	0.33	0.58	0.68	0.72	0.57	0.62	0.58	0.57	0.76	0.67
10	1000	1.04	0.5	0	0	0.26	0.43	0.50	0.42	0.42	0.45	0.45	0.42	0.46	0.44
10	1000	1.04	0.5	1	0	0.27	0.47	0.57	0.44	0.46	0.50	0.49	0.43	0.48	0.45

Table S3: Simulation results showing prevalence of separation (SP, %) and root mean squared errors of β_1 across simulation scenarios with marginal event rate $E(Y) = 0.25$ that differed by the number of predictors $K \in \{2, 5, 10\}$, sample size $N \in \{100, 250, 500, 1000\}$, effect multiplier $a \in \{1, 0.5\}$ and noise absent (0) or present (1). OEX, explanation oracle; D, deviance; GCV, generalized cross-validation; CE, classification error; RCV50, repeated 10-fold cross-validated deviance with $\theta = 0.5$; RCV95, repeated 10-fold cross-validated deviance with $\theta = 0.95$; AIC, Akaike's information criterion; IP, shrinkage based on informative priors; WP, shrinkage based on weakly informative priors; FC, Firth's correction.

K	N	β_1	a	Noise	SP	OEX	D	GCV	CE	RCV50	RCV95	AIC	IP	WP	FC
2	100	2.08	1	0	47	0.62	6.75	1.69	1.71	6.84	6.18	1.77	0.77	0.99	0.86
2	100	2.08	1	1	47	0.61	1.25	1.52	1.27	1.18	1.33	1.18	0.78	1.04	0.91
2	250	2.08	1	0	14	0.41	3.55	1.65	1.57	3.64	3.59	1.57	0.57	0.83	0.72
2	250	2.08	1	1	14	0.40	3.45	1.14	1.10	3.39	2.02	1.23	0.57	0.85	0.73
2	500	2.08	1	0	3	0.32	1.63	0.99	1.41	1.65	1.66	0.97	0.49	0.65	0.60
2	500	2.08	1	1	3	0.31	1.68	1.11	0.98	1.70	1.69	1.01	0.50	0.67	0.61
5	100	2.08	1	0	53	0.63	2.09	1.29	1.31	1.71	1.11	1.03	0.77	1.00	0.90
5	100	2.08	1	1	53	0.63	1.26	1.58	1.38	1.26	1.39	1.23	0.81	1.06	0.97
5	250	2.08	1	0	23	0.43	4.51	1.15	1.18	4.54	4.22	1.64	0.60	0.94	0.79
5	250	2.08	1	1	23	0.43	2.03	1.23	0.99	1.39	0.98	0.95	0.61	0.96	0.81
5	500	2.08	1	0	5	0.34	2.15	1.18	1.06	2.17	2.13	1.13	0.51	0.71	0.64
5	500	2.08	1	1	5	0.34	2.11	0.95	0.83	2.12	2.06	1.13	0.52	0.72	0.65
5	1000	2.08	1	0	0	0.26	0.58	0.54	0.91	0.59	0.60	0.50	0.38	0.44	0.42
5	1000	2.08	1	1	0	0.26	0.63	0.69	0.72	0.63	0.65	0.56	0.39	0.45	0.43
10	100	2.08	1	0	30	0.65	1.31	1.42	1.80	1.31	1.21	1.27	0.86	1.18	1.21
10	100	2.08	1	1	30	0.63	1.26	1.58	2.38	1.23	1.36	1.73	0.88	1.28	1.27
10	250	2.08	1	0	4	0.41	0.93	0.98	0.99	0.92	0.82	0.86	0.61	0.83	0.80
10	250	2.08	1	1	4	0.42	0.87	1.18	1.03	0.87	0.95	0.92	0.63	0.88	0.84
10	500	2.08	1	0	0	0.29	0.62	0.67	0.65	0.62	0.57	0.58	0.46	0.56	0.55
10	500	2.08	1	1	0	0.29	0.61	0.84	0.66	0.60	0.66	0.63	0.47	0.58	0.56
10	1000	2.08	1	0	0	0.23	0.38	0.45	0.44	0.38	0.40	0.39	0.35	0.39	0.38
10	1000	2.08	1	1	0	0.23	0.42	0.56	0.47	0.42	0.45	0.43	0.36	0.40	0.39
2	100	1.04	0.5	0	12	0.42	3.68	1.22	0.82	3.75	3.47	1.28	0.65	0.95	0.78
2	100	1.04	0.5	1	12	0.42	0.76	0.83	0.84	0.75	0.80	0.69	0.67	0.99	0.81
2	250	1.04	0.5	0	0	0.29	0.65	0.58	0.71	0.65	0.67	0.56	0.47	0.55	0.51
2	250	1.04	0.5	1	0	0.29	0.72	0.69	0.53	0.72	0.69	0.57	0.49	0.58	0.53
2	500	1.04	0.5	0	0	0.23	0.36	0.40	0.59	0.36	0.37	0.37	0.35	0.37	0.36
2	500	1.04	0.5	1	0	0.23	0.43	0.56	0.46	0.43	0.47	0.45	0.35	0.38	0.37
5	100	1.04	0.5	0	14	0.39	1.90	0.75	0.75	1.50	0.71	0.65	0.66	1.00	0.82
5	100	1.04	0.5	1	14	0.40	0.73	0.83	0.93	0.73	0.78	0.66	0.69	1.08	0.87
5	250	1.04	0.5	0	1	0.30	0.97	0.63	0.56	0.98	0.98	0.61	0.52	0.63	0.57
5	250	1.04	0.5	1	1	0.30	0.77	0.70	0.56	0.65	0.61	0.55	0.54	0.65	0.59
5	500	1.04	0.5	0	0	0.22	0.36	0.44	0.46	0.36	0.37	0.37	0.37	0.40	0.39
5	500	1.04	0.5	1	0	0.22	0.42	0.57	0.39	0.42	0.46	0.44	0.38	0.41	0.39
5	1000	1.04	0.5	0	0	0.18	0.26	0.33	0.36	0.26	0.28	0.28	0.26	0.27	0.26
5	1000	1.04	0.5	1	0	0.18	0.32	0.44	0.31	0.32	0.35	0.35	0.27	0.28	0.27
10	100	1.04	0.5	0	10	0.43	0.71	0.82	0.96	0.71	0.76	0.67	0.72	1.08	0.92
10	100	1.04	0.5	1	10	0.44	0.75	0.86	1.37	0.75	0.80	0.69	0.76	1.20	1.03
10	250	1.04	0.5	0	0	0.32	0.54	0.67	0.58	0.53	0.57	0.54	0.51	0.60	0.56
10	250	1.04	0.5	1	0	0.32	0.58	0.73	0.59	0.58	0.62	0.57	0.53	0.63	0.58
10	500	1.04	0.5	0	0	0.25	0.40	0.53	0.41	0.39	0.42	0.42	0.37	0.40	0.39
10	500	1.04	0.5	1	0	0.26	0.44	0.60	0.43	0.44	0.48	0.47	0.39	0.42	0.41
10	1000	1.04	0.5	0	0	0.17	0.27	0.36	0.29	0.27	0.28	0.29	0.26	0.27	0.26
10	1000	1.04	0.5	1	0	0.18	0.31	0.45	0.30	0.30	0.33	0.34	0.27	0.28	0.27

Table S4: Simulation results showing prevalence of separation (SP, %) and root mean squared errors of β_2 across simulation scenarios with marginal event rate $E(Y) = 0.10$ that differed by the number of predictors $K \in \{2, 5, 10\}$, sample size $N \in \{100, 250, 500, 1000\}$, effect multiplier $a \in \{1, 0.5\}$ and noise absent (0) or present (1). OEX, explanation oracle; D, deviance; GCV, generalized cross-validation; CE, classification error; RCV50, repeated 10-fold cross-validated deviance with $\theta = 0.5$; RCV95, repeated 10-fold cross-validated deviance with $\theta = 0.95$; AIC, Akaike's information criterion; IP, shrinkage based on informative priors; WP, shrinkage based on weakly informative priors; FC, Firth's correction.

K	N	β_2	a	Noise	SP	OEX	D	GCV	CE	RCV50	RCV95	AIC	IP	WP	FC
2	100	1.39	1	0	79	1.29	2.95	1.30	1.36	2.95	2.73	1.00	0.62	0.80	0.77
2	100	1.39	1	1	79	1.51	0.91	0.99	2.83	0.90	0.96	4.72	0.65	0.91	0.90
2	250	1.39	1	0	54	0.48	0.81	0.57	0.93	0.81	0.80	0.53	0.43	0.49	0.48
2	250	1.39	1	1	54	0.51	0.63	0.69	0.55	0.56	0.63	0.56	0.44	0.52	0.49
2	500	1.39	1	0	33	0.33	0.33	0.34	0.81	0.33	0.33	0.33	0.32	0.34	0.33
2	500	1.39	1	1	33	0.34	0.37	0.45	0.42	0.36	0.39	0.38	0.32	0.34	0.34
5	100	1.39	1	0	85	1.43	1.25	0.90	0.95	1.02	0.88	0.84	0.63	0.82	0.77
5	100	1.39	1	1	85	1.78	0.93	1.04	3.87	0.92	0.99	5.24	0.65	0.93	0.87
5	250	1.39	1	0	63	0.50	0.49	0.56	0.60	0.49	0.52	0.50	0.45	0.51	0.49
5	250	1.39	1	1	63	0.53	0.60	0.75	0.56	0.60	0.68	0.59	0.47	0.55	0.51
5	500	1.39	1	0	41	0.33	0.33	0.35	0.45	0.33	0.33	0.33	0.31	0.34	0.33
5	500	1.39	1	1	41	0.35	0.37	0.50	0.36	0.37	0.42	0.41	0.33	0.35	0.34
5	1000	1.39	1	0	18	0.24	0.24	0.25	0.35	0.24	0.24	0.24	0.23	0.24	0.24
5	1000	1.39	1	1	18	0.25	0.26	0.32	0.26	0.26	0.28	0.28	0.24	0.25	0.24
10	100	1.39	1	0	72	2.50	0.95	1.01	8.74	0.92	0.92	7.59	0.69	0.99	1.04
10	100	1.39	1	1	72	3.37	0.96	1.04	13.35	0.92	0.97	10.66	0.71	1.09	0.99
10	250	1.39	1	0	34	0.58	0.53	0.60	0.65	0.53	0.56	0.55	0.47	0.59	0.55
10	250	1.39	1	1	34	0.64	0.58	0.69	0.75	0.57	0.63	0.59	0.49	0.64	0.58
10	500	1.39	1	0	11	0.39	0.36	0.40	0.41	0.36	0.37	0.37	0.35	0.39	0.38
10	500	1.39	1	1	11	0.40	0.39	0.47	0.42	0.39	0.42	0.40	0.36	0.41	0.38
10	1000	1.39	1	0	1	0.27	0.25	0.27	0.27	0.25	0.26	0.26	0.25	0.27	0.26
10	1000	1.39	1	1	1	0.27	0.26	0.30	0.28	0.26	0.28	0.27	0.26	0.27	0.26
2	100	0.69	0.5	0	47	0.62	1.66	0.86	0.62	1.67	1.59	0.71	0.60	0.75	0.71
2	100	0.69	0.5	1	47	2.64	0.62	0.60	2.44	0.62	0.60	4.89	0.63	0.85	0.80
2	250	0.69	0.5	0	16	0.43	0.44	0.44	0.52	0.44	0.45	0.43	0.43	0.47	0.46
2	250	0.69	0.5	1	16	0.45	0.45	0.48	0.46	0.45	0.48	0.42	0.45	0.50	0.48
2	500	0.69	0.5	0	2	0.30	0.30	0.31	0.43	0.30	0.31	0.30	0.31	0.32	0.31
2	500	0.69	0.5	1	2	0.31	0.34	0.38	0.32	0.34	0.36	0.33	0.31	0.33	0.32
5	100	0.69	0.5	0	50	0.61	0.58	0.57	0.73	0.58	0.58	0.56	0.60	0.76	0.71
5	100	0.69	0.5	1	50	0.73	0.59	0.59	3.47	0.59	0.59	5.56	0.63	0.88	0.79
5	250	0.69	0.5	0	18	0.40	0.43	0.45	0.41	0.42	0.44	0.39	0.41	0.44	0.43
5	250	0.69	0.5	1	18	0.42	0.45	0.50	0.44	0.45	0.48	0.41	0.42	0.47	0.44
5	500	0.69	0.5	0	3	0.31	0.32	0.35	0.34	0.32	0.34	0.31	0.32	0.33	0.32
5	500	0.69	0.5	1	3	0.33	0.36	0.41	0.33	0.36	0.39	0.35	0.33	0.34	0.33
5	1000	0.69	0.5	0	0	0.22	0.22	0.24	0.26	0.22	0.23	0.22	0.22	0.22	0.22
5	1000	0.69	0.5	1	0	0.23	0.25	0.31	0.23	0.25	0.28	0.26	0.22	0.23	0.23
10	100	0.69	0.5	0	48	3.56	0.65	0.64	5.08	0.62	0.58	4.00	0.66	0.94	0.85
10	100	0.69	0.5	1	48	3.23	0.59	0.59	9.37	0.59	0.58	8.93	0.68	1.06	0.94
10	250	0.69	0.5	0	12	0.42	0.41	0.45	0.46	0.41	0.43	0.39	0.42	0.47	0.45
10	250	0.69	0.5	1	12	0.45	0.43	0.48	0.52	0.43	0.46	0.40	0.44	0.52	0.47
10	500	0.69	0.5	0	2	0.31	0.30	0.34	0.34	0.30	0.32	0.30	0.32	0.34	0.32
10	500	0.69	0.5	1	2	0.33	0.32	0.38	0.35	0.32	0.34	0.32	0.33	0.36	0.34
10	1000	0.69	0.5	0	0	0.23	0.22	0.25	0.25	0.22	0.23	0.23	0.23	0.24	0.23
10	1000	0.69	0.5	1	0	0.23	0.24	0.28	0.25	0.23	0.25	0.24	0.24	0.24	0.24

Table S5: Simulation results showing prevalence of separation (SP, %) and root mean squared errors of β_2 across simulation scenarios with marginal event rate $E(Y) = 0.25$ that differed by the number of predictors $K \in \{2, 5, 10\}$, sample size $N \in \{100, 250, 500, 1000\}$, effect multiplier $a \in \{1, 0.5\}$ and noise absent (0) or present (1). OEX, explanation oracle; D, deviance; GCV, generalized cross-validation; CE, classification error; RCV50, repeated 10-fold cross-validated deviance with $\theta = 0.5$; RCV95, repeated 10-fold cross-validated deviance with $\theta = 0.95$; AIC, Akaike's information criterion; IP, shrinkage based on informative priors; WP, shrinkage based on weakly informative priors; FC, Firth's correction.

K	N	β_2	a	Noise	SP	OEX	D	GCV	CE	RCV50	RCV95	AIC	IP	WP	FC
2	100	1.39	1	0	47	0.51	0.52	0.54	1.02	0.52	0.52	0.52	0.47	0.52	0.51
2	100	1.39	1	1	47	0.57	0.60	0.79	0.63	0.59	0.67	0.59	0.49	0.58	0.54
2	250	1.39	1	0	14	0.33	0.32	0.34	0.83	0.32	0.33	0.33	0.32	0.33	0.33
2	250	1.39	1	1	14	0.34	0.37	0.50	0.52	0.37	0.40	0.39	0.33	0.35	0.34
2	500	1.39	1	0	3	0.22	0.22	0.23	0.66	0.22	0.22	0.22	0.22	0.22	0.22
2	500	1.39	1	1	3	0.23	0.24	0.31	0.41	0.24	0.25	0.25	0.23	0.23	0.23
5	100	1.39	1	0	53	0.52	0.52	0.66	0.69	0.52	0.57	0.54	0.46	0.53	0.51
5	100	1.39	1	1	53	0.59	0.65	0.87	0.64	0.65	0.73	0.63	0.49	0.62	0.57
5	250	1.39	1	0	23	0.33	0.33	0.38	0.58	0.33	0.34	0.33	0.31	0.33	0.33
5	250	1.39	1	1	23	0.36	0.38	0.57	0.43	0.38	0.43	0.41	0.33	0.36	0.34
5	500	1.39	1	0	5	0.24	0.24	0.26	0.47	0.24	0.24	0.24	0.24	0.24	0.24
5	500	1.39	1	1	5	0.25	0.26	0.37	0.34	0.26	0.28	0.28	0.24	0.25	0.25
5	1000	1.39	1	0	0	0.17	0.16	0.17	0.35	0.16	0.16	0.16	0.16	0.16	0.16
5	1000	1.39	1	1	0	0.17	0.17	0.21	0.26	0.17	0.18	0.18	0.17	0.17	0.17
10	100	1.39	1	0	30	0.69	0.59	0.75	0.72	0.58	0.63	0.64	0.52	0.68	0.63
10	100	1.39	1	1	30	0.81	0.65	0.87	1.14	0.65	0.71	1.09	0.55	0.80	0.72
10	250	1.39	1	0	4	0.38	0.36	0.45	0.43	0.36	0.38	0.37	0.35	0.39	0.37
10	250	1.39	1	1	4	0.41	0.40	0.55	0.44	0.40	0.43	0.41	0.36	0.42	0.39
10	500	1.39	1	0	0	0.26	0.25	0.28	0.30	0.25	0.25	0.25	0.25	0.26	0.25
10	500	1.39	1	1	0	0.27	0.27	0.35	0.30	0.27	0.28	0.28	0.26	0.27	0.26
10	1000	1.39	1	0	0	0.18	0.17	0.19	0.20	0.17	0.18	0.18	0.17	0.18	0.18
10	1000	1.39	1	1	0	0.19	0.18	0.22	0.21	0.18	0.19	0.19	0.18	0.19	0.18
2	100	0.69	0.5	0	12	0.46	0.46	0.48	0.53	0.47	0.48	0.45	0.46	0.51	0.50
2	100	0.69	0.5	1	12	0.48	0.49	0.53	0.47	0.49	0.51	0.45	0.50	0.56	0.53
2	250	0.69	0.5	0	0	0.29	0.30	0.33	0.43	0.30	0.31	0.30	0.30	0.31	0.31
2	250	0.69	0.5	1	0	0.31	0.35	0.41	0.32	0.35	0.37	0.34	0.32	0.33	0.32
2	500	0.69	0.5	0	0	0.22	0.21	0.23	0.32	0.21	0.22	0.21	0.22	0.22	0.22
2	500	0.69	0.5	1	0	0.22	0.24	0.30	0.26	0.24	0.26	0.25	0.23	0.23	0.23
5	100	0.69	0.5	0	14	0.46	0.46	0.49	0.46	0.46	0.47	0.43	0.47	0.53	0.51
5	100	0.69	0.5	1	14	0.51	0.48	0.53	0.52	0.48	0.51	0.44	0.51	0.60	0.55
5	250	0.69	0.5	0	1	0.29	0.30	0.36	0.34	0.30	0.32	0.30	0.30	0.31	0.31
5	250	0.69	0.5	1	1	0.31	0.34	0.42	0.31	0.34	0.37	0.33	0.31	0.33	0.31
5	500	0.69	0.5	0	0	0.22	0.21	0.25	0.27	0.21	0.22	0.22	0.22	0.22	0.22
5	500	0.69	0.5	1	0	0.23	0.25	0.33	0.24	0.25	0.27	0.26	0.23	0.23	0.23
5	1000	0.69	0.5	0	0	0.16	0.15	0.18	0.20	0.15	0.16	0.16	0.15	0.15	0.15
5	1000	0.69	0.5	1	0	0.16	0.17	0.23	0.18	0.17	0.19	0.19	0.16	0.16	0.16
10	100	0.69	0.5	0	10	0.51	0.46	0.51	0.52	0.46	0.48	0.44	0.51	0.61	0.55
10	100	0.69	0.5	1	10	0.56	0.47	0.54	0.57	0.47	0.50	0.44	0.55	0.70	0.61
10	250	0.69	0.5	0	0	0.31	0.31	0.38	0.32	0.31	0.33	0.31	0.32	0.34	0.32
10	250	0.69	0.5	1	0	0.33	0.33	0.42	0.33	0.33	0.35	0.32	0.33	0.36	0.33
10	500	0.69	0.5	0	0	0.23	0.22	0.28	0.24	0.22	0.23	0.23	0.23	0.23	0.23
10	500	0.69	0.5	1	0	0.24	0.24	0.32	0.24	0.24	0.25	0.25	0.24	0.24	0.24
10	1000	0.69	0.5	0	0	0.17	0.15	0.18	0.17	0.15	0.16	0.16	0.16	0.16	0.16
10	1000	0.69	0.5	1	0	0.17	0.16	0.22	0.17	0.16	0.17	0.17	0.17	0.17	0.17

Table S6: Simulation results showing root mean squared errors of predictions ($\times 10000$) across simulation scenarios with marginal event rate $E(Y) = 0.10$ that differed by the number of predictors $K \in \{2, 5, 10\}$, sample size $N \in \{100, 250, 500, 1000\}$, effect multiplier $a \in \{1, 0.5\}$ and noise absent (0) or present (1). OP, prediction oracle; D, deviance; GCV, generalized cross-validation; CE, classification error; RCV50, repeated 10-fold cross-validated deviance with $\theta = 0.5$; RCV95, repeated 10-fold cross-validated deviance with $\theta = 0.95$; AIC, Akaike's information criterion; IP, shrinkage based on informative priors; WP, shrinkage based on weakly informative priors; FLIC, Firth's logistic regression with intercept-correction.

K	N	a	Noise	OP	D	GCV	CE	RCV50	RCV95	AIC	IP	WP	FLIC
2	100	1	0	405	493	514	641	493	525	498	459	469	480
2	100	1	1	591	700	684	877	701	691	697	744	881	843
2	250	1	0	261	319	330	560	317	327	319	295	299	303
2	250	1	1	439	510	504	547	506	501	491	533	579	568
2	500	1	0	182	223	230	483	222	228	223	211	214	215
2	500	1	1	340	398	384	404	398	391	388	402	420	415
5	100	1	0	580	697	698	721	699	701	682	660	749	736
5	100	1	1	678	793	769	1131	793	778	827	881	1066	990
5	250	1	0	409	486	484	503	483	477	471	456	487	484
5	250	1	1	527	585	589	686	584	584	574	643	707	681
5	500	1	0	304	349	356	387	349	352	351	338	351	349
5	500	1	1	413	466	456	487	460	451	449	480	504	494
5	1000	1	0	229	256	260	304	256	257	258	253	258	257
5	1000	1	1	320	350	355	357	350	351	351	355	364	361
10	100	1	0	833	940	966	1216	938	958	1039	909	1068	1039
10	100	1	1	902	1011	1036	1591	1006	1018	1377	1037	1267	1185
10	250	1	0	617	672	692	739	669	675	678	664	725	716
10	250	1	1	693	741	767	893	739	746	749	779	873	846
10	500	1	0	471	506	518	530	505	507	510	499	525	521
10	500	1	1	545	575	592	635	575	577	578	594	633	621
10	1000	1	0	347	367	374	377	367	370	371	365	376	374
10	1000	1	1	411	430	439	452	430	431	433	438	453	449
2	100	0.5	0	381	483	468	467	483	480	459	440	472	470
2	100	0.5	1	463	559	514	848	559	526	570	720	857	810
2	250	0.5	0	258	319	331	384	318	334	315	293	304	304
2	250	0.5	1	357	417	398	515	415	404	398	510	553	540
2	500	0.5	0	194	232	244	321	232	242	235	220	225	225
2	500	0.5	1	291	331	326	367	332	329	319	382	398	394
5	100	0.5	0	472	566	538	632	567	550	553	634	733	708
5	100	0.5	1	508	608	559	1061	608	575	648	853	1033	939
5	250	0.5	0	343	418	402	407	417	408	389	434	465	458
5	250	0.5	1	398	456	435	636	456	444	443	609	667	639
5	500	0.5	0	275	314	323	324	315	321	310	328	340	337
5	500	0.5	1	332	373	364	450	372	366	358	453	474	465
5	1000	0.5	0	204	230	239	246	231	233	232	237	242	241
5	1000	0.5	1	264	290	291	320	290	289	285	329	337	334
10	100	0.5	0	664	773	762	1102	771	768	789	898	1077	980
10	100	0.5	1	701	818	792	1470	814	801	1045	1047	1294	1132
10	250	0.5	0	519	587	602	679	586	596	566	654	710	676
10	250	0.5	1	564	625	639	827	624	631	605	773	855	798
10	500	0.5	0	406	446	467	489	445	452	443	483	504	490
10	500	0.5	1	453	489	512	583	488	494	482	577	608	586
10	1000	0.5	0	315	340	357	360	340	343	343	356	364	359
10	1000	0.5	1	358	381	401	421	381	384	381	423	434	427

Table S7: Simulation results showing root mean squared errors of predictions ($\times 10000$) across simulation scenarios with marginal event rate $E(Y) = 0.25$ that differed by the number of predictors $K \in \{2, 5, 10\}$, sample size $N \in \{100, 250, 500, 1000\}$, effect multiplier $a \in \{1, 0.5\}$ and noise absent (0) or present (1). OP, prediction oracle; D, deviance; GCV, generalized cross-validation; CE, classification error; RCV50, repeated 10-fold cross-validated deviance with $\theta = 0.5$; RCV95, repeated 10-fold cross-validated deviance with $\theta = 0.95$; AIC, Akaike's information criterion; IP, shrinkage based on informative priors; WP, shrinkage based on weakly informative priors; FLIC, Firth's logistic regression with intercept-correction.

K	N	a	Noise	OP	D	GCV	CE	RCV50	RCV95	AIC	IP	WP	FLIC
2	100	1	0	586	700	749	1289	696	720	698	645	654	663
2	100	1	1	921	1031	1109	1086	1029	1050	1016	1053	1153	1124
2	250	1	0	375	438	467	1045	435	451	439	416	419	422
2	250	1	1	642	710	758	787	709	711	711	711	740	730
2	500	1	0	270	307	323	829	306	316	309	298	299	300
2	500	1	1	485	524	554	622	524	527	529	528	540	536
5	100	1	0	834	946	1026	1076	943	960	944	900	966	961
5	100	1	1	1040	1141	1229	1229	1139	1157	1133	1211	1350	1298
5	250	1	0	562	621	659	841	620	629	627	603	624	623
5	250	1	1	750	814	872	854	811	810	810	837	879	861
5	500	1	0	417	451	475	688	451	456	456	445	453	452
5	500	1	1	576	611	648	649	611	613	615	620	635	628
5	1000	1	0	295	314	323	517	314	316	315	312	315	315
5	1000	1	1	416	434	452	492	435	435	437	438	444	441
10	100	1	0	1109	1187	1305	1292	1186	1207	1209	1178	1303	1287
10	100	1	1	1246	1324	1455	1524	1322	1339	1371	1390	1583	1530
10	250	1	0	783	825	891	882	824	834	834	817	857	851
10	250	1	1	915	954	1039	1021	954	961	963	979	1042	1021
10	500	1	0	569	595	627	640	595	600	599	591	607	604
10	500	1	1	681	705	752	738	705	709	709	714	737	729
10	1000	1	0	408	423	437	441	423	425	425	421	427	426
10	1000	1	1	498	512	533	534	512	514	515	516	525	521
2	100	0.5	0	591	734	773	842	732	765	719	670	699	698
2	100	0.5	1	784	896	881	1006	897	888	870	1070	1170	1120
2	250	0.5	0	386	463	513	659	461	486	470	435	444	443
2	250	0.5	1	584	656	676	682	656	660	640	737	766	750
2	500	0.5	0	277	324	357	505	323	335	332	314	318	318
2	500	0.5	1	446	490	521	506	489	495	487	530	540	534
5	100	0.5	0	748	885	883	886	881	878	844	931	1011	988
5	100	0.5	1	852	962	935	1197	963	945	951	1254	1395	1312
5	250	0.5	0	541	617	659	652	617	630	612	641	664	657
5	250	0.5	1	662	729	742	810	728	727	711	869	908	881
5	500	0.5	0	396	438	478	500	438	444	442	453	462	459
5	500	0.5	1	511	551	581	579	551	554	546	618	632	622
5	1000	0.5	0	294	319	348	373	319	323	324	324	326	326
5	1000	0.5	1	396	422	447	433	421	424	423	448	453	449
10	100	0.5	0	1003	1108	1151	1241	1108	1119	1082	1265	1399	1324
10	100	0.5	1	1078	1178	1204	1454	1178	1180	1170	1490	1685	1554
10	250	0.5	0	722	786	870	829	785	801	781	842	879	858
10	250	0.5	1	813	869	943	962	868	880	857	1012	1065	1023
10	500	0.5	0	565	600	670	627	600	608	606	625	638	631
10	500	0.5	1	649	681	753	723	681	688	682	749	768	753
10	1000	0.5	0	412	434	477	456	433	437	440	444	449	446
10	1000	0.5	1	486	506	559	525	506	509	511	535	542	536

Table S8: Simulation results showing median calibration slopes (with 5th and 95th percentile) across simulation scenarios with marginal event rate $E(Y) = 0.1$ that differed by the number of predictors $K \in \{2, 5, 10\}$, sample size $N \in \{100, 250, 500, 1000\}$, effect multiplier $a \in \{1, 0.5\}$ and noise absent (0) or present (1). OP, prediction oracle; D, deviance; GCV, generalized cross-validation; CE, classification error; RCV50, repeated 10-fold cross-validated deviance with $\theta = 0.5$; RCV95, repeated 10-fold cross-validated deviance with $\theta = 0.95$; AIC, Akaike's information criterion; IP, shrinkage based on informative priors; WP, shrinkage based on weakly informative priors; FLIC, Firth's logistic regression with intercept-correction.

K	N	a	Noise	OP	D	GCV	CE	RCV50	RCV95	AIC	IP	WP	FLIC
2	100	1	0	1.1 (0.3, 2.1)	0.7 (0.2, 5.7)	1.4 (0.5, 15.3)	4.9 (0.8, 11.5)	0.5 (0.2, 4.8)	1.3 (0.2, 28.1)	1.2 (0.4, 5.1)	1.2 (0.7, 2.8)	1 (0.5, 2.1)	1.1 (0.5, 2.6)
2	100	1	1	1.2 (0.7, 1.7)	1.2 (0.4, 11.5)	1.8 (0.5, 12.1)	0.5 (0.2, 1.7)	1.1 (0.4, 11.3)	1.6 (0.5, 12.7)	1 (0.3, 2.9)	0.7 (0.4, 1.1)	0.5 (0.2, 0.8)	0.5 (0.2, 1)
2	250	1	0	1.1 (0.3, 1.6)	0.4 (0.2, 2.1)	0.8 (0.6, 2.5)	4.3 (1, 8.7)	0.4 (0.2, 2.1)	0.8 (0.3, 2.4)	0.9 (0.6, 2.2)	1.1 (0.7, 1.8)	1 (0.6, 1.6)	1 (0.7, 1.7)
2	250	1	1	1.1 (0.9, 1.4)	1 (0.5, 2.3)	1.4 (0.8, 3.6)	0.8 (0.5, 2)	1 (0.6, 2.3)	1.2 (0.7, 3.3)	1.1 (0.7, 2.2)	0.8 (0.6, 1.1)	0.7 (0.5, 1)	0.7 (0.5, 1.1)
2	500	1	0	1 (0.3, 1.3)	1 (0.2, 1.5)	1 (0.5, 1.6)	2.8 (1.1, 4.3)	0.9 (0.2, 1.5)	1 (0.4, 1.5)	0.9 (0.6, 1.5)	1 (0.7, 1.4)	0.9 (0.7, 1.3)	0.9 (0.7, 1.3)
2	500	1	1	1.1 (0.9, 1.2)	0.9 (0.3, 1.4)	1.1 (0.7, 1.8)	1 (0.6, 1.9)	0.9 (0.3, 1.4)	1 (0.3, 1.6)	1 (0.6, 1.5)	0.8 (0.6, 1.1)	0.8 (0.6, 1)	0.8 (0.6, 1.1)
5	100	1	0	1.1 (0.6, 1.6)	1.1 (0.4, 15.4)	1.6 (0.5, 17.1)	0.9 (0.3, 4)	1.1 (0.4, 14.9)	1.4 (0.5, 17.4)	1.1 (0.4, 3.5)	0.9 (0.5, 1.5)	0.7 (0.3, 1.2)	0.7 (0.3, 1.4)
5	100	1	1	1.1 (0.7, 1.6)	1.1 (0.4, 8.8)	1.8 (0.6, 9.1)	0.4 (0.1, 1)	1.1 (0.4, 8.7)	1.5 (0.5, 9.1)	0.9 (0.3, 2.2)	0.6 (0.3, 0.9)	0.4 (0.2, 0.7)	0.5 (0.1, 0.8)
5	250	1	0	1.1 (0.8, 1.4)	0.9 (0.4, 2.1)	1.3 (0.8, 3)	1.2 (0.7, 4.2)	0.9 (0.4, 2.1)	1.2 (0.7, 2.7)	1.1 (0.7, 2.3)	1 (0.7, 1.5)	0.8 (0.6, 1.3)	0.9 (0.6, 1.4)
5	250	1	1	1.1 (0.9, 1.4)	1.1 (0.7, 2.4)	1.4 (0.8, 3.9)	0.7 (0.4, 1.2)	1.1 (0.7, 2.4)	1.2 (0.7, 3.5)	1.1 (0.7, 2)	0.7 (0.5, 1)	0.6 (0.4, 0.9)	0.7 (0.5, 1)
5	500	1	0	1 (0.8, 1.2)	0.9 (0.5, 1.4)	1 (0.6, 1.6)	1.1 (0.8, 2.8)	0.9 (0.5, 1.4)	0.9 (0.5, 1.5)	0.9 (0.7, 1.5)	0.9 (0.7, 1.2)	0.9 (0.7, 1.2)	0.9 (0.7, 1.2)
5	500	1	1	1 (0.9, 1.2)	0.9 (0.5, 1.4)	1.2 (0.8, 1.8)	0.8 (0.6, 1.2)	0.9 (0.5, 1.4)	1 (0.7, 1.6)	1 (0.7, 1.5)	0.8 (0.6, 1)	0.7 (0.6, 1)	0.8 (0.6, 1)
5	1000	1	0	1.1 (0.9, 1.3)	1 (0.6, 1.4)	1.1 (0.7, 1.4)	1.2 (0.9, 2.2)	1 (0.6, 1.3)	1.1 (0.6, 1.4)	1 (0.8, 1.4)	1 (0.8, 1.3)	1 (0.8, 1.2)	1 (0.8, 1.3)
5	1000	1	1	1.1 (1, 1.2)	1 (0.6, 1.3)	1.1 (0.7, 1.6)	1 (0.8, 1.3)	1 (0.6, 1.3)	1.1 (0.6, 1.4)	1.1 (0.7, 1.4)	0.9 (0.8, 1.2)	0.9 (0.7, 1.1)	0.9 (0.8, 1.2)
10	100	1	0	1 (0.6, 1.5)	1.1 (0.5, 4.3)	1.4 (0.5, 8)	0.4 (0, 1.2)	1.1 (0.5, 4.7)	1.3 (0.6, 9.1)	0.9 (0.1, 2)	0.8 (0.5, 1.1)	0.5 (0.3, 0.9)	0.6 (0.3, 1)
10	100	1	1	1 (0.6, 1.6)	1.1 (0.4, 4.8)	1.5 (0.6, 7.1)	0.2 (0, 1)	1 (0.4, 4.9)	1.3 (0.6, 7.6)	0.8 (0, 1.8)	0.6 (0.4, 0.9)	0.4 (0.2, 0.6)	0.5 (0.2, 0.8)
10	250	1	0	1 (0.8, 1.2)	1 (0.6, 1.5)	1.2 (0.8, 1.8)	0.7 (0.5, 1.2)	1 (0.7, 1.5)	1.1 (0.7, 1.7)	1 (0.6, 1.5)	0.9 (0.7, 1.1)	0.7 (0.5, 1)	0.8 (0.5, 1.1)
10	250	1	1	1 (0.8, 1.2)	1 (0.7, 1.5)	1.2 (0.8, 2)	0.6 (0.4, 1)	1 (0.7, 1.5)	1.1 (0.7, 1.8)	1 (0.6, 1.5)	0.8 (0.6, 1)	0.6 (0.4, 0.8)	0.7 (0.5, 0.9)
10	500	1	0	1 (0.8, 1.1)	1 (0.7, 1.3)	1.1 (0.8, 1.4)	0.8 (0.6, 1.1)	1 (0.7, 1.3)	1 (0.8, 1.3)	1 (0.7, 1.3)	0.9 (0.7, 1.1)	0.8 (0.7, 1)	0.9 (0.7, 1.1)
10	500	1	1	1 (0.9, 1.1)	1 (0.8, 1.3)	1.1 (0.8, 1.5)	0.8 (0.6, 1)	1 (0.8, 1.3)	1 (0.8, 1.4)	1 (0.7, 1.3)	0.8 (0.7, 1)	0.8 (0.6, 1)	0.8 (0.6, 1)
10	1000	1	0	1 (0.9, 1.1)	1 (0.8, 1.2)	1.1 (0.9, 1.2)	0.9 (0.8, 1.1)	1 (0.8, 1.2)	1 (0.9, 1.2)	1 (0.8, 1.2)	1 (0.8, 1.1)	0.9 (0.8, 1.1)	0.9 (0.8, 1.1)
10	1000	1	1	1 (0.9, 1.1)	1 (0.8, 1.2)	1.1 (0.9, 1.3)	0.9 (0.8, 1.1)	1 (0.8, 1.2)	1 (0.9, 1.2)	1 (0.9, 1.2)	0.9 (0.8, 1.1)	0.9 (0.8, 1)	0.9 (0.8, 1)
2	100	0.5	0	1.2 (-0.4, 2.2)	0.8 (0, 27.2)	1.9 (-0.1, 27.2)	3.6 (-0.2, 22.2)	0.8 (0, 27.2)	2.3 (0, 27.2)	1.3 (-0.1, 18.1)	0.9 (0, 2.5)	0.6 (0, 2)	0.8 (-0.2, 2.3)
2	100	0.5	1	1.2 (-0.9, 1.8)	1.2 (-0.4, 7.8)	2 (-0.4, 7.8)	0.3 (0, 0.9)	1.2 (-0.4, 7.8)	2.3 (-0.6, 7.8)	0.7 (-0.2, 2.8)	0.4 (0, 0.7)	0.3 (0, 0.5)	0.3 (0, 0.6)
2	250	0.5	0	1.1 (0.7, 1.9)	1.2 (0.1, 10.7)	1.5 (0.3, 10.7)	4.1 (0.8, 8)	1.2 (0.1, 10.7)	1.4 (0.1, 10.7)	1.3 (0.3, 6.5)	0.9 (0.5, 2)	0.8 (0.4, 1.9)	0.9 (0.5, 2.1)
2	250	0.5	1	1.1 (0.8, 1.4)	1.1 (0.4, 4.3)	1.8 (0.6, 4.4)	0.5 (0.2, 1.5)	1.1 (0.4, 4.3)	1.6 (0.5, 4.4)	1 (0.4, 2.9)	0.5 (0.2, 0.9)	0.4 (0.2, 0.8)	0.5 (0.2, 0.8)
2	500	0.5	0	1.1 (0.8, 1.6)	1.1 (0.6, 2.9)	1.3 (0.7, 4)	2.8 (1.1, 5)	1.1 (0.6, 2.8)	1.2 (0.7, 4.2)	1.1 (0.6, 2.9)	0.9 (0.6, 1.7)	0.9 (0.6, 1.6)	0.9 (0.6, 1.7)
2	500	0.5	1	1.1 (0.8, 1.3)	1.1 (0.5, 2.8)	1.5 (0.7, 3.1)	0.7 (0.4, 1.8)	1 (0.5, 2.8)	1.3 (0.6, 3.1)	1.1 (0.6, 2.3)	0.6 (0.4, 1)	0.6 (0.4, 0.9)	0.6 (0.4, 1)
5	100	0.5	0	1.1 (0.3, 1.7)	1.2 (0.1, 10.6)	2 (0.1, 10.7)	0.5 (0.1, 2.3)	1.1 (0.1, 10.6)	2.3 (0.2, 10.7)	0.9 (0.1, 4)	0.5 (0.1, 1)	0.4 (0, 0.8)	0.4 (0, 0.9)
5	100	0.5	1	1.1 (0.1, 1.7)	1 (0.1, 5.6)	1.9 (0.1, 5.7)	0.2 (0, 0.6)	1 (0.1, 5.6)	1.9 (0.1, 5.8)	0.6 (0, 1.9)	0.3 (0, 0.5)	0.2 (0, 0.4)	0.2 (0, 0.5)
5	250	0.5	0	1.1 (0.8, 1.5)	1.1 (0.1, 6.3)	1.7 (0.7, 6.5)	1 (0.4, 3.3)	1.1 (0.1, 6.3)	1.5 (0.6, 6.5)	1.2 (0.5, 3.7)	0.7 (0.4, 1.2)	0.6 (0.3, 1.1)	0.6 (0.4, 1.2)
5	250	0.5	1	1.2 (0.8, 1.5)	1.1 (0.4, 4)	1.9 (0.6, 4.1)	0.5 (0.2, 0.9)	1.1 (0.4, 4)	1.7 (0.5, 4.1)	1 (0.4, 2.4)	0.5 (0.3, 0.7)	0.4 (0.2, 0.7)	0.4 (0.2, 0.7)
5	500	0.5	0	1.2 (0.9, 1.5)	1.2 (0.7, 2.8)	1.5 (0.8, 3.9)	1.2 (0.7, 3.3)	1.1 (0.6, 2.7)	1.3 (0.7, 3.9)	1.2 (0.7, 2.5)	0.9 (0.6, 1.3)	0.8 (0.5, 1.2)	0.8 (0.6, 1.3)
5	500	0.5	1	1.2 (1, 1.5)	1.2 (0.6, 2.9)	1.7 (0.9, 3)	0.7 (0.4, 1.3)	1.1 (0.6, 2.9)	1.4 (0.7, 3)	1.2 (0.7, 2.3)	0.7 (0.5, 1)	0.6 (0.4, 0.9)	0.6 (0.4, 0.9)
5	1000	0.5	0	1.1 (1, 1.4)	1.1 (0.8, 1.8)	1.3 (0.8, 2.3)	1.3 (0.8, 2.4)	1.1 (0.8, 1.8)	1.2 (0.8, 2.1)	1.2 (0.8, 2)	0.9 (0.7, 1.3)	0.9 (0.7, 1.3)	0.9 (0.7, 1.3)
5	1000	0.5	1	1.2 (1, 1.4)	1.1 (0.7, 1.9)	1.4 (0.9, 2.3)	0.9 (0.6, 1.4)	1.1 (0.7, 1.9)	1.2 (0.8, 2.1)	1.2 (0.8, 1.9)	0.8 (0.6, 1.1)	0.8 (0.6, 1)	0.8 (0.6, 1.1)
10	100	0.5	0	1 (0.3, 1.9)	1.1 (0.2, 7.3)	1.8 (0.3, 7.3)	0.3 (0, 1.1)	1.1 (0.2, 7.3)	1.8 (0.3, 7.5)	0.8 (0.1, 2.1)	0.5 (0, 0.7)	0.3 (0, 0.6)	0.4 (0, 0.7)
10	100	0.5	1	1 (0.3, 2)	1.1 (0.1, 5.8)	1.9 (0.3, 5.9)	0.2 (0, 0.7)	1.1 (0.2, 5.8)	1.8 (0.3, 5.9)	0.7 (0, 1.7)	0.4 (0, 0.6)	0.2 (0, 0.4)	0.3 (0, 0.5)
10	250	0.5	0	1 (0.8, 1.3)	1.1 (0.6, 3.6)	1.5 (0.8, 4.1)	0.6 (0.3, 1.3)	1.1 (0.6, 3.6)	1.3 (0.7, 4.1)	1.1 (0.6, 2.1)	0.6 (0.4, 0.9)	0.6 (0.3, 0.8)	0.6 (0.4, 0.9)
10	250	0.5	1	1 (0.8, 1.3)	1.1 (0.6, 3.4)	1.6 (0.8, 3.6)	0.5 (0.2, 1)	1.1 (0.6, 3.4)	1.3 (0.7, 3.6)	1 (0.6, 1.9)	0.5 (0.3, 0.8)	0.5 (0.3, 0.7)	0.5 (0.3, 0.7)
10	500	0.5	0	1 (0.9, 1.2)	1 (0.7, 1.6)	1.3 (0.8, 2.2)	0.8 (0.6, 1.3)	1 (0.7, 1.6)	1.1 (0.8, 1.9)	1.1 (0.7, 1.6)	0.8 (0.6, 1)	0.7 (0.5, 1)	0.8 (0.6, 1)
10	500	0.5	1	1 (0.9, 1.2)	1 (0.7, 1.7)	1.3 (0.9, 2.3)	0.7 (0.5, 1.1)	1 (0.7, 1.7)	1.1 (0.8, 2)	1.1 (0.7, 1.6)	0.7 (0.5, 0.9)	0.6 (0.5, 0.9)	0.7 (0.5, 0.9)
10	1000	0.5	0	1 (0.9, 1.2)	1 (0.8, 1.4)	1.2 (0.9, 1.6)	0.9 (0.7, 1.4)	1 (0.8, 1.4)	1.1 (0.9, 1.5)	1.1 (0.9, 1.5)	0.9 (0.7, 1.1)	0.9 (0.7, 1.1)	0.9 (0.7, 1.1)
10	1000	0.5	1	1 (0.9, 1.2)	1 (0.8, 1.4)	1.2 (0.9, 1.7)	0.8 (0.7, 1.2)	1 (0.8, 1.4)	1.1 (0.9, 1.5)	1.1 (0.9, 1.5)	0.8 (0.7, 1)	0.8 (0.6, 1)	0.8 (0.7, 1)

Table S9: Simulation results showing median calibration slopes (with 5th and 95th percentile) across simulation scenarios with marginal event rate $E(Y) = 0.25$ that differed by the number of predictors $K \in \{2, 5, 10\}$, sample size $N \in \{100, 250, 500, 1000\}$, effect multiplier $a \in \{1, 0.5\}$ and noise absent (0) or present (1). OP, prediction oracle; D, deviance; GCV, generalized cross-validation; CE, classification error; RCV50, repeated 10-fold cross-validated deviance with $\theta = 0.5$; RCV95, repeated 10-fold cross-validated deviance with $\theta = 0.95$; AIC, Akaike's information criterion; IP, shrinkage based on informative priors; WP, shrinkage based on weakly informative priors; FLIC, Firth's logistic regression with intercept-correction.

K	N	a	Noise	OP	D	GCV	CE	RCV50	RCV95	AIC	IP	WP	FLIC
2	100	1	0	1.1 (0.2, 1.6)	0.9 (0.2, 2.3)	1.1 (0.6, 3.1)	5.5 (1, 9.7)	0.9 (0.2, 2.2)	0.9 (0.2, 2.6)	0.9 (0.6, 2.3)	1.1 (0.8, 1.8)	0.9 (0.7, 1.6)	1 (0.7, 1.7)
2	100	1	1	1.1 (0.9, 1.4)	1.1 (0.6, 2.7)	1.8 (0.9, 5.4)	1.1 (0.5, 3.1)	1.1 (0.6, 2.6)	1.3 (0.7, 4.2)	1.1 (0.6, 2.2)	0.8 (0.5, 1.1)	0.6 (0.4, 1)	0.7 (0.4, 1.1)
2	250	1	0	1.1 (0.9, 1.4)	1.1 (0.3, 1.6)	1.2 (0.5, 1.8)	3.1 (1.2, 4.5)	1.1 (0.3, 1.6)	1.1 (0.3, 1.7)	1.1 (0.6, 1.6)	1.1 (0.8, 1.5)	1 (0.7, 1.4)	1 (0.8, 1.4)
2	250	1	1	1.1 (0.9, 1.3)	1 (0.3, 1.6)	1.3 (0.9, 2.4)	1.3 (0.8, 2.8)	1 (0.3, 1.6)	1.1 (0.7, 1.8)	1.1 (0.6, 1.7)	0.9 (0.7, 1.2)	0.8 (0.6, 1.2)	0.9 (0.7, 1.2)
2	500	1	0	1 (0.9, 1.2)	1 (0.8, 1.3)	1.1 (0.8, 1.4)	2.1 (1.4, 2.6)	1 (0.8, 1.3)	1.1 (0.8, 1.4)	1 (0.8, 1.3)	1 (0.8, 1.3)	1 (0.8, 1.2)	1 (0.8, 1.2)
2	500	1	1	1 (0.9, 1.2)	1 (0.8, 1.3)	1.2 (0.8, 1.6)	1.2 (0.8, 2.1)	1 (0.8, 1.3)	1 (0.8, 1.4)	1 (0.8, 1.4)	0.9 (0.7, 1.1)	0.9 (0.7, 1.1)	0.9 (0.7, 1.1)
5	100	1	0	1 (0.8, 1.3)	1 (0.5, 2.1)	1.5 (0.8, 3.6)	1.3 (0.6, 5.2)	1 (0.6, 2.1)	1.2 (0.7, 2.6)	1.1 (0.6, 2.1)	0.9 (0.6, 1.3)	0.7 (0.5, 1.1)	0.8 (0.6, 1.2)
5	100	1	1	1.1 (0.9, 1.3)	1.1 (0.6, 2.4)	1.8 (0.9, 5)	0.8 (0.4, 2)	1 (0.6, 2.3)	1.3 (0.7, 3.5)	1 (0.5, 1.9)	0.7 (0.5, 1)	0.5 (0.4, 0.8)	0.6 (0.4, 0.9)
5	250	1	0	1 (0.9, 1.2)	1 (0.3, 1.4)	1.2 (0.7, 1.8)	1.4 (0.8, 3.5)	1 (0.3, 1.4)	1 (0.3, 1.5)	1 (0.6, 1.5)	0.9 (0.7, 1.2)	0.9 (0.7, 1.2)	0.9 (0.7, 1.2)
5	250	1	1	1 (0.9, 1.2)	1 (0.6, 1.5)	1.4 (0.9, 2.2)	1 (0.6, 2)	1 (0.7, 1.5)	1.1 (0.8, 1.6)	1 (0.7, 1.5)	0.8 (0.6, 1.1)	0.7 (0.6, 1)	0.8 (0.6, 1.1)
5	500	1	0	1 (0.9, 1.2)	1 (0.7, 1.3)	1.1 (0.8, 1.5)	1.4 (0.9, 2.4)	1 (0.7, 1.3)	1 (0.7, 1.3)	1 (0.7, 1.3)	1 (0.8, 1.2)	0.9 (0.7, 1.2)	0.9 (0.8, 1.2)
5	500	1	1	1 (0.9, 1.1)	1 (0.7, 1.3)	1.2 (0.9, 1.6)	1 (0.8, 1.7)	1 (0.7, 1.3)	1 (0.7, 1.3)	1 (0.7, 1.3)	0.9 (0.7, 1.1)	0.9 (0.7, 1.1)	0.9 (0.7, 1.1)
5	1000	1	0	1.1 (1, 1.2)	1.1 (0.9, 1.2)	1.1 (0.9, 1.3)	1.4 (1, 1.9)	1 (0.9, 1.2)	1.1 (0.9, 1.3)	1 (0.9, 1.2)	1 (0.9, 1.2)	1 (0.9, 1.2)	1 (0.9, 1.2)
5	1000	1	1	1.1 (1, 1.2)	1 (0.9, 1.2)	1.1 (0.9, 1.4)	1.1 (0.9, 1.6)	1 (0.9, 1.2)	1.1 (0.9, 1.2)	1.1 (0.9, 1.2)	1 (0.9, 1.1)	1 (0.8, 1.1)	1 (0.8, 1.1)
10	100	1	0	1 (0.8, 1.2)	1 (0.6, 1.8)	1.5 (0.8, 3.4)	0.9 (0.4, 2.3)	1 (0.6, 1.8)	1.2 (0.7, 2.2)	1 (0.5, 1.7)	0.8 (0.6, 1.1)	0.6 (0.4, 0.9)	0.7 (0.4, 1)
10	100	1	1	1 (0.8, 1.3)	1 (0.6, 1.9)	1.7 (0.9, 4)	0.7 (0.3, 2)	1 (0.6, 1.9)	1.2 (0.7, 2.4)	0.9 (0.4, 1.6)	0.7 (0.5, 0.9)	0.5 (0.3, 0.7)	0.6 (0.3, 0.9)
10	250	1	0	1 (0.9, 1.2)	1 (0.7, 1.4)	1.2 (0.9, 1.8)	1 (0.7, 1.7)	1 (0.7, 1.4)	1.1 (0.8, 1.5)	1 (0.7, 1.4)	0.9 (0.7, 1.1)	0.8 (0.6, 1.1)	0.9 (0.7, 1.1)
10	250	1	1	1 (0.9, 1.2)	1 (0.7, 1.4)	1.3 (0.9, 2)	0.9 (0.6, 1.6)	1 (0.7, 1.4)	1.1 (0.8, 1.5)	1 (0.7, 1.4)	0.8 (0.7, 1)	0.7 (0.6, 0.9)	0.8 (0.6, 1)
10	500	1	0	1 (0.9, 1.1)	1 (0.9, 1.2)	1.1 (0.9, 1.4)	1 (0.8, 1.6)	1 (0.8, 1.2)	1.1 (0.9, 1.3)	1 (0.8, 1.3)	1 (0.8, 1.1)	0.9 (0.8, 1.1)	0.9 (0.8, 1.1)
10	500	1	1	1 (0.9, 1.1)	1 (0.8, 1.2)	1.2 (0.9, 1.5)	1 (0.8, 1.4)	1 (0.8, 1.2)	1.1 (0.9, 1.3)	1 (0.8, 1.3)	0.9 (0.8, 1.1)	0.9 (0.7, 1)	0.9 (0.8, 1.1)
10	1000	1	0	1 (0.9, 1.1)	1 (0.9, 1.2)	1.1 (0.9, 1.2)	1 (0.9, 1.3)	1 (0.9, 1.2)	1 (0.9, 1.2)	1 (0.9, 1.2)	1 (0.9, 1.1)	1 (0.8, 1.1)	1 (0.8, 1.1)
10	1000	1	1	1 (0.9, 1.1)	1 (0.9, 1.1)	1.1 (0.9, 1.3)	1 (0.8, 1.3)	1 (0.9, 1.1)	1 (0.9, 1.2)	1 (0.9, 1.2)	1 (0.8, 1.1)	0.9 (0.8, 1)	0.9 (0.8, 1.1)
2	100	0.5	0	1.2 (0.9, 1.9)	1.4 (0.1, 13.6)	2.1 (0.3, 13.6)	4.4 (0.9, 9.5)	1.3 (0.1, 13.6)	1.7 (0.1, 13.6)	1.4 (0.3, 8.7)	1 (0.6, 2.2)	0.9 (0.4, 2)	0.9 (0.5, 2.2)
2	100	0.5	1	1.2 (0.8, 1.5)	1.3 (0.4, 5.4)	2.5 (0.6, 5.4)	0.6 (0.2, 2)	1.3 (0.4, 5.4)	2 (0.5, 5.4)	1.1 (0.4, 3)	0.5 (0.2, 0.9)	0.4 (0.2, 0.8)	0.5 (0.2, 0.8)
2	250	0.5	0	1.1 (0.9, 1.7)	1.2 (0.7, 2.9)	1.5 (0.8, 4.6)	2.9 (1.2, 5)	1.2 (0.7, 2.8)	1.4 (0.7, 4)	1.3 (0.7, 2.9)	1 (0.7, 1.7)	1 (0.6, 1.7)	1 (0.6, 1.7)
2	250	0.5	1	1.1 (1, 1.4)	1.2 (0.6, 3.1)	1.9 (0.9, 3.4)	1 (0.5, 2.3)	1.2 (0.6, 3.2)	1.4 (0.7, 3.4)	1.2 (0.7, 2.7)	0.7 (0.5, 1.1)	0.7 (0.4, 1)	0.7 (0.5, 1.1)
2	500	0.5	0	1.1 (1, 1.5)	1.1 (0.8, 1.8)	1.3 (0.9, 2.3)	2 (1.4, 2.9)	1.1 (0.8, 1.8)	1.2 (0.8, 2)	1.2 (0.8, 1.9)	1 (0.7, 1.5)	1 (0.7, 1.5)	1 (0.7, 1.5)
2	500	0.5	1	1.1 (1, 1.3)	1.1 (0.8, 1.9)	1.5 (1, 2.4)	1.2 (0.7, 2)	1.1 (0.8, 1.9)	1.2 (0.8, 2.2)	1.2 (0.8, 1.9)	0.8 (0.6, 1.1)	0.8 (0.6, 1.1)	0.8 (0.6, 1.2)
5	100	0.5	0	1 (0.8, 1.4)	1.1 (0.3, 6.3)	2 (0.6, 6.4)	0.9 (0.4, 3.3)	1 (0.4, 6.3)	1.5 (0.5, 6.4)	1 (0.4, 3.2)	0.6 (0.3, 1)	0.5 (0.3, 0.9)	0.6 (0.3, 1)
5	100	0.5	1	1 (0.8, 1.4)	1.1 (0.4, 3.9)	2.1 (0.7, 4)	0.5 (0.2, 1.4)	1 (0.4, 3.9)	1.5 (0.5, 4)	0.8 (0.4, 2)	0.4 (0.2, 0.6)	0.3 (0.1, 0.6)	0.4 (0.1, 0.6)
5	250	0.5	0	1 (0.9, 1.2)	1 (0.6, 2.4)	1.5 (0.8, 3.5)	1.2 (0.6, 2.9)	1 (0.6, 2.4)	1.2 (0.6, 3.4)	1.1 (0.6, 2.3)	0.8 (0.5, 1.2)	0.7 (0.5, 1.1)	0.8 (0.5, 1.2)
5	250	0.5	1	1 (0.9, 1.2)	1 (0.6, 2.4)	1.7 (0.9, 2.6)	0.7 (0.4, 1.6)	1 (0.6, 2.4)	1.2 (0.6, 2.6)	1 (0.6, 1.9)	0.6 (0.4, 0.8)	0.6 (0.4, 0.8)	0.6 (0.4, 0.8)
5	500	0.5	0	1.1 (0.9, 1.2)	1.1 (0.8, 1.6)	1.3 (0.9, 2.3)	1.4 (0.8, 2.3)	1 (0.8, 1.6)	1.1 (0.8, 1.8)	1.1 (0.8, 1.7)	0.9 (0.7, 1.2)	0.9 (0.7, 1.2)	0.9 (0.7, 1.2)
5	500	0.5	1	1.1 (1, 1.2)	1 (0.7, 1.7)	1.5 (1, 2.1)	0.9 (0.6, 1.6)	1 (0.8, 1.7)	1.1 (0.8, 1.9)	1.1 (0.8, 1.7)	0.8 (0.6, 1)	0.8 (0.6, 1)	0.8 (0.6, 1)
5	1000	0.5	0	1.1 (1, 1.2)	1.1 (0.9, 1.4)	1.2 (0.9, 1.7)	1.3 (0.9, 1.8)	1.1 (0.8, 1.4)	1.1 (0.9, 1.5)	1.1 (0.9, 1.5)	1 (0.8, 1.2)	1 (0.8, 1.2)	1 (0.8, 1.2)
5	1000	0.5	1	1.1 (1, 1.2)	1.1 (0.8, 1.4)	1.3 (1, 1.7)	1.1 (0.8, 1.5)	1.1 (0.8, 1.4)	1.1 (0.9, 1.5)	1.1 (0.9, 1.5)	0.9 (0.7, 1.1)	0.9 (0.7, 1.1)	0.9 (0.7, 1.1)
10	100	0.5	0	1 (0.7, 1.3)	1 (0.5, 4.4)	2 (0.8, 4.7)	0.6 (0.3, 1.9)	1 (0.5, 4.3)	1.4 (0.6, 4.7)	0.9 (0.4, 2.1)	0.5 (0.3, 0.8)	0.4 (0.2, 0.7)	0.5 (0.2, 0.8)
10	100	0.5	1	1 (0.7, 1.3)	1 (0.5, 3.6)	2.1 (0.8, 3.7)	0.4 (0.2, 1.4)	1 (0.4, 3.5)	1.4 (0.5, 3.7)	0.8 (0.4, 1.7)	0.4 (0.2, 0.6)	0.3 (0.1, 0.5)	0.3 (0.2, 0.6)
10	250	0.5	0	1.1 (0.9, 1.2)	1.1 (0.7, 2)	1.7 (1, 3.1)	0.9 (0.6, 2)	1.1 (0.7, 2)	1.3 (0.8, 2.5)	1.2 (0.7, 2)	0.8 (0.6, 1.1)	0.7 (0.5, 1)	0.8 (0.6, 1.1)
10	250	0.5	1	1.1 (0.9, 1.2)	1.1 (0.7, 2.1)	1.8 (1, 2.7)	0.8 (0.5, 1.8)	1.1 (0.7, 2.1)	1.3 (0.8, 2.5)	1.1 (0.7, 1.8)	0.7 (0.5, 0.9)	0.6 (0.4, 0.8)	0.7 (0.5, 0.9)
10	500	0.5	0	1 (0.9, 1.2)	1 (0.8, 1.5)	1.3 (1, 2)	1 (0.7, 1.6)	1 (0.8, 1.5)	1.1 (0.8, 1.6)	1.1 (0.8, 1.5)	0.9 (0.7, 1.1)	0.8 (0.7, 1.1)	0.9 (0.7, 1.1)
10	500	0.5	1	1 (0.9, 1.2)	1 (0.8, 1.5)	1.4 (1, 2)	0.9 (0.7, 1.5)	1 (0.8, 1.5)	1.1 (0.8, 1.6)	1.1 (0.8, 1.5)	0.8 (0.6, 1)	0.8 (0.6, 0.9)	0.8 (0.6, 1)
10	1000	0.5	0	1 (0.9, 1.1)	1 (0.8, 1.2)	1.1 (0.9, 1.5)	1 (0.8, 1.4)	1 (0.8, 1.2)	1 (0.8, 1.3)	1 (0.8, 1.3)	0.9 (0.8, 1)	0.9 (0.7, 1)	0.9 (0.8, 1.1)
10	1000	0.5	1	1 (0.9, 1.1)	1 (0.8, 1.2)	1.2 (1, 1.5)	0.9 (0.7, 1.3)	1 (0.8, 1.2)	1 (0.8, 1.3)	1 (0.8, 1.3)	0.8 (0.7, 1)	0.8 (0.7, 1)	0.8 (0.7, 1)

Table S10: Simulation results showing root mean squared distance of the logarithm of calibration slopes across simulation scenarios with marginal event rate $E(Y) = 0.1$ that differed by the number of predictors $K \in \{2, 5, 10\}$, sample size $N \in \{100, 250, 500, 1000\}$, effect multiplier $a \in \{1, 0.5\}$ and noise absent (0) or present (1). OP, prediction oracle; D, deviance; GCV, generalized cross-validation; CE, classification error; RCV50, repeated 10-fold cross-validated deviance with $\theta = 0.5$; RCV95, repeated 10-fold cross-validated deviance with $\theta = 0.95$; AIC, Akaike's information criterion; IP, shrinkage based on informative priors; WP, shrinkage based on weakly informative priors; FLIC, Firth's logistic regression with intercept-correction.

K	N	a	Noise	OP	D	GCV	CE	RCV50	RCV95	AIC	IP	WP	FLIC
2	100	1	0	0.75	1.20	1.16	1.70	1.19	1.38	0.87	0.60	0.52	0.59
2	100	1	1	0.39	1.08	1.24	1.02	1.07	1.28	0.77	0.66	0.91	0.94
2	250	1	0	0.50	0.89	0.48	1.42	0.89	0.77	0.40	0.29	0.28	0.28
2	250	1	1	0.23	0.49	0.62	0.47	0.46	0.56	0.38	0.33	0.45	0.39
2	500	1	0	0.36	0.67	0.37	1.00	0.68	0.59	0.32	0.19	0.22	0.20
2	500	1	1	0.16	0.63	0.31	0.35	0.63	0.48	0.28	0.26	0.33	0.29
5	100	1	0	0.40	1.07	1.22	0.78	1.06	1.28	0.71	0.46	0.62	0.62
5	100	1	1	0.38	0.98	1.15	1.37	0.98	1.16	0.87	0.76	1.08	1.11
5	250	1	0	0.28	0.57	0.53	0.61	0.52	0.47	0.38	0.22	0.28	0.25
5	250	1	1	0.20	0.40	0.62	0.49	0.40	0.53	0.34	0.36	0.50	0.43
5	500	1	0	0.20	0.43	0.30	0.43	0.43	0.41	0.26	0.18	0.22	0.20
5	500	1	1	0.13	0.36	0.31	0.31	0.29	0.24	0.22	0.26	0.33	0.29
5	1000	1	0	0.15	0.23	0.22	0.36	0.23	0.24	0.19	0.13	0.14	0.14
5	1000	1	1	0.13	0.25	0.26	0.18	0.25	0.27	0.21	0.14	0.17	0.15
10	100	1	0	0.41	0.76	0.91	1.42	0.76	0.94	1.07	0.48	0.84	0.78
10	100	1	1	0.40	0.76	0.93	2.20	0.75	0.93	1.87	0.62	1.06	1.02
10	250	1	0	0.14	0.27	0.32	0.42	0.26	0.27	0.28	0.23	0.37	0.33
10	250	1	1	0.14	0.26	0.36	0.59	0.25	0.29	0.28	0.33	0.52	0.44
10	500	1	0	0.09	0.20	0.19	0.25	0.18	0.17	0.18	0.16	0.23	0.20
10	500	1	1	0.09	0.18	0.22	0.32	0.18	0.17	0.18	0.22	0.31	0.26
10	1000	1	0	0.06	0.11	0.12	0.13	0.11	0.11	0.11	0.10	0.12	0.11
10	1000	1	1	0.06	0.11	0.14	0.16	0.11	0.11	0.12	0.12	0.16	0.14
2	100	0.5	0	1.23	2.31	2.04	1.87	2.30	2.25	1.65	1.19	1.23	1.31
2	100	0.5	1	1.23	1.66	1.67	1.87	1.66	1.70	1.48	1.66	1.85	1.90
2	250	0.5	0	0.48	1.36	1.26	1.48	1.36	1.42	1.01	0.53	0.59	0.59
2	250	0.5	1	0.43	0.87	0.91	0.94	0.85	0.91	0.67	0.89	1.01	0.99
2	500	0.5	0	0.28	0.66	0.69	1.09	0.65	0.71	0.59	0.35	0.39	0.37
2	500	0.5	1	0.26	0.61	0.64	0.54	0.61	0.63	0.47	0.55	0.61	0.58
5	100	0.5	0	1.00	1.57	1.62	1.36	1.56	1.68	1.25	1.30	1.48	1.52
5	100	0.5	1	1.05	1.45	1.43	2.10	1.45	1.45	1.37	1.74	2.02	2.07
5	250	0.5	0	0.33	1.07	1.01	0.65	1.03	1.01	0.68	0.53	0.64	0.60
5	250	0.5	1	0.40	0.80	0.88	0.97	0.80	0.86	0.62	0.90	1.03	0.99
5	500	0.5	0	0.21	0.56	0.69	0.58	0.56	0.67	0.49	0.28	0.34	0.31
5	500	0.5	1	0.22	0.55	0.64	0.51	0.53	0.58	0.40	0.49	0.55	0.52
5	1000	0.5	0	0.17	0.31	0.43	0.45	0.30	0.36	0.34	0.20	0.21	0.21
5	1000	0.5	1	0.18	0.32	0.46	0.29	0.32	0.38	0.33	0.28	0.31	0.29
10	100	0.5	0	0.94	1.33	1.38	1.87	1.32	1.41	1.19	1.38	1.73	1.50
10	100	0.5	1	0.96	1.30	1.34	2.47	1.29	1.35	1.73	1.57	2.01	1.84
10	250	0.5	0	0.18	0.56	0.69	0.61	0.56	0.66	0.38	0.52	0.65	0.56
10	250	0.5	1	0.18	0.55	0.68	0.86	0.55	0.64	0.36	0.72	0.88	0.79
10	500	0.5	0	0.10	0.33	0.38	0.34	0.31	0.32	0.26	0.31	0.36	0.32
10	500	0.5	1	0.10	0.28	0.42	0.44	0.28	0.33	0.25	0.42	0.49	0.43
10	1000	0.5	0	0.08	0.17	0.26	0.20	0.17	0.20	0.20	0.17	0.19	0.18
10	1000	0.5	1	0.08	0.18	0.30	0.24	0.18	0.21	0.20	0.23	0.26	0.23

Table S11: Simulation results showing root mean squared distance of the logarithm of calibration slopes across simulation scenarios with marginal event rate $E(Y) = 0.25$ that differed by the number of predictors $K \in \{2, 5, 10\}$, sample size $N \in \{100, 250, 500, 1000\}$, effect multiplier $a \in \{1, 0.5\}$ and noise absent (0) or present (1). OP, prediction oracle; D, deviance; GCV, generalized cross-validation; CE, classification error; RCV50, repeated 10-fold cross-validated deviance with $\theta = 0.5$; RCV95, repeated 10-fold cross-validated deviance with $\theta = 0.95$; AIC, Akaike's information criterion; IP, shrinkage based on informative priors; WP, shrinkage based on weakly informative priors; FLIC, Firth's logistic regression with intercept-correction.

K	N	a	Noise	OP	D	GCV	CE	RCV50	RCV95	AIC	IP	WP	FLIC
2	100	1	0	0.48	1.00	0.59	1.64	1.00	0.98	0.46	0.29	0.27	0.28
2	100	1	1	0.18	0.50	0.82	0.57	0.49	0.64	0.41	0.34	0.51	0.44
2	250	1	0	0.22	0.51	0.35	1.10	0.51	0.52	0.30	0.19	0.20	0.20
2	250	1	1	0.15	0.51	0.43	0.49	0.50	0.38	0.30	0.20	0.26	0.22
2	500	1	0	0.12	0.25	0.21	0.75	0.25	0.26	0.19	0.13	0.15	0.14
2	500	1	1	0.08	0.26	0.26	0.37	0.26	0.27	0.21	0.15	0.18	0.17
5	100	1	0	0.20	0.46	0.64	0.75	0.42	0.48	0.38	0.24	0.37	0.32
5	100	1	1	0.14	0.46	0.80	0.56	0.46	0.58	0.39	0.45	0.65	0.57
5	250	1	0	0.14	0.55	0.32	0.63	0.56	0.54	0.30	0.17	0.22	0.20
5	250	1	1	0.09	0.33	0.42	0.35	0.27	0.26	0.25	0.25	0.34	0.29
5	500	1	0	0.11	0.26	0.22	0.49	0.26	0.27	0.19	0.13	0.16	0.15
5	500	1	1	0.07	0.27	0.25	0.25	0.27	0.27	0.20	0.17	0.21	0.18
5	1000	1	0	0.08	0.11	0.14	0.38	0.11	0.12	0.11	0.09	0.09	0.09
5	1000	1	1	0.08	0.11	0.16	0.22	0.11	0.12	0.11	0.09	0.09	0.09
10	100	1	0	0.13	0.34	0.63	0.54	0.34	0.41	0.38	0.27	0.50	0.45
10	100	1	1	0.14	0.38	0.73	0.74	0.38	0.46	0.49	0.44	0.74	0.67
10	250	1	0	0.09	0.19	0.30	0.30	0.19	0.20	0.21	0.16	0.25	0.22
10	250	1	1	0.08	0.19	0.37	0.33	0.19	0.21	0.21	0.23	0.34	0.29
10	500	1	0	0.06	0.13	0.19	0.20	0.13	0.13	0.13	0.11	0.14	0.13
10	500	1	1	0.06	0.13	0.23	0.20	0.12	0.14	0.14	0.14	0.18	0.15
10	1000	1	0	0.05	0.08	0.11	0.12	0.08	0.09	0.08	0.08	0.09	0.08
10	1000	1	1	0.05	0.08	0.13	0.13	0.08	0.09	0.09	0.09	0.11	0.10
2	100	0.5	0	0.61	1.41	1.47	1.60	1.40	1.56	1.13	0.66	0.70	0.70
2	100	0.5	1	0.58	1.03	1.18	0.96	1.03	1.13	0.83	0.99	1.11	1.08
2	250	0.5	0	0.25	0.52	0.72	1.10	0.51	0.63	0.53	0.30	0.31	0.31
2	250	0.5	1	0.17	0.53	0.73	0.46	0.53	0.62	0.46	0.42	0.47	0.43
2	500	0.5	0	0.18	0.29	0.41	0.75	0.29	0.34	0.32	0.22	0.22	0.22
2	500	0.5	1	0.14	0.31	0.51	0.38	0.31	0.37	0.33	0.24	0.26	0.25
5	100	0.5	0	0.31	0.95	1.07	0.75	0.91	1.00	0.67	0.70	0.83	0.81
5	100	0.5	1	0.36	0.82	0.90	1.06	0.82	0.87	0.66	1.11	1.29	1.23
5	250	0.5	0	0.11	0.45	0.65	0.55	0.44	0.54	0.41	0.35	0.39	0.37
5	250	0.5	1	0.11	0.44	0.59	0.49	0.43	0.48	0.35	0.56	0.62	0.58
5	500	0.5	0	0.11	0.24	0.42	0.46	0.24	0.29	0.27	0.20	0.22	0.21
5	500	0.5	1	0.10	0.26	0.44	0.29	0.26	0.31	0.27	0.30	0.32	0.30
5	1000	0.5	0	0.11	0.17	0.29	0.34	0.17	0.20	0.20	0.13	0.13	0.13
5	1000	0.5	1	0.10	0.18	0.33	0.20	0.18	0.22	0.22	0.16	0.17	0.16
10	100	0.5	0	0.22	0.70	0.90	0.80	0.69	0.80	0.49	0.82	1.02	0.91
10	100	0.5	1	0.23	0.68	0.83	1.07	0.67	0.74	0.53	1.08	1.34	1.22
10	250	0.5	0	0.11	0.35	0.64	0.38	0.35	0.43	0.34	0.32	0.37	0.33
10	250	0.5	1	0.12	0.36	0.64	0.43	0.36	0.44	0.31	0.45	0.52	0.46
10	500	0.5	0	0.08	0.19	0.38	0.24	0.19	0.23	0.22	0.20	0.22	0.20
10	500	0.5	1	0.07	0.20	0.41	0.26	0.20	0.24	0.22	0.28	0.31	0.27
10	1000	0.5	0	0.06	0.12	0.20	0.18	0.12	0.12	0.13	0.16	0.17	0.16
10	1000	0.5	1	0.05	0.13	0.24	0.18	0.13	0.13	0.14	0.20	0.21	0.20

Figure S1: Scatter plots showing the logarithm of calibration slopes obtained by optimizing different tuning criteria versus tuned complexity parameter values λ^* over 1000 generated datasets in scenarios with the expected value of Y, $E(Y) = 0.1$, the number of predictors $K = 5$, noise absent or present, the sample size of $N \in \{100, 250, 500, 1000\}$ considering A) moderate ($a = 0.5$) and B) strong ($a = 1$) predictors. The horizontal line indicates the calibration slope of 1. Red points refer to datasets where separation occurred. OP, prediction oracle, D, deviance; GCV, generalized cross-validation; CE, classification error; RCV50, repeated 10-fold cross-validated deviance with $\theta = 0.5$; RCV95, repeated 10-fold cross-validated deviance with $\theta = 0.95$; AIC, Akaike's information criterion.

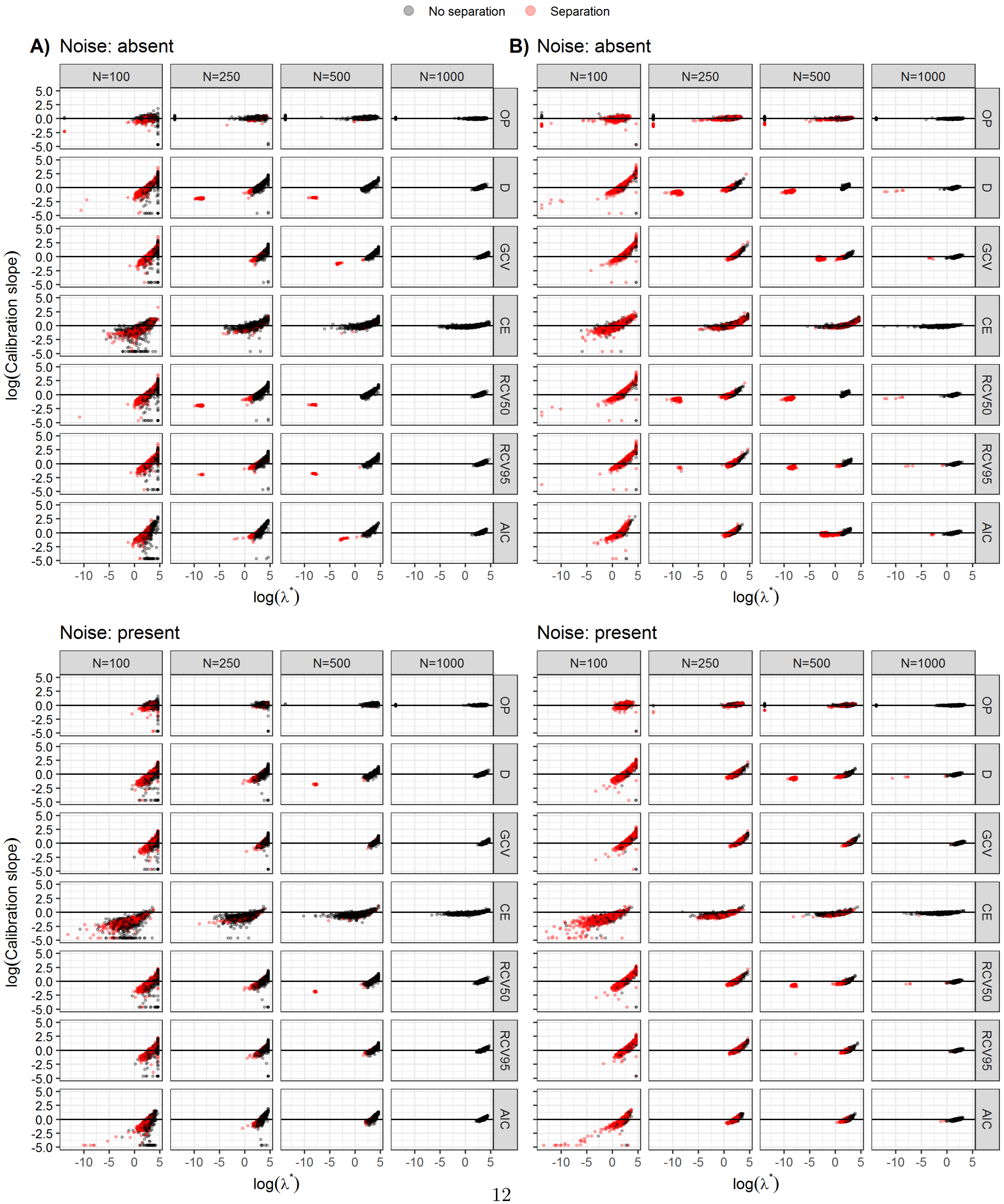

Table S12: Simulation results showing mean c-indices (with standard deviations) (×1000) across simulation scenarios with marginal event rate $E(Y) = 0.1$ that differed by the number of predictors $K \in \{2, 5, 10\}$, sample size $N \in \{100, 250, 500, 1000\}$, effect multiplier $a \in \{1, 0.5\}$ and noise absent (0) or present (1). Optimal c-index was calculated based on event probabilities from the true model. OP, prediction oracle; D, deviance; GCV, generalized cross-validation; CE, classification error; RCV50, repeated 10-fold cross-validated deviance with $\theta = 0.5$; RCV95, repeated 10-fold cross-validated deviance with $\theta = 0.95$; AIC, Akaike's information criterion; IP, shrinkage based on informative priors; WP, shrinkage based on weakly informative priors; FLIC, Firth's logistic regression with intercept-correction.

K	N	a	Noise	Optimal	OP	D	GCV	CE	RCV50	RCV95	AIC	IP	WP	FLIC
2	100	1	0	724 (70)	716 (75)	717 (74)	717 (74)	717 (74)	717 (75)	717 (74)	716 (75)	716 (75)	715 (75)	712 (76)
2	100	1	1	724 (70)	691 (86)	691 (86)	692 (86)	687 (87)	691 (86)	692 (86)	690 (86)	689 (87)	686 (87)	678 (89)
2	250	1	0	726 (44)	724 (45)	724 (45)	724 (45)	722 (44)	724 (45)	724 (45)	724 (45)	723 (45)	724 (45)	723 (45)
2	250	1	1	726 (44)	712 (52)	713 (52)	713 (52)	712 (52)	713 (52)	713 (52)	713 (52)	712 (52)	711 (52)	709 (53)
2	500	1	0	727 (30)	726 (31)	726 (31)	726 (31)	724 (31)	726 (31)	726 (31)	726 (31)	726 (31)	726 (31)	726 (31)
2	500	1	1	727 (30)	723 (35)	722 (36)	722 (36)	722 (36)	722 (36)	722 (36)	722 (36)	723 (36)	723 (35)	722 (36)
5	100	1	0	766 (74)	723 (88)	724 (88)	724 (88)	722 (89)	724 (88)	724 (88)	723 (88)	723 (88)	722 (88)	715 (90)
5	100	1	1	766 (74)	697 (89)	698 (89)	699 (89)	692 (89)	698 (89)	699 (89)	696 (89)	694 (88)	691 (88)	680 (89)
5	250	1	0	762 (45)	744 (48)	743 (48)	744 (49)	744 (49)	744 (48)	744 (49)	744 (48)	744 (48)	743 (48)	742 (49)
5	250	1	1	762 (45)	728 (52)	728 (52)	728 (52)	726 (52)	728 (52)	728 (52)	728 (52)	727 (52)	726 (52)	723 (53)
5	500	1	0	763 (33)	754 (34)	754 (34)	754 (34)	754 (34)	754 (34)	754 (34)	754 (34)	755 (34)	755 (34)	754 (34)
5	500	1	1	763 (33)	745 (35)	744 (35)	744 (35)	744 (35)	744 (35)	744 (35)	745 (35)	745 (35)	744 (35)	744 (35)
5	1000	1	0	762 (23)	758 (24)	758 (24)	758 (24)	758 (24)	758 (24)	758 (24)	758 (24)	759 (24)	759 (24)	758 (24)
5	1000	1	1	762 (23)	752 (24)	752 (24)	752 (24)	753 (24)	752 (24)	752 (24)	752 (24)	753 (24)	753 (25)	753 (25)
10	100	1	0	850 (65)	794 (86)	795 (86)	796 (86)	783 (92)	795 (86)	796 (86)	792 (88)	792 (88)	787 (90)	782 (90)
10	100	1	1	850 (65)	780 (92)	781 (91)	782 (91)	761 (96)	781 (91)	782 (91)	771 (95)	776 (93)	768 (95)	758 (96)
10	250	1	0	848 (41)	821 (46)	820 (46)	820 (46)	818 (47)	820 (46)	820 (46)	820 (46)	821 (46)	820 (47)	818 (47)
10	250	1	1	848 (41)	811 (48)	810 (48)	810 (48)	806 (49)	810 (48)	811 (48)	810 (48)	809 (48)	807 (48)	805 (49)
10	500	1	0	850 (29)	836 (31)	836 (31)	835 (31)	836 (31)	836 (31)	836 (31)	836 (31)	837 (31)	836 (31)	836 (31)
10	500	1	1	850 (29)	829 (33)	829 (33)	828 (33)	828 (33)	829 (33)	829 (33)	829 (33)	829 (33)	828 (33)	828 (33)
10	1000	1	0	848 (20)	841 (22)	841 (22)	840 (22)	841 (22)	841 (22)	841 (22)	841 (22)	841 (21)	841 (22)	841 (22)
10	1000	1	1	848 (20)	837 (22)	837 (22)	836 (22)	837 (22)	837 (22)	837 (22)	837 (22)	837 (22)	837 (22)	837 (22)
2	100	0.5	0	635 (75)	621 (75)	623 (74)	624 (74)	623 (75)	623 (75)	624 (74)	624 (74)	621 (75)	619 (75)	618 (75)
2	100	0.5	1	635 (75)	608 (74)	609 (74)	609 (75)	604 (73)	609 (74)	609 (75)	608 (74)	605 (74)	604 (74)	602 (73)
2	250	0.5	0	637 (50)	629 (56)	630 (55)	631 (55)	631 (54)	630 (55)	631 (55)	631 (55)	629 (57)	629 (57)	628 (56)
2	250	0.5	1	637 (50)	611 (58)	611 (58)	612 (58)	607 (58)	611 (58)	612 (58)	611 (58)	607 (58)	606 (58)	604 (57)
2	500	0.5	0	634 (38)	631 (40)	631 (40)	631 (40)	631 (39)	631 (40)	631 (40)	631 (40)	631 (40)	631 (40)	631 (40)
2	500	0.5	1	634 (38)	619 (44)	619 (44)	619 (44)	617 (45)	619 (44)	619 (44)	619 (44)	616 (45)	616 (45)	615 (45)
5	100	0.5	0	655 (80)	615 (76)	616 (76)	616 (77)	614 (76)	616 (76)	616 (77)	616 (76)	614 (76)	613 (76)	610 (75)
5	100	0.5	1	655 (80)	608 (76)	608 (77)	608 (77)	603 (75)	608 (77)	608 (77)	607 (76)	604 (75)	603 (74)	598 (73)
5	250	0.5	0	655 (53)	627 (57)	627 (57)	628 (57)	626 (57)	627 (57)	628 (57)	628 (57)	625 (58)	624 (58)	623 (58)
5	250	0.5	1	655 (53)	615 (57)	615 (57)	616 (57)	610 (57)	615 (57)	616 (57)	614 (57)	610 (57)	609 (57)	607 (57)
5	500	0.5	0	657 (37)	639 (42)	639 (42)	640 (42)	639 (42)	639 (42)	640 (42)	639 (42)	638 (43)	637 (43)	637 (43)
5	500	0.5	1	657 (37)	629 (43)	629 (43)	630 (43)	626 (44)	629 (43)	630 (43)	629 (43)	626 (44)	625 (44)	624 (44)
5	1000	0.5	0	659 (28)	649 (30)	649 (30)	650 (30)	649 (30)	649 (30)	649 (30)	649 (30)	649 (30)	649 (30)	648 (30)
5	1000	0.5	1	659 (28)	641 (30)	641 (30)	641 (30)	640 (31)	641 (30)	641 (30)	641 (30)	639 (31)	639 (31)	639 (31)
10	100	0.5	0	723 (88)	660 (92)	661 (92)	662 (92)	652 (90)	661 (92)	662 (93)	659 (93)	655 (91)	651 (90)	650 (90)
10	100	0.5	1	723 (88)	651 (88)	651 (88)	652 (89)	637 (86)	651 (88)	652 (89)	648 (87)	643 (87)	638 (86)	636 (85)
10	250	0.5	0	720 (56)	679 (62)	680 (62)	681 (62)	675 (63)	680 (62)	681 (62)	680 (62)	675 (63)	674 (63)	674 (62)
10	250	0.5	1	720 (56)	670 (64)	670 (64)	672 (64)	662 (65)	671 (64)	671 (64)	670 (64)	663 (65)	661 (65)	660 (65)
10	500	0.5	0	718 (37)	694 (41)	694 (41)	695 (41)	692 (42)	694 (41)	695 (41)	694 (41)	692 (42)	692 (42)	692 (42)
10	500	0.5	1	718 (37)	685 (42)	686 (42)	686 (42)	681 (43)	686 (42)	686 (42)	686 (42)	681 (43)	680 (43)	680 (43)
10	1000	0.5	0	718 (27)	705 (29)	704 (29)	704 (29)	704 (29)	704 (29)	704 (29)	704 (29)	704 (29)	704 (29)	704 (29)
10	1000	0.5	1	718 (27)	699 (30)	699 (30)	698 (30)	698 (30)	699 (30)	699 (30)	699 (30)	697 (30)	697 (30)	697 (30)

Table S13: Simulation results showing mean c-indices (with standard deviations) ($\times 1000$) across simulation scenarios with marginal event rate $E(Y) = 0.25$ that differed by the number of predictors $K \in \{2, 5, 10\}$, sample size $N \in \{100, 250, 500, 1000\}$, effect multiplier $a \in \{1, 0.5\}$ and noise absent (0) or present (1). Optimal c-index was calculated based on event probabilities from the true model. OP, prediction oracle; D, deviance; GCV, generalized cross-validation; CE, classification error; RCV50, repeated 10-fold cross-validated deviance with $\theta = 0.5$; RCV95, repeated 10-fold cross-validated deviance with $\theta = 0.95$; AIC, Akaike's information criterion; IP, shrinkage based on informative priors; WP, shrinkage based on weakly informative priors; FLIC, Firth's logistic regression with intercept-correction.

K	N	a	Noise	Optimal	OP	D	GCV	CE	RCV50	RCV95	AIC	IP	WP	FLIC
2	100	1	0	737 (50)	735 (51)	735 (51)	734 (51)	733 (51)	735 (51)	735 (51)	735 (51)	735 (51)	735 (51)	734 (51)
2	100	1	1	737 (50)	720 (62)	721 (61)	722 (61)	719 (62)	721 (61)	722 (61)	721 (61)	718 (62)	716 (63)	715 (63)
2	250	1	0	736 (31)	735 (32)	735 (31)	735 (32)	733 (31)	735 (31)	735 (31)	735 (31)	735 (31)	735 (31)	735 (31)
2	250	1	1	736 (31)	732 (36)	731 (36)	731 (36)	731 (36)	731 (36)	731 (36)	731 (36)	731 (36)	731 (36)	731 (36)
2	500	1	0	736 (22)	735 (22)	735 (22)	735 (22)	732 (23)	735 (22)	735 (23)	735 (22)	735 (22)	735 (22)	735 (22)
2	500	1	1	736 (22)	734 (25)	734 (25)	733 (25)	733 (25)	734 (25)	734 (25)	734 (25)	734 (25)	734 (25)	734 (25)
5	100	1	0	768 (50)	746 (57)	746 (57)	746 (57)	746 (57)	746 (57)	746 (57)	746 (57)	746 (57)	745 (57)	743 (58)
5	100	1	1	768 (50)	731 (59)	731 (58)	732 (58)	728 (59)	731 (59)	731 (58)	731 (59)	727 (60)	725 (60)	722 (60)
5	250	1	0	768 (33)	760 (34)	760 (34)	760 (34)	759 (35)	760 (34)	760 (34)	760 (34)	760 (34)	760 (34)	760 (34)
5	250	1	1	768 (33)	750 (35)	750 (35)	750 (35)	749 (35)	750 (35)	750 (35)	750 (35)	749 (35)	749 (35)	748 (35)
5	500	1	0	769 (24)	765 (25)	765 (24)	765 (24)	764 (24)	765 (24)	765 (24)	765 (24)	765 (24)	765 (24)	765 (24)
5	500	1	1	769 (24)	759 (25)	759 (25)	758 (25)	758 (25)	759 (25)	759 (25)	759 (25)	759 (25)	759 (25)	759 (25)
5	1000	1	0	769 (15)	767 (16)	767 (16)	767 (16)	766 (16)	767 (16)	767 (16)	767 (16)	767 (16)	767 (16)	767 (16)
5	1000	1	1	769 (15)	764 (16)	764 (16)	764 (16)	763 (16)	764 (16)	764 (16)	764 (16)	764 (16)	764 (16)	764 (16)
10	100	1	0	830 (47)	794 (54)	794 (54)	793 (55)	792 (55)	794 (54)	794 (55)	794 (54)	794 (54)	792 (54)	789 (55)
10	100	1	1	830 (47)	781 (56)	780 (57)	780 (57)	777 (58)	780 (57)	780 (57)	780 (57)	778 (56)	775 (57)	771 (57)
10	250	1	0	825 (28)	808 (30)	808 (31)	807 (31)	808 (31)	808 (31)	808 (31)	808 (31)	808 (31)	808 (31)	808 (31)
10	250	1	1	825 (28)	801 (32)	801 (32)	800 (32)	800 (32)	801 (32)	800 (32)	800 (32)	800 (32)	800 (32)	799 (32)
10	500	1	0	826 (19)	818 (20)	817 (20)	817 (20)	817 (20)	817 (20)	817 (20)	817 (20)	817 (20)	817 (20)	817 (20)
10	500	1	1	826 (19)	813 (20)	813 (20)	812 (21)	812 (20)	813 (20)	813 (20)	813 (20)	813 (20)	813 (20)	812 (20)
10	1000	1	0	825 (15)	821 (15)	821 (15)	821 (15)	821 (15)	821 (15)	821 (15)	821 (15)	821 (15)	821 (15)	821 (15)
10	1000	1	1	825 (15)	819 (15)	819 (15)	818 (15)	819 (15)	819 (15)	819 (15)	819 (15)	819 (15)	819 (15)	819 (15)
2	100	0.5	0	642 (57)	631 (61)	633 (60)	633 (60)	634 (60)	633 (60)	633 (60)	633 (60)	630 (61)	629 (61)	628 (61)
2	100	0.5	1	642 (57)	616 (63)	616 (63)	617 (63)	613 (63)	616 (63)	617 (63)	616 (63)	611 (62)	610 (62)	608 (62)
2	250	0.5	0	640 (37)	638 (38)	637 (38)	638 (38)	638 (37)	637 (38)	638 (38)	637 (38)	637 (39)	637 (39)	637 (39)
2	250	0.5	1	640 (37)	627 (42)	627 (42)	628 (42)	625 (43)	627 (42)	627 (42)	627 (42)	623 (43)	623 (43)	623 (43)
2	500	0.5	0	640 (25)	639 (26)	639 (26)	639 (26)	639 (25)	639 (26)	639 (26)	639 (26)	639 (26)	639 (26)	639 (26)
2	500	0.5	1	640 (25)	634 (29)	634 (29)	635 (28)	634 (29)	634 (29)	635 (29)	635 (29)	633 (29)	633 (29)	633 (29)
5	100	0.5	0	661 (60)	630 (62)	630 (63)	632 (63)	629 (63)	631 (63)	632 (63)	631 (62)	627 (62)	626 (62)	624 (63)
5	100	0.5	1	661 (60)	617 (62)	618 (61)	619 (62)	612 (61)	618 (61)	618 (61)	617 (61)	611 (61)	608 (61)	607 (60)
5	250	0.5	0	661 (39)	645 (41)	646 (41)	647 (40)	645 (41)	646 (41)	646 (41)	646 (41)	644 (41)	643 (41)	643 (41)
5	250	0.5	1	661 (39)	635 (42)	635 (42)	636 (42)	632 (42)	635 (42)	635 (41)	635 (42)	631 (42)	630 (42)	629 (42)
5	500	0.5	0	661 (27)	654 (28)	653 (28)	654 (28)	654 (28)	653 (28)	653 (28)	653 (28)	653 (28)	653 (28)	653 (28)
5	500	0.5	1	661 (27)	646 (28)	646 (29)	647 (28)	645 (29)	646 (29)	646 (28)	646 (28)	644 (29)	644 (29)	644 (29)
5	1000	0.5	0	661 (19)	657 (20)	657 (20)	657 (20)	657 (20)	657 (20)	657 (20)	657 (20)	657 (20)	657 (20)	657 (20)
5	1000	0.5	1	661 (19)	652 (20)	652 (20)	652 (20)	652 (21)	652 (20)	652 (20)	652 (20)	651 (21)	651 (21)	651 (21)
10	100	0.5	0	703 (62)	653 (68)	653 (68)	654 (68)	649 (68)	653 (68)	653 (68)	652 (68)	649 (69)	647 (69)	646 (69)
10	100	0.5	1	703 (62)	643 (68)	644 (68)	644 (68)	638 (66)	644 (68)	644 (68)	643 (68)	637 (67)	635 (66)	633 (66)
10	250	0.5	0	705 (39)	680 (41)	680 (41)	680 (41)	679 (42)	680 (41)	680 (41)	681 (41)	679 (42)	679 (42)	678 (42)
10	250	0.5	1	705 (39)	672 (42)	672 (42)	672 (42)	669 (43)	672 (42)	672 (42)	672 (42)	669 (43)	668 (43)	667 (43)
10	500	0.5	0	705 (27)	690 (28)	690 (28)	689 (28)	689 (28)	690 (28)	690 (28)	690 (28)	690 (28)	689 (28)	689 (28)
10	500	0.5	1	705 (27)	684 (28)	683 (28)	683 (29)	683 (28)	683 (28)	683 (28)	683 (28)	682 (29)	682 (29)	682 (29)
10	1000	0.5	0	705 (20)	697 (20)	697 (20)	697 (20)	697 (20)	697 (20)	697 (20)	697 (20)	697 (20)	697 (20)	697 (20)
10	1000	0.5	1	705 (20)	694 (20)	693 (20)	693 (20)	693 (20)	693 (20)	693 (20)	693 (20)	693 (20)	693 (21)	693 (21)

R code

```
library(brglm2)
library(logistf)
library(penalized)
library(simdata)
library(doParallel)
library(doRNG)

#Illustrative example ################################################################################

#Dataset 1
x=c(rep(0, 20), rep(1, 80))
y=c(rep(0, 20), rep(0, 71), rep(1, 9))
table(x,y)

df <- data.frame(x=x, y=y)
f <- logistf(y~x, data=df, flic=T)
cfs.f <- coef(f)
table(f$predict)

fit.opt.1<-profL2(y, x, lambda1 = 0, minlambda2=1e-6, maxlambda2=100, steps=200, minsteps=200, log=TRUE,
                  fusedl = FALSE, positive = FALSE, model = "logistic", fold=length(y),
                  standardize = TRUE, save.predictions = TRUE, trace = FALSE, plot = FALSE, approximate = F)

l.opt = fit.opt.1$lambda[which.max(fit.opt.1$cvl)]
cfs.dev <- coef(penalized(y, penalized = ~x, lambda2 = l.opt, standardize = T))
table(fitted(penalized(y, penalized = ~x, lambda2 = l.opt, standardize = T)))

cfs.ip <- coef(penalized(y, penalized = ~x, lambda2 = 2, standardize = T))
table(fitted(penalized(y, penalized = ~x, lambda2 = 2, standardize = T)))

#Dataset 2
x=c(rep(0, 20), rep(1, 80))
y=c(rep(0, 19), 1, rep(0, 71), rep(1, 9))
table(x,y)

df <- data.frame(x=x, y=y)
f <- logistf(y~x, data=df, flic=T)
cfs.f <- coef(f)
table(f$predict)

fit.opt.1<-profL2(y, x, lambda1 = 0, minlambda2=1e-6, maxlambda2=100, steps=200, minsteps=200, log=TRUE,
                  fusedl = FALSE, positive = FALSE, model = "logistic", fold=length(y),
                  standardize = TRUE, save.predictions = TRUE, trace = FALSE, plot = FALSE, approximate = F)

l.opt = fit.opt.1$lambda[which.max(fit.opt.1$cvl)]
cfs.dev <- coef(penalized(y, penalized = ~x, lambda2 = l.opt, standardize = T))
table(fitted(penalized(y, penalized = ~x, lambda2 = l.opt, standardize = T)))

cfs.ip <- coef(penalized(y, penalized = ~x, lambda2 = 2, standardize = T))
table(fitted(penalized(y, penalized = ~x, lambda2 = 2, standardize = T)))

#Repeat data-generating mechanism 500x
res = matrix(NA, 500, 7)
colnames(res) <- c("ym", "opt.rr.dev.1", "calib.p.rr.dev.1", "cfs.f", "calib.p.f", "cfs.rr.ip", "calib.p.rr.ip")

betas=c(-3.053194, 1)
set.seed(123)
testX = cbind(1, sample(c(0,1), prob=c(0.2, 0.8), 10000, replace=T))
testlp = testX %*% betas
testY = ifelse(runif(nrow(testX))<1/(1+exp(-testlp)), 1, 0)

set.seed(2828)
for(i in 1:500){

  #generate x
  x=sample(c(0,1), prob=c(0.2, 0.8), 100, replace=T)
  #linear predictor
  lp = cbind(1, x)%*%betas
  #generate y
  y=ifelse(runif(length(x))<1/(1+exp(-lp)), 1, 0)
  df = data.frame(y=y, x=x)
  res[i, "ym"] = mean(y)

  #FC
  res[i,"cfs.f"] <- coef(logistf(y~x, data=df, flic=T))[2]
  res[i,"calib.p.f"] <- coef(glm(testY~c(testX %*% coef(logistf(y~x, data=df, flic=T))), family = "binomial"))[2]


  #LOOCV D
  fit.opt.1<-profL2(y, x, lambda1 = 0, minlambda2=1e-6, maxlambda2=100, steps=200, minsteps=200, log=TRUE,
                    fusedl = FALSE, positive = FALSE,  model = "logistic", fold=length(y),
                    standardize = TRUE, save.predictions = TRUE, trace = FALSE, plot = FALSE, approximate = F)
```

```
  res[i,"opt.rr.dev.1"] <- fit.opt.1$lambda[which.max(fit.opt.1$cvl)]
  cfs.rr.dev.1 <- coef(penalized(y, penalized = ~x,
                     lambda2 = fit.opt.1$lambda[which.max(fit.opt.1$cvl)], standardize = T))
  res[i,"calib.p.rr.dev.1"] <- coef(glm(testY~c(testX %*% cfs.rr.dev.1), family = "binomial"))[2]


  #IP
  cfs.rr.ip <- coef(penalized(y, penalized = ~x, lambda2 = 2, standardize = T))
  res[i,"calib.p.rr.ip"] <- coef(glm(testY~c(testX %*% cfs.rr.ip), family = "binomial"))[2]
  res[i,"cfs.rr.ip"] <- cfs.rr.ip[2]

}

#Helper functions ####################################################################################

#' @title Generate sample
#' @param n
#' sample size
#' @param sim_design
#' stored simulation design
#' @param n.var
#' number of true predictors
#' @param target.prop
#' expected value of Y
#' @param betas
#' a vector of parameter values without the intercept
#' @param beta0.opt
#' intercept

my.sample <- function(n, sim_design, n.var, target.prop, betas, beta0.opt) {
  X <- as.matrix(simulate_data(sim_design, n_obs = n))
  beta <- c(beta0.opt, betas[c(1:n.var, 11:15)])
  names(beta)[1] <- "Intercept"
  lp = cbind(1, X[, c(1:n.var, 11:15)])%*%beta
  y=ifelse(runif(n)<1/(1+exp(-lp)), 1, 0)
  data <- data.frame(y=y, X[,c(1:n.var, 11:15)])
  return(list(data=data, beta=beta))
}

#' @title Calculate c-index
#' @param x
#' a numeric vector of values
#' @param y
#' a binary vector of values

#c-index
cindex<-function(x,y, abs=TRUE) {
  c1<-wilcox.test(x~y)$statistic/sum(y==0)/sum(y==1)
  if(abs){
    if(c1<0.5) c1<-1-c1
  }
  return(c1)
}


#' @title Standardize covariates
#' @param X
#' model matrix (+/- the intercept)
#' @param type
#' type=1, standardization to zero mean and unit variance, type=2, Gelman's standardization

my.std<-function(X, type=1){
  n<-nrow(X)
  p<-ncol(X)
  if (sum(X[,1])==n) intercept=TRUE else intercept=FALSE

  if (type==1){
    ms<-apply(X,2,mean)
    ss<-apply(X,2,sd)

    X<-(X-matrix(ms,ncol=p,nrow=n,byrow=T))/matrix(ss,ncol=p,nrow=n,byrow=T)
    binom<-rep(0,p)

  } else {

    binom<-apply(X,2,function(x) ifelse(length(unique(x))==2,1,0) )

    xb<-as.matrix(X[,which(binom==1)],ncol=sum(binom),nrow=n,byrow=T)

    ms<-apply(X,2,mean)
    ss<-apply(X,2,sd)*2

    X<-(X-matrix(ms,ncol=p,nrow=n,byrow=T))/matrix(ss,ncol=p,nrow=n,byrow=T)

    xbs<-apply(xb,2,function(x) x-mean(x) )
```

```
    X[,which(binom==1)]<-xbs
  }

  if (intercept==TRUE) X[,1]<-1

  list(X=X,means=ms,sds=ss,binom=binom)
}

#' @title Unstandardize coefficents
#' @param beta
#' a vector of coefficients
#' @param z
#' output of my.std

my.unstandardize<-function(beta, z){
  X<-z$X
  n<-nrow(X)
  p<-ncol(X)
  if (sum(X[,1])==n) intercept=TRUE else intercept=FALSE

  betao<-beta/z$sds
  adjb<-beta*z$means/z$sds
  adjb[which(z$binom==1)]<-(beta*z$means)[which(z$binom==1)]

  if (intercept==TRUE) betao[1]<-beta[1]-sum(adjb[-1])

  betao[which(z$binom==1)]<-beta[which(z$binom==1)]
  betao
}

#' @title Fit ridge logistic regression by data augmentation
#' @param data
#' a data frame containing the values in the formula
#' @param formula
#' a model formula
#' @param priorv
#' prior variance
#' @param penalize
#' apply penalization to which parameters
#' @param s
#' scaling factor
#' @param intercept
#' including the intercept

logistr<-function(data, formula, priorv, penalize, s=10, intercept=TRUE){
  penvars <- sum(penalize)
  mf <- model.frame(data=data, formula=formula)
  y <- model.response(mf)
  x <- model.matrix(mf,data=data, formula=formula)
  varnames <- colnames(x)
  realdata <- cbind(x,y, weight=rep(1,nrow(x)))

  pseudodata <- matrix(0,2*(penvars),length(varnames)+2)
  colnames(pseudodata) <- c(varnames,"y","weight")
  pseudodata[,"y"] <- rep(c(0,1), (penvars))
  j=1
  for(i in which(penalize!=0)) {
    j=j+1
    pseudodata[(j-2)*2+(1:2),varnames[i]]<-1/s
  }

  pseudodata[,"weight"] <- 2*s*s/priorv
  pseudodata <- as.data.frame(pseudodata)
  newdata <- rbind(realdata, pseudodata)
  if (intercept==TRUE) newdata$Intercept <- newdata[,"(Intercept)"]
  if (intercept==TRUE) formula2 <- as.formula(paste(formula, "+Intercept-1")) else formula2<-as.formula(paste(formula))

  fit.ridge <- logistf(data=newdata, formula=formula2, firth=FALSE, weights=weight, dataout=TRUE)
  fit.ridge$call$formula <- formula2
  return(fit.ridge)
}

#' @title Deviance
#' @param i
#' evaluated at which lambda
#' @est.p
#' cross-validated probabilities at lambda i
#' @param y
#' binary outcome

my.dev <- function(i, est.p, y){
  -2*sum( y*log(est.p[[i]])+(1-y)*log(1-est.p[[i]])  )
}

#' @title Generalized cross-validation
#' @param i
```

```
#' evaluated at which lambda
#' @est.p
#' cross-validated probabilities at lambda i
#' @param y
#' binary outcome
#' @param edf
#' effective degrees of freedom

my.dev.g <- function(i, est.p, y, edf){
  (length(y)*(-2)*sum( y*log(est.p[[i]])+(1-y)*log(1-est.p[[i]])  ) ) / (length(y)-edf[i])^2

}

#' @title Mean classification error
#' @param i
#' evaluated at which lambda
#' @est.p
#' cross-validated probabilities at lambda i
#' @param y
#' binary outcome

my.error <- function(i, est.p, y){
  mean(y*ifelse(est.p[[i]]<mean(y),1,0)+(1-y)*ifelse(est.p[[i]]>mean(y),1,0)+0.5*ifelse(est.p[[i]]==mean(y),1,0))
}

#' @title Explanation oracle
#' @param i
#' evaluated at which lambda
#' @param coef.rr
#' standardized coefficients at lambda i
#' @param xz
#' output of my.std
#' @param beta
#' true coefficients
#' @param j
#' with respect to which coefficient

##oracle
my.oracle <- function(i, coef.rr, xz, beta, j){
  (my.unstandardize(coef.rr[[i]], xz)[j]-beta[j])^2
}

#' @title Prediciton oracle
#' @param i
#' evaluated at which lambda
#' @predictions
#' fitted predictions at lambda i
#' @param ps
#' true predictions

my.oracle.p <- function(i, predictions, ps){
  mean(c(predictions[[i]]-ps)^2)
}

#' @title AIC
#' @param l
#' a vector of lambda values
#' @param y
#' binray outcome
#' @param X
#' model matrix
#' @param penalize
#' apply penalization to which parameters; if NULL it applys to all non-intercept parameters

my.aic.ridge <- function(l, y, X, penalize=NULL)   {

  n<-nrow(X)
  p<-ncol(X)
  if (is.null(penalize)) {penalize<-rep(1,p);penalize[1]<-0}

  prob_min = 1e-05
  prob_max = 1 - prob_min

  aic=NULL
  edf=NULL
  cfs.rr <- list()
  probs <- list()

  for(i in 1:length(l)){
    fit.aic <- penalized(y, X[,-1], lambda2=l[i], fusedl = FALSE, positive = FALSE,
                         model =   "logistic", standardize = FALSE, trace = FALSE)
    cfs.rr[[i]] <- coef(fit.aic)
    pi<- pmin(pmax(fitted(fit.aic) , prob_min), prob_max)
    deviance<- -2*sum(y*log(pi)+(1-y)*log(1-pi))

    W<-matrix(0,ncol=n,nrow=n)
```

```
    diag(W)<-pi-pi**2

    I<-matrix(0,ncol=p,nrow=p)
    diag(I) <- penalize

    XWX<-t(X)%*%W%*%X

    XWXI.inv<-solve(XWX+l[i]*I)

    v<-XWXI.inv%*%XWX%*%XWXI.inv
    edf[i]=sum(diag(XWX %*% v)) - 1

    aic[i] = deviance+2*edf[i]

    probs[[i]] <- pi

  }

  return(list(aic, edf, cfs.rr, probs))

}


# Simulation function ###############################################################################

#' @title Simulation loop
#' @param nRep
#' number of simulation runs
#' @param n
#' sample size
#' @param n.var
#' number of true predictors
#' @param target.prop
#' expected value of Y
#' @param betas
#' true non-intercept coefficients
#' @param beta0.opt
#' intercept
#' @param sim_design
#' stored simulation parameters
#' @param testX
#' model matrix of a validation dataset
#' @param testY
#' binary outcome of a validation dataset

sim <- function(nRep, n, n.var, target.prop, betas, beta0.opt, sim_design, testX, testY) {

  ResMat <- foreach(i=1:nRep, .combine = rbind,
                  .packages=c("logistf", "brglm2", "penalized", "simdata"),
                  .errorhandling=c('remove'),
                  .export=c("my.sample", "cindex", "my.dev", "my.dev.g",  "my.error", "my.oracle", "my.oracle.p",
                            "logistr", "my.std", "my.unstandardize", "my.aic.ridge"))  %dorng% {

                    #generate sample
                    smp <- my.sample(n=n, sim_design=sim_design, n.var=n.var, target.prop=target.prop,
                                     betas=betas, beta0.opt=beta0.opt)
                    df <- as.matrix(smp$data)

                    #save betas
                    beta <- smp$beta

                    #define covariates
                    x <- as.matrix(df[,-1])
                    #define outcome
                    y <- df[,1]
                    #mean outcome
                    ys <- mean(y)

                    #a vector of true probabilities
                    ps <- (1 + exp(-beta[1] - x %*% (beta[-1])))^(-1)
                    #linear predictor vector
                    lps <- beta[1] + x %*% (beta[-1])

                    #true c-stat with respect to new y
                    new.y <- rep(0, n)
                    while (sum(new.y)<1) {
                      new.y <- rbinom(n, size=1, prob=ps)
                    }
                    cs <- cindex(ps, new.y)

                    #detect separation
                    sep <- ifelse(glm(y~x, family="binomial", method = "detect_separation", purpose="test",
                                      linear_program = "dual")$separation==T, 1, 0)
```

```
#Noise absent
p <- dim(x)[2]-5
xx=cbind(1,x[,1:p])

###Firth
df <- data.frame(y, x[,1:p])
formulas <- as.formula(paste("y~",paste(names(df)[-1] ,collapse="+"),sep=""))
firth <- logistf(formulas, df, flic=T)

cfs.f <- c(firth$coef)
ps.f <- c(firth$predict)
lps.f <- c(firth$linear.predictors)
calib.p.f <- c(coef(glm(testY~c(testX[,1:(p+1)] %*% cfs.f), family = "binomial"))[2])
cs.f <- cindex(lps.f, new.y)

###Ridge

#standardize covariates to zero mean and unit variance
xz<-my.std(xx, type=1)
xs<-xz$X

df<-data.frame(y, xs[,-1])
formulas<-as.formula(paste("y~",paste(names(df)[-1] ,collapse="+"),sep=""))

pen<-c(0,rep(1,p))

#LOOCV predicted probabilities
fit.opt.1 <- profL2(y, xs[,-1], lambda1 = 0, minlambda2=1e-6, maxlambda2=100, steps=200,
                    minsteps=200, log=TRUE, fused1 = FALSE, positive = FALSE,  model =   "logistic" ,
                    fold=n, standardize = FALSE,  save.predictions = TRUE,
                    trace = FALSE, plot = FALSE, approximate = F)


prob_min <- 1e-05
prob_max <- 1 - prob_min

fit.opt.1$predictions <- lapply(fit.opt.1$predictions, function(x) pmin(pmax(x, prob_min), prob_max))

##AIC
aic = my.aic.ridge(l=fit.opt.1$lambda, y=y, X=xs, penalize=NULL)
coef.rr <- aic[[3]]
edf <- aic[[2]]
probs <- aic[[4]]

opt.rr.aic <- fit.opt.1$lambda[which.min(aic[[1]])]
fit.rr.aic <- logistr(df, formulas,penalize=pen, priorv=1/opt.rr.aic, s=10)
fit.rr.aic$coef <- fit.rr.aic$coef[c(p+1,1:p)]
cfs.rr.aic <- my.unstandardize(fit.rr.aic$coef, xz)
ps.rr.aic <- fit.rr.aic$predict[1:n]
lps.rr.aic <- fit.rr.aic$linear.predictors[1:n]
calib.p.rr.aic <- c(coef(glm(testY~c(testX[,1:(p+1)] %*% cfs.rr.aic), family = "binomial"))[2])
cs.rr.aic <- cindex(lps.rr.aic, new.y)

##D
dev.1 <- unlist(lapply(1:200, my.dev,fit.opt.1$predictions, y))
opt.rr.dev.1 <- fit.opt.1$lambda[which.min(dev.1)]
fit.rr.dev.1 <- logistr(df, formulas,penalize=pen, priorv=1/opt.rr.dev.1, s=10)
fit.rr.dev.1$coef <- fit.rr.dev.1$coef[c(p+1,1:p)]
cfs.rr.dev.1 <- my.unstandardize(fit.rr.dev.1$coef, xz)
ps.rr.dev.1 <- fit.rr.dev.1$predict[1:n]
lps.rr.dev.1 <- fit.rr.dev.1$linear.predictors[1:n]
calib.p.rr.dev.1 <- c(coef(glm(testY~c(testX[,1:(p+1)] %*% cfs.rr.dev.1), family = "binomial"))[2])
cs.rr.dev.1 <- cindex(lps.rr.dev.1, new.y)

##GCV
dev.g.1 <- unlist(lapply(1:200, my.dev.g, fit.opt.1$predictions, y, edf))
opt.rr.dev.g.1 <- fit.opt.1$lambda[which.min(dev.g.1)]
fit.rr.dev.g.1 <- logistr(df, formulas,penalize=pen, priorv=1/opt.rr.dev.g.1, s=10)
fit.rr.dev.g.1$coef <- fit.rr.dev.g.1$coef[c(p+1,1:p)]
cfs.rr.dev.g.1 <- my.unstandardize(fit.rr.dev.g.1$coef, xz)
ps.rr.dev.g.1 <- fit.rr.dev.g.1$predict[1:n]
lps.rr.dev.g.1 <- fit.rr.dev.g.1$linear.predictors[1:n]
calib.p.rr.dev.g.1 <- c(coef(glm(testY~c(testX[,1:(p+1)] %*% cfs.rr.dev.g.1), family = "binomial"))[2])
cs.rr.dev.g.1 <- cindex(lps.rr.dev.g.1, new.y)

##CE
ce.1 <- unlist(lapply(1:200, my.error, fit.opt.1$predictions, y))
opt.rr.ce.1 <- fit.opt.1$lambda[which.min(ce.1)]
fit.rr.ce.1 <- logistr(df, formulas,penalize=pen, priorv=1/opt.rr.ce.1, s=10)
fit.rr.ce.1$coef <- fit.rr.ce.1$coef[c(p+1,1:p)]
cfs.rr.ce.1 <- my.unstandardize(fit.rr.ce.1$coef,xz)
ps.rr.ce.1 <- fit.rr.ce.1$predict[1:n]
lps.rr.ce.1 <- fit.rr.ce.1$linear.predictors[1:n]
calib.p.rr.ce.1 <- c(coef(glm(testY~c(testX[,1:(p+1)] %*% cfs.rr.ce.1), family = "binomial"))[2])
cs.rr.ce.1 <- cindex(lps.rr.ce.1, new.y)
```

```
##repeated 10-fold CV
opt.dev.10=NULL
for(j in 1:50){
  fit.opt.10 <- profL2(y, xs[,-1], lambda1 = 0, minlambda2=1e-6, maxlambda2=100, steps=200,
                      minsteps=200, log=TRUE, fusedl = FALSE, positive = FALSE,  model =   "logistic" ,
                      fold=10, standardize = FALSE,  save.predictions = T, trace = FALSE, plot = FALSE,
                      approximate = F)
  fit.opt.10$predictions <- lapply(fit.opt.10$predictions, function(x) pmin(pmax(x, prob_min), prob_max))
  dev.10 <- unlist(lapply(1:200,my.dev,fit.opt.10$predictions,y))
  opt.dev.10[j] <- fit.opt.10$lambda[which.min(dev.10)]
}

##RCV50
opt.rr.perc.50 <- median(opt.dev.10)
fit.rr.perc.50 <- logistr(df, formulas, penalize=pen, priorv=1/opt.rr.perc.50, s=10)
fit.rr.perc.50$coef <- fit.rr.perc.50$coef[c(p+1,1:p)]
cfs.rr.perc.50 <- my.unstandardize(fit.rr.perc.50$coef, xz)
ps.rr.perc.50 <- fit.rr.perc.50$predict[1:n]
lps.rr.perc.50 <- fit.rr.perc.50$linear.predictors[1:n]
calib.p.rr.perc.50 <- c(coef(glm(testY~c(testX[,1:(p+1)] %*% cfs.rr.perc.50), family = "binomial"))[2])
cs.rr.perc.50 <- cindex(lps.rr.perc.50, new.y)

##RCV95
opt.rr.perc.95 <- quantile(opt.dev.10, c(0.95))
fit.rr.perc.95 <- logistr(df, formulas, penalize=pen, priorv=1/opt.rr.perc.95, s=10)
fit.rr.perc.95$coef<-fit.rr.perc.95$coef[c(p+1,1:p)]
cfs.rr.perc.95 <- my.unstandardize(fit.rr.perc.95$coef, xz)
ps.rr.perc.95 <- fit.rr.perc.95$predict[1:n]
lps.rr.perc.95 <- fit.rr.perc.95$linear.predictors[1:n]
calib.p.rr.perc.95 <- c(coef(glm(testY~c(testX[,1:(p+1)] %*% cfs.rr.perc.95), family = "binomial"))[2])
cs.rr.perc.95 <- cindex(lps.rr.perc.95, new.y)

##explanation oracle
oracle <- unlist(lapply(1:200, my.oracle, coef.rr, xz, beta, j=2))
opt.rr.oracle <- fit.opt.1$lambda[which.min(oracle)]
fit.rr.oracle <- logistr(df, formulas, penalize=pen, priorv=1/opt.rr.oracle, s=10)
fit.rr.oracle$coef<-fit.rr.oracle$coef[c(p+1,1:p)]
cfs.rr.oracle <- my.unstandardize(fit.rr.oracle$coef, xz)
ps.rr.oracle <- fit.rr.oracle$predict[1:n]
lps.rr.oracle <- fit.rr.oracle$linear.predictors[1:n]
calib.p.rr.oracle <- c(coef(glm(testY~c(testX[,1:(p+1)] %*% cfs.rr.oracle), family = "binomial"))[2])
cs.rr.oracle <- cindex(lps.rr.oracle, new.y)

##prediction oracle
oracle.p <- unlist(lapply(1:200, my.oracle.p, predictions=probs, ps=ps))
opt.rr.oracle.p <- fit.opt.1$lambda[which.min(oracle.p)]
fit.rr.oracle.p <- logistr(df, formulas, penalize=pen, priorv=1/opt.rr.oracle.p, s=10)
fit.rr.oracle.p$coef<-fit.rr.oracle.p$coef[c(p+1,1:p)]
cfs.rr.oracle.p <- my.unstandardize(fit.rr.oracle.p$coef, xz)
ps.rr.oracle.p <- fit.rr.oracle.p$predict[1:n]
lps.rr.oracle.p <- fit.rr.oracle.p$linear.predictors[1:n]
calib.p.rr.oracle.p <-  c(coef(glm(testY~c(testX[,1:(p+1)] %*% cfs.rr.oracle.p), family = "binomial"))[2])
cs.rr.oracle.p <- cindex(lps.rr.oracle.p, new.y)

##priorv=0.5
opt.rr.gl.05 <- 1/0.5
fit.rr.gl.05 <- logistr(df, formulas, penalize=pen, priorv=0.5, s=10)
fit.rr.gl.05$coef<-fit.rr.gl.05$coef[c(p+1,1:p)]
cfs.rr.gl.05 <- my.unstandardize(fit.rr.gl.05$coef, xz)
ps.rr.gl.05 <- fit.rr.gl.05$predict[1:n]
lps.rr.gl.05 <- fit.rr.gl.05$linear.predictors[1:n]
calib.p.rr.gl.05 <- c(coef(glm(testY~c(testX[,1:(p+1)] %*% cfs.rr.gl.05), family = "binomial"))[2])
cs.rr.gl.05 <- cindex(lps.rr.gl.05, new.y)

##priorv=2
opt.rr.gl.2 <- 1/2
fit.rr.gl.2 <- logistr(df, formulas, penalize=pen, priorv=2, s=10)
fit.rr.gl.2$coef <- fit.rr.gl.2$coef[c(p+1,1:p)]
cfs.rr.gl.2 <- my.unstandardize(fit.rr.gl.2$coef, xz)
ps.rr.gl.2 <- fit.rr.gl.2$predict[1:n]
lps.rr.gl.2 <- fit.rr.gl.2$linear.predictors[1:n]
calib.p.rr.gl.2 <- c(coef(glm(testY~c(testX[,1:(p+1)] %*% cfs.rr.gl.2), family = "binomial"))[2])
cs.rr.gl.2 <- cindex(lps.rr.gl.2, new.y)

#save results
coefs.nonoise <- c(cfs.f, cfs.rr.aic, cfs.rr.dev.1, cfs.rr.dev.g.1, cfs.rr.ce.1,
                   cfs.rr.perc.50, cfs.rr.perc.95, cfs.rr.oracle, cfs.rr.oracle.p,
                   cfs.rr.gl.05, cfs.rr.gl.2)
pnlty.nonoise <- c(opt.rr.aic, opt.rr.dev.1, opt.rr.dev.g.1, opt.rr.ce.1,
                   opt.rr.perc.50, opt.rr.perc.95, opt.rr.oracle, opt.rr.oracle.p,
                   opt.rr.gl.05, opt.rr.gl.2)
probs.nonoise <- c(ps.f, ps.rr.aic, ps.rr.dev.1, ps.rr.dev.g.1, ps.rr.ce.1,
                   ps.rr.perc.50, ps.rr.perc.95, ps.rr.oracle, ps.rr.oracle.p,
                   ps.rr.gl.05, ps.rr.gl.2)
linpred.nonoise <- c(lps.f, lps.rr.aic, lps.rr.dev.1, lps.rr.dev.g.1, lps.rr.ce.1,
                     lps.rr.perc.50, lps.rr.perc.95, lps.rr.oracle, lps.rr.oracle.p,
```

```
                     lps.rr.gl.05, lps.rr.gl.2)
calib.p.nonoise <- c(calib.p.f, calib.p.rr.aic, calib.p.rr.dev.1, calib.p.rr.dev.g.1, calib.p.rr.ce.1,
                     calib.p.rr.perc.50, calib.p.rr.perc.95, calib.p.rr.oracle, calib.p.rr.oracle.p,
                     calib.p.rr.gl.05, calib.p.rr.gl.2)
cstat.nonoise <- c(cs.f, cs.rr.aic, cs.rr.dev.1, cs.rr.dev.g.1, cs.rr.ce.1,
                   cs.rr.perc.50, cs.rr.perc.95, cs.rr.oracle, cs.rr.oracle.p,
                   cs.rr.gl.05, cs.rr.gl.2)


#Noise present
p=dim(x)[2]
xx=cbind(1,x)

##Firth

df<-data.frame(y, x)
formulas<-as.formula(paste("y~",paste(names(df)[-1] ,collapse="+"),sep=""))
firth<-logistf(formulas, df, flic=T)

cfs.f <- c(firth$coef)
ps.f <- c(firth$predict)
lps.f <- c(firth$linear.predictors)
calib.p.f <- c(coef(glm(testY~c(testX %*% cfs.f), family = "binomial"))[2])
cs.f <- cindex(lps.f, new.y)

###Ridge

#standardize covariates to zero mean and unit variance
xz<-my.std(xx,type=1)
xs<-xz$X

df<-data.frame(y, xs[,-1])
formulas<-as.formula(paste("y~",paste(names(df)[-1] ,collapse="+"),sep=""))

pen<-c(0,rep(1,p))

#LOOCV predicted probabilities
fit.opt.1<-profL2(y, xs[,-1], lambda1 = 0, minlambda2=1e-6, maxlambda2=100, steps=200,
                  minsteps=200, log=TRUE, fusedl = FALSE, positive = FALSE,  model =   "logistic" ,
                  fold=n, standardize = FALSE,  save.predictions = TRUE, trace = FALSE, plot = FALSE,
                  approximate = F)


prob_min = 1e-05
prob_max = 1 - prob_min

fit.opt.1$predictions <- lapply(fit.opt.1$predictions, function(x) pmin(pmax(x, prob_min), prob_max))

##AIC
aic <-  my.aic.ridge(l=fit.opt.1$lambda, y=y, X=xs, penalize=NULL)
coef.rr <- aic[[3]]
edf <- aic[[2]]
probs <- aic[[4]]

opt.rr.aic <- fit.opt.1$lambda[which.min(aic[[1]])]
fit.rr.aic <- logistr(df, formulas,penalize=pen, priorv=1/opt.rr.aic, s=10)
fit.rr.aic$coef <- fit.rr.aic$coef[c(p+1,1:p)]
cfs.rr.aic <- my.unstandardize(fit.rr.aic$coef, xz)
ps.rr.aic <- fit.rr.aic$predict[1:n]
lps.rr.aic <- fit.rr.aic$linear.predictors[1:n]
calib.p.rr.aic <- c(coef(glm(testY~c(testX %*% cfs.rr.aic), family = "binomial"))[2])
cs.rr.aic <- cindex(lps.rr.aic, new.y)

##D
dev.1 <- unlist(lapply(1:200, my.dev,fit.opt.1$predictions, y))
opt.rr.dev.1 <- fit.opt.1$lambda[which.min(dev.1)]
fit.rr.dev.1 <- logistr(df, formulas,penalize=pen, priorv=1/opt.rr.dev.1, s=10)
fit.rr.dev.1$coef <- fit.rr.dev.1$coef[c(p+1,1:p)]
cfs.rr.dev.1 <- my.unstandardize(fit.rr.dev.1$coef, xz)
ps.rr.dev.1 <- fit.rr.dev.1$predict[1:n]
lps.rr.dev.1 <- fit.rr.dev.1$linear.predictors[1:n]
calib.p.rr.dev.1 <- c(coef(glm(testY~c(testX %*% cfs.rr.dev.1), family = "binomial"))[2])
cs.rr.dev.1 <- cindex(lps.rr.dev.1, new.y)

##GCV
dev.g.1 <- unlist(lapply(1:200, my.dev.g, fit.opt.1$predictions, y, edf))
opt.rr.dev.g.1 <- fit.opt.1$lambda[which.min(dev.g.1)]
fit.rr.dev.g.1 <- logistr(df, formulas,penalize=pen, priorv=1/opt.rr.dev.g.1, s=10)
fit.rr.dev.g.1$coef <- fit.rr.dev.g.1$coef[c(p+1,1:p)]
cfs.rr.dev.g.1 <- my.unstandardize(fit.rr.dev.g.1$coef, xz)
ps.rr.dev.g.1 <- fit.rr.dev.g.1$predict[1:n]
lps.rr.dev.g.1 <- fit.rr.dev.g.1$linear.predictors[1:n]
calib.p.rr.dev.g.1 <- c(coef(glm(testY~c(testX %*% cfs.rr.dev.g.1), family = "binomial"))[2])
cs.rr.dev.g.1 <- cindex(lps.rr.dev.g.1, new.y)

##CE
```

```
ce.1 <- unlist(lapply(1:200, my.error, fit.opt.1$predictions, y))
opt.rr.ce.1 <- fit.opt.1$lambda[which.min(ce.1)]
fit.rr.ce.1<-logistr(df, formulas,penalize=pen, priorv=1/opt.rr.ce.1, s=10)
fit.rr.ce.1$coef<-fit.rr.ce.1$coef[c(p+1,1:p)]
cfs.rr.ce.1<-my.unstandardize(fit.rr.ce.1$coef,xz)
ps.rr.ce.1 <- fit.rr.ce.1$predict[1:n]
lps.rr.ce.1 <- fit.rr.ce.1$linear.predictors[1:n]
calib.p.rr.ce.1 <- c(coef(glm(testY~c(testX %*% cfs.rr.ce.1), family = "binomial"))[2])
cs.rr.ce.1 <- cindex(lps.rr.ce.1, new.y)

##repeated 10-fold CV
opt.dev.10=NULL
for(j in 1:50){
  fit.opt.10 <- profL2(y, xs[,-1], lambda1 = 0, minlambda2=1e-6, maxlambda2=100, steps=200,
                     minsteps=200, log=TRUE, fusedl = FALSE, positive = FALSE,  model =   "logistic",
                     fold=10, standardize = FALSE,  save.predictions = T, trace = FALSE,
                     plot = FALSE, approximate = F)
  fit.opt.10$predictions <- lapply(fit.opt.10$predictions, function(x) pmin(pmax(x, prob_min), prob_max))
  dev.10 <- unlist(lapply(1:200,my.dev,fit.opt.10$predictions,y))
  opt.dev.10[j] <- fit.opt.10$lambda[which.min(dev.10)]
}

##RCV50
opt.rr.perc.50 <- median(opt.dev.10)
fit.rr.perc.50 <- logistr(df, formulas, penalize=pen, priorv=1/opt.rr.perc.50, s=10)
fit.rr.perc.50$coef <- fit.rr.perc.50$coef[c(p+1,1:p)]
cfs.rr.perc.50 <- my.unstandardize(fit.rr.perc.50$coef, xz)
ps.rr.perc.50 <- fit.rr.perc.50$predict[1:n]
lps.rr.perc.50 <- fit.rr.perc.50$linear.predictors[1:n]
calib.p.rr.perc.50 <- c(coef(glm(testY~c(testX %*% cfs.rr.perc.50), family = "binomial"))[2])
cs.rr.perc.50 <- cindex(lps.rr.perc.50, new.y)

##RCV95
opt.rr.perc.95 <- quantile(opt.dev.10, c(0.95))
fit.rr.perc.95 <- logistr(df, formulas, penalize=pen, priorv=1/opt.rr.perc.95, s=10)
fit.rr.perc.95$coef<-fit.rr.perc.95$coef[c(p+1,1:p)]
cfs.rr.perc.95 <- my.unstandardize(fit.rr.perc.95$coef, xz)
ps.rr.perc.95 <- fit.rr.perc.95$predict[1:n]
lps.rr.perc.95 <- fit.rr.perc.95$linear.predictors[1:n]
calib.p.rr.perc.95 <- c(coef(glm(testY~c(testX %*% cfs.rr.perc.95), family = "binomial"))[2])
cs.rr.perc.95 <- cindex(lps.rr.perc.95, new.y)

##explanation oracle
oracle <- unlist(lapply(1:200, my.oracle, coef.rr, xz, beta, j=2))
opt.rr.oracle <- fit.opt.1$lambda[which.min(oracle)]
fit.rr.oracle <- logistr(df, formulas, penalize=pen, priorv=1/opt.rr.oracle, s=10)
fit.rr.oracle$coef<-fit.rr.oracle$coef[c(p+1,1:p)]
cfs.rr.oracle <- my.unstandardize(fit.rr.oracle$coef, xz)
ps.rr.oracle <- fit.rr.oracle$predict[1:n]
lps.rr.oracle <- fit.rr.oracle$linear.predictors[1:n]
calib.p.rr.oracle <- c(coef(glm(testY~c(testX %*% cfs.rr.oracle), family = "binomial"))[2])
cs.rr.oracle <- cindex(lps.rr.oracle, new.y)

##prediction oracle
oracle.p <- unlist(lapply(1:200, my.oracle.p, predictions=probs, ps=ps))
opt.rr.oracle.p <- fit.opt.1$lambda[which.min(oracle.p)]
fit.rr.oracle.p <- logistr(df, formulas, penalize=pen, priorv=1/opt.rr.oracle.p, s=10)
fit.rr.oracle.p$coef<-fit.rr.oracle.p$coef[c(p+1,1:p)]
cfs.rr.oracle.p <- my.unstandardize(fit.rr.oracle.p$coef, xz)
ps.rr.oracle.p <- fit.rr.oracle.p$predict[1:n]
lps.rr.oracle.p <- fit.rr.oracle.p$linear.predictors[1:n]
calib.p.rr.oracle.p <-  c(coef(glm(testY~c(testX %*% cfs.rr.oracle.p), family = "binomial"))[2])
cs.rr.oracle.p <- cindex(lps.rr.oracle.p, new.y)

##priorv=0.5
opt.rr.gl.05 <- 1/0.5
fit.rr.gl.05 <- logistr(df, formulas, penalize=pen, priorv=0.5, s=10)
fit.rr.gl.05$coef<-fit.rr.gl.05$coef[c(p+1,1:p)]
cfs.rr.gl.05 <- my.unstandardize(fit.rr.gl.05$coef, xz)
ps.rr.gl.05 <- fit.rr.gl.05$predict[1:n]
lps.rr.gl.05 <- fit.rr.gl.05$linear.predictors[1:n]
calib.p.rr.gl.05 <- c(coef(glm(testY~c(testX %*% cfs.rr.gl.05), family = "binomial"))[2])
cs.rr.gl.05 <- cindex(lps.rr.gl.05, new.y)

##priorv=2
opt.rr.gl.2 <- 1/2
fit.rr.gl.2 <- logistr(df, formulas, penalize=pen, priorv=2, s=10)
fit.rr.gl.2$coef <- fit.rr.gl.2$coef[c(p+1,1:p)]
cfs.rr.gl.2 <- my.unstandardize(fit.rr.gl.2$coef, xz)
ps.rr.gl.2 <- fit.rr.gl.2$predict[1:n]
lps.rr.gl.2 <- fit.rr.gl.2$linear.predictors[1:n]
calib.p.rr.gl.2 <- c(coef(glm(testY~c(testX %*% cfs.rr.gl.2), family = "binomial"))[2])
cs.rr.gl.2 <- cindex(lps.rr.gl.2, new.y)

#save results
coefs.noise <- c(cfs.f, cfs.rr.aic, cfs.rr.dev.1, cfs.rr.dev.g.1, cfs.rr.ce.1,
```

```
                 cfs.rr.perc.50, cfs.rr.perc.95, cfs.rr.oracle, cfs.rr.oracle.p,
                 cfs.rr.gl.05, cfs.rr.gl.2)
pnlty.noise <- c(opt.rr.aic, opt.rr.dev.1, opt.rr.dev.g.1, opt.rr.ce.1,
                 opt.rr.perc.50, opt.rr.perc.95, opt.rr.oracle, opt.rr.oracle.p,
                 opt.rr.gl.05, opt.rr.gl.2)
probs.noise <- c(ps.f, ps.rr.aic, ps.rr.dev.1, ps.rr.dev.g.1, ps.rr.ce.1,
                 ps.rr.perc.50, ps.rr.perc.95, ps.rr.oracle, ps.rr.oracle.p,
                 ps.rr.gl.05, ps.rr.gl.2)
linpred.noise <- c(lps.f, lps.rr.aic, lps.rr.dev.1, lps.rr.dev.g.1, lps.rr.ce.1,
                   lps.rr.perc.50, lps.rr.perc.95, lps.rr.oracle, lps.rr.oracle.p,
                   lps.rr.gl.05, lps.rr.gl.2)
calib.p.noise <- c(calib.p.f, calib.p.rr.aic, calib.p.rr.dev.1, calib.p.rr.dev.g.1, calib.p.rr.ce.1,
                   calib.p.rr.perc.50, calib.p.rr.perc.95, calib.p.rr.oracle, calib.p.rr.oracle.p,
                   calib.p.rr.gl.05, calib.p.rr.gl.2)
cstat.noise <- c(cs.f, cs.rr.aic, cs.rr.dev.1, cs.rr.dev.g.1, cs.rr.ce.1,
                 cs.rr.perc.50, cs.rr.perc.95, cs.rr.oracle, cs.rr.oracle.p,
                 cs.rr.gl.05, cs.rr.gl.2)

pars <- c(n, n.var, target.prop, beta, ys, ps, lps, cs, sep)

ires <- c(pars, coefs.nonoise, pnlty.nonoise, probs.nonoise, linpred.nonoise, calib.p.nonoise, cstat.nonoise,
          coefs.noise, pnlty.noise, probs.noise, linpred.noise, calib.p.noise, cstat.noise)

names(ires) <- c("n", "n.var", "target.prop", rep("beta", n.var+6),
                 "ys", rep("ps", n), rep("lps", n), "cs", "sep",

                 rep("cfs.f.nn", n.var+1), rep("cfs.rr.aic.nn", n.var+1), rep("cfs.rr.dev.1.nn", n.var+1),
                 rep("cfs.rr.dev.g.1.nn", n.var+1), rep("cfs.rr.ce.1.nn", n.var+1),
                 rep("cfs.rr.perc.50.nn", n.var+1), rep("cfs.rr.perc.95.nn", n.var+1),
                 rep("cfs.rr.oracle.nn", n.var+1), rep("cfs.rr.oracle.p.nn", n.var+1),
                 rep("cfs.rr.gl.05.nn", n.var+1), rep("cfs.rr.gl.2.nn", n.var+1),

                 "opt.rr.aic.nn", "opt.rr.dev.1.nn", "opt.rr.dev.g.1.nn", "opt.rr.ce.1.nn",
                 "opt.rr.perc.50.nn", "opt.rr.perc.95.nn", "opt.rr.oracle.nn", "opt.rr.oracle.p.nn",
                 "opt.rr.gl.05.nn", "opt.rr.gl.2.nn",

                 rep("ps.f.nn", n), rep("ps.rr.aic.nn", n), rep("ps.rr.dev.1.nn", n),
                 rep("ps.rr.dev.g.1.nn", n), rep("ps.rr.ce.1.nn", n), rep("ps.rr.perc.50.nn", n),
                 rep("ps.rr.perc.95.nn", n), rep("ps.rr.oracle.nn", n), rep("ps.rr.oracle.p.nn", n),
                 rep("ps.rr.gl.05.nn", n), rep("ps.rr.gl.2.nn", n),

                 rep("lps.f.nn", n), rep("lps.rr.aic.nn", n), rep("lps.rr.dev.1.nn", n),
                 rep("lps.rr.dev.g.1.nn", n), rep("lps.rr.ce.1.nn", n), rep("lps.rr.perc.50.nn", n),
                 rep("lps.rr.perc.95.nn", n), rep("lps.rr.oracle.nn", n), rep("lps.rr.oracle.p.nn", n),
                 rep("lps.rr.gl.05.nn", n), rep("lps.rr.gl.2.nn", n),

                 "calib.p.f.nn", "calib.p.rr.aic.nn", "calib.p.rr.dev.1.nn", "calib.p.rr.dev.g.1.nn",
                 "calib.p.rr.ce.1.nn", "calib.p.rr.perc.50.nn", "calib.p.rr.perc.95.nn",
                 "calib.p.rr.oracle.nn", "calib.p.rr.oracle.p.nn", "calib.p.rr.gl.05.nn",
                 "calib.p.rr.gl.2.nn",

                 "cstat.f.nn", "cstat.rr.aic.nn", "cstat.rr.dev.1.nn", "cstat.rr.dev.g.1.nn",
                 "cstat.rr.ce.1.nn", "cstat.rr.perc.50.nn", "cstat.rr.perc.95.nn", "cstat.rr.oracle.nn",
                 "cstat.rr.oracle.p.nn", "cstat.rr.gl.05.nn", "cstat.rr.gl.2.nn",

                 rep("cfs.f.n", n.var+6), rep("cfs.rr.aic.n", n.var+6), rep("cfs.rr.dev.1.n", n.var+6),
                 rep("cfs.rr.dev.g.1.n", n.var+6), rep("cfs.rr.ce.1.n", n.var+6),
                 rep("cfs.rr.perc.50.n", n.var+6), rep("cfs.rr.perc.95.n", n.var+6),
                 rep("cfs.rr.oracle.n", n.var+6), rep("cfs.rr.oracle.p.n", n.var+6),
                 rep("cfs.rr.gl.05.n", n.var+6), rep("cfs.rr.gl.2.n", n.var+6),

                 "opt.rr.aic.n", "opt.rr.dev.1.n", "opt.rr.dev.g.1.n", "opt.rr.ce.1.n",
                 "opt.rr.perc.50.n", "opt.rr.perc.95.n", "opt.rr.oracle.n", "opt.rr.oracle.p.n",
                 "opt.rr.gl.05.n", "opt.rr.gl.2.n",

                 rep("ps.f.n", n), rep("ps.rr.aic.n", n), rep("ps.rr.dev.1.n", n),
                 rep("ps.rr.dev.g.1.n", n), rep("ps.rr.ce.1.n", n), rep("ps.rr.perc.50.n", n),
                 rep("ps.rr.perc.95.n", n), rep("ps.rr.oracle.n", n),
                 rep("ps.rr.oracle.p.n", n), rep("ps.rr.gl.05.n", n), rep("ps.rr.gl.2.n", n),

                 rep("lps.f.n", n), rep("lps.rr.aic.n", n), rep("lps.rr.dev.1.n", n),
                 rep("lps.rr.dev.g.1.n", n), rep("lps.rr.ce.1.n", n), rep("lps.rr.perc.50.n", n),
                 rep("lps.rr.perc.95.n", n), rep("lps.rr.oracle.n", n), rep("lps.rr.oracle.p.n", n),
                 rep("lps.rr.gl.05.n", n), rep("lps.rr.gl.2.n", n),

                 "calib.p.f.n", "calib.p.rr.aic.n", "calib.p.rr.dev.1.n", "calib.p.rr.dev.g.1.n",
                 "calib.p.rr.ce.1.n", "calib.p.rr.perc.50.n", "calib.p.rr.perc.95.n", "calib.p.rr.oracle.n",
                 "calib.p.rr.oracle.p.n", "calib.p.rr.gl.05.n", "calib.p.rr.gl.2.n",

                 "cstat.f.n", "cstat.rr.aic.n", "cstat.rr.dev.1.n", "cstat.rr.dev.g.1.n", "cstat.rr.ce.1.n",
                 "cstat.rr.perc.50.n", "cstat.rr.perc.95.n", "cstat.rr.oracle.n", "cstat.rr.oracle.p.n",
                 "cstat.rr.gl.05.n", "cstat.rr.gl.2.n")

ires
```

```
                    }
}


# Data example ####################################################################################

load("data.Rdata")

dd <- dd[with(dd, order(id)), ]
size = 275

set.seed(234)
train.id <- sample(1:nrow(dd), size=size)
train <- dd[train.id,]
test <- dd[-train.id, ]

table(train$outcome); prop.table(table(train$outcome))
table(test$outcome); prop.table(table(test$outcome))

y <- train$outcome
x <- as.matrix(train[,c("age", "sex", "ITM", "APGAR", "cron.disease", "fall", "lonlines", "health", "pain")])
x <- apply(x, 2, as.numeric)

testX <- cbind(1, as.matrix(test[,c("age", "sex", "ITM", "APGAR", "cron.disease", "fall", "lonlines", "health", "pain")]))
testX <- apply(testX, 2, as.numeric)

#standardize
xz<-my.std(cbind(1, x), type=1)
xs<-xz$X

df<-data.frame(y, xs[,-1])
formulas<-as.formula(paste("y~",paste(names(df)[-1] ,collapse="+"),sep=""))

p=ncol(x)
pen<-c(0,rep(1, ncol(x)))

fit.opt.1<-profL2(y, xs[,-1], lambda1 = 0, minlambda2=1e-6, maxlambda2=100, steps=200, minsteps=200, log=TRUE,
                  fusedl = FALSE, positive = FALSE,  model =   "logistic" , fold=length(y),
                  standardize = F,  save.predictions = T, trace = FALSE, plot = FALSE, approximate = F)

prob_min <- 1e-05
prob_max <- 1 - prob_min

fit.opt.1$predictions <- lapply(fit.opt.1$predictions, function(x) pmin(pmax(x, prob_min), prob_max))

##Firth
cfs.f <- coef(logistf(outcome~age+sex+as.numeric(ITM)+APGAR+as.numeric(cron.disease)+fall+lonlines+health+pain,
                      data=train, flic = T))
calib.p.f <- c(coef(glm(test$outcome~(testX %*% cfs.f), family = "binomial"))[2])

##AIC
aic = my.aic.ridge(l=fit.opt.1$lambda, y=y, X=xs, penalize=NULL)
coef.rr <- aic[[3]]
edf <- aic[[2]]
opt.rr.aic <- fit.opt.1$lambda[which.min(aic[[1]])]
fit.rr.aic <- logistr(df, formulas,penalize=pen, priorv=1/opt.rr.aic, s=10)
fit.rr.aic$coef <- fit.rr.aic$coef[c(p+1,1:p)]
cfs.rr.aic <- my.unstandardize(fit.rr.aic$coef, xz)
calib.p.rr.aic <- c(coef(glm(test$outcome~(testX %*% cfs.rr.aic), family = "binomial"))[2])


##D
dev.1 <- unlist(lapply(1:200, my.dev, fit.opt.1$predictions, y))
opt.rr.dev.1 <- fit.opt.1$lambda[which.min(dev.1)]
fit.rr.dev.1 <- logistr(df, formulas,penalize=pen, priorv=1/opt.rr.dev.1, s=10)
fit.rr.dev.1$coef <- fit.rr.dev.1$coef[c(p+1,1:p)]
cfs.rr.dev.1 <- my.unstandardize(fit.rr.dev.1$coef, xz)
calib.p.rr.dev.1 <- c(coef(glm(test$outcome~(testX %*% cfs.rr.dev.1), family = "binomial"))[2])


##GCV
dev.g.1 <- unlist(lapply(1:200, my.dev.g, fit.opt.1$predictions, y, edf))
opt.rr.dev.g.1 <- fit.opt.1$lambda[which.min(dev.g.1)]
fit.rr.dev.g.1 <- logistr(df, formulas,penalize=pen, priorv=1/opt.rr.dev.g.1, s=10)
fit.rr.dev.g.1$coef <- fit.rr.dev.g.1$coef[c(p+1,1:p)]
cfs.rr.dev.g.1 <- my.unstandardize(fit.rr.dev.g.1$coef, xz)
calib.p.rr.dev.g.1 <- c(coef(glm(test$outcome~(testX %*% cfs.rr.dev.g.1), family = "binomial"))[2])


##CE
ce.1 <- unlist(lapply(1:200, my.error, fit.opt.1$predictions, y))
opt.rr.ce.1 <- fit.opt.1$lambda[which.min(ce.1)]
fit.rr.ce.1 <- logistr(df, formulas,penalize=pen, priorv=1/opt.rr.ce.1, s=10)
fit.rr.ce.1$coef <- fit.rr.ce.1$coef[c(p+1,1:p)]
cfs.rr.ce.1 <- my.unstandardize(fit.rr.ce.1$coef,xz)
calib.p.rr.ce.1 <- c(coef(glm(test$outcome~(testX %*% cfs.rr.ce.1), family = "binomial"))[2])
```

```
##IP
fit.rr.gl.05 <- logistr(df, formulas, penalize=pen, priorv=0.5, s=10)
fit.rr.gl.05$coef<-fit.rr.gl.05$coef[c(p+1,1:p)]
cfs.rr.gl.05 <- my.unstandardize(fit.rr.gl.05$coef, xz)
calib.p.rr.gl.05 <- c(coef(glm(test$outcome~(testX %*% cfs.rr.gl.05), family = "binomial"))[2])


##WP
fit.rr.gl.2 <- logistr(df, formulas, penalize=pen, priorv=2, s=10)
fit.rr.gl.2$coef <- fit.rr.gl.2$coef[c(p+1,1:p)]
cfs.rr.gl.2 <- my.unstandardize(fit.rr.gl.2$coef, xz)
calib.p.rr.gl.2 <- c(coef(glm(test$outcome~(testX %*% cfs.rr.gl.2), family = "binomial"))[2])

##RCV
opt.dev.10=NULL
for(j in 1:50){
  fit.opt.10 <- profL2(y, xs[,-1], lambda1 = 0, minlambda2=1e-6, maxlambda2=100, steps=200, minsteps=200, log=TRUE,
                      fusedl = FALSE, positive = FALSE,  model =   "logistic" , fold=10,
                      standardize = FALSE,  save.predictions = T, trace = FALSE, plot = FALSE, approximate = F)
  fit.opt.10$predictions <- lapply(fit.opt.10$predictions, function(x) pmin(pmax(x, prob_min), prob_max))
  dev.10 <- unlist(lapply(1:200, my.dev, fit.opt.10$predictions, y))
  opt.dev.10[j] <- fit.opt.10$lambda[which.min(dev.10)]
}

opt.rr.perc.50 <- median(opt.dev.10)
fit.rr.perc.50 <- logistr(df, formulas, penalize=pen, priorv=1/opt.rr.perc.50, s=10)
fit.rr.perc.50$coef <- fit.rr.perc.50$coef[c(p+1,1:p)]
cfs.rr.perc.50 <- my.unstandardize(fit.rr.perc.50$coef, xz)
calib.p.rr.perc.50 <- c(coef(glm(test$outcome~(testX %*% cfs.rr.perc.50), family = "binomial"))[2])

opt.rr.perc.95 <- quantile(opt.dev.10, c(0.95))
fit.rr.perc.95 <- logistr(df, formulas, penalize=pen, priorv=1/opt.rr.perc.95, s=10)
fit.rr.perc.95$coef<-fit.rr.perc.95$coef[c(p+1,1:p)]
cfs.rr.perc.95 <- my.unstandardize(fit.rr.perc.95$coef, xz)
ps.rr.perc.95 <- fit.rr.perc.95$predict[1:n]
lps.rr.perc.95 <- fit.rr.perc.95$linear.predictors[1:n]
calib.p.rr.perc.95 <- c(coef(glm(test$outcome~(testX %*% cfs.rr.perc.95), family = "binomial"))[2])
```
